# Peroxynitrite-Activated Conditional Chemical Probe for the Affinity-Based Protein Modification

**DOI:** 10.1021/acs.analchem.4c05580

**Published:** 2025-07-10

**Authors:** Chien-Chi Wu, Szu-Hsien Lee, Ting-Ju Huang, Jing-Cyun Lin, Yueh-Hsun Lu, Shu-Pao Wu, Kui-Thong Tan

**Affiliations:** † Department of Chemistry, 34881 National Tsing Hua University, 101 Section 2, Kuang-Fu Road, Hsinchu 300044, Taiwan; ‡ Department of Medicinal and Applied Chemistry, Kaohsiung Medical University, Kaohsiung 80708, Taiwan; § Department of Applied Chemistry, 34914 National Yang Ming Chiao Tung University, Hsinchu 30010, Taiwan

## Abstract

Affinity-based protein labeling chemical probes are invaluable tools for the selective chemical modification of native proteins. This protein labeling strategy generally relies on “always ON” reactive electrophiles to achieve protein modification. However, reactive electrophiles are also the causes of probe instability and nonselective labeling in complex biological environments. In this paper, we introduce a conditional-activated affinity-based protein labeling strategy in which the probe is activated by peroxynitrite, triggering a labeling reaction with the target protein located in the near proximity. This conditionally activated protein labeling probe was synthesized with a peroxynitrite-responsive acyl hydrazide, a Cy5 fluorescent dye, and a sulfonamide ligand for recognition with human carbonic anhydrase (hCA) protein. We showed that the specific and rapid labeling of hCA can be achieved in the presence of peroxynitrite in living cells and in vivo. Long-term imaging of the target protein in vivo is possible due to the formation of a stable covalent bond. Furthermore, the probe can also be used to detect peroxynitrite secreted from macrophage cells. As compared with the previous reactive oxygen species-activated probes based on the formation of quinone methide intermediate, our hydrazide probe can be easily synthesized and is more resistant to self-hydrolysis in aqueous solutions. We anticipate that such a condition-responsive reagent will become an invaluable tool for research involving the chemical modification of native proteins under oxidative stress conditions.

## Graphical abstract

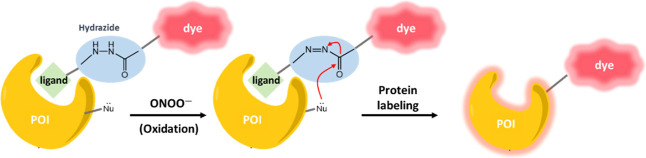

## Introduction

Selective chemical modification of the protein of interest (POI) is crucial in many biological and medical studies of protein functions, expression levels, and cellular localization, as well as the development of new biopharmaceuticals.^1–4^ Currently, genetically encoded tags and affinity-based chemical probes are the two main approaches employed for the selective chemical modification of POI. While genetically encoded methodologies using self-labeling proteins, enzyme-mediated proximity labeling, or genetic code expansion strategies have been highly effective for specific and sensitive labeling of POI in very complex biological contexts, these methods require prior genetic manipulations of the cells, which might disturb the cellular physiology, causing undesired artifacts in the downstream analysis.^5–8^ In contrast, affinity-based protein labeling probes decorated with a wide range of small molecules, such as fluorescent dyes, biotin, or drugs, are an invaluable alternative for the selective chemical modification of native proteins in complex biological samples. Recent progress in such affinity-based chemical probes generally relies on chemically reactive electrophiles or light-activated functional groups.^2,9–13^ These protein labeling probe designs have been applied in protein imaging, proteomics analysis, biosensor construction, and other applications to reveal many fundamental biological phenomena. However, the reactive nature of such electrophiles and the requirements of short-wavelength light for probe activation are also the cause of many problems associated with probe instability, target protein selectivity, labeling sensitivity, and cell toxicity.^14–16^

To overcome the existing problems of protein labeling probes, the concept of conditionally responsive protein labeling chemical probes was developed, in which the probe is activated in response to a stimulant in cells, thereby initiating the labeling reaction with the nearby proteins. Currently, hypoxia microenvironments,^17,18^ hydrogen peroxide,^19,20^ nitric oxide,^21^ and metal ions^22–24^ have been used as stimulants to trigger these conditionally responsive protein labeling probes. These probes have been applied in the study of metal ion homeostasis, the investigation of localized subproteomes under the target cellular conditions, and the imaging of brain cells and mouse organs. There are numerous advantages to developing conditionally responsive protein labeling probes. The probes allow the protein labeling events to take place on demand, which can potentially reduce background signal to provide better protein detection sensitivity. Furthermore, as the reactive electrophiles are not formed before activation, the problems due to probe instability in complex biological environments can be minimized. Despite several years of devoted developments, almost all the current conditionally responsive probes used latent quinone methide^19,20,25,26^ or reactive acyl imidazole^22–24^ sources for the covalent trapping and nonspecific labeling of the surrounding proteins upon activation. While this strategy can facilitate the labeling of as many proteins as possible for proteomic analysis, high protein concentrations are generally required for the probes to achieve effective protein labeling due to the absence of proximity effects.

In this paper, we report a new conditionally responsive protein labeling probe strategy based on an acyl hydrazide precursor that can be oxidized and activated by peroxynitrite (ONOO^–^) for the subsequent affinity-based labeling of the target proteins in close proximity (Figure 1). To demonstrate this affinity-based conditionally responsive protein labeling concept, we synthesized probe **1**, which consists of an ONOO^–^-responsive acyl hydrazide, a Cy5 fluorescent dye, and a sulfonamide ligand for recognition with human carbonic anhydrase (hCA) protein. It is known that primary sulfonamide binds selectively and strongly with hCA with nanomolar (nM) binding affinity.^27,28^ Upon formation of an acyl diazo electrophile by ONOO^–^ oxidation, protein labeling can take place via the proximity effect. Subsequently, the Cy5-labeled hCA protein can be detected by SDS-PAGE or imaging. We showed that specific and rapid hCA protein labeling can be achieved with probe **1** in the presence of ONOO^–^ in living cells and mice. As the probe is ONOO^–^-responsive, we also demonstrated that the probe can be used to detect the secretion of ONOO^–^ from macrophage cells. Previously, acyl hydrazide has been applied in fluorescent turn-on chemical probes for the rapid detection of peroxynitrite.^29,30^ In addition to the sensing applications, acyl hydrazide can also be used as a traceless safety-catch linker in solid-phase peptide synthesis^31,32^ or as a conjugation reagent to react with the carbonyl group.^33,34^

**Figure 1:**
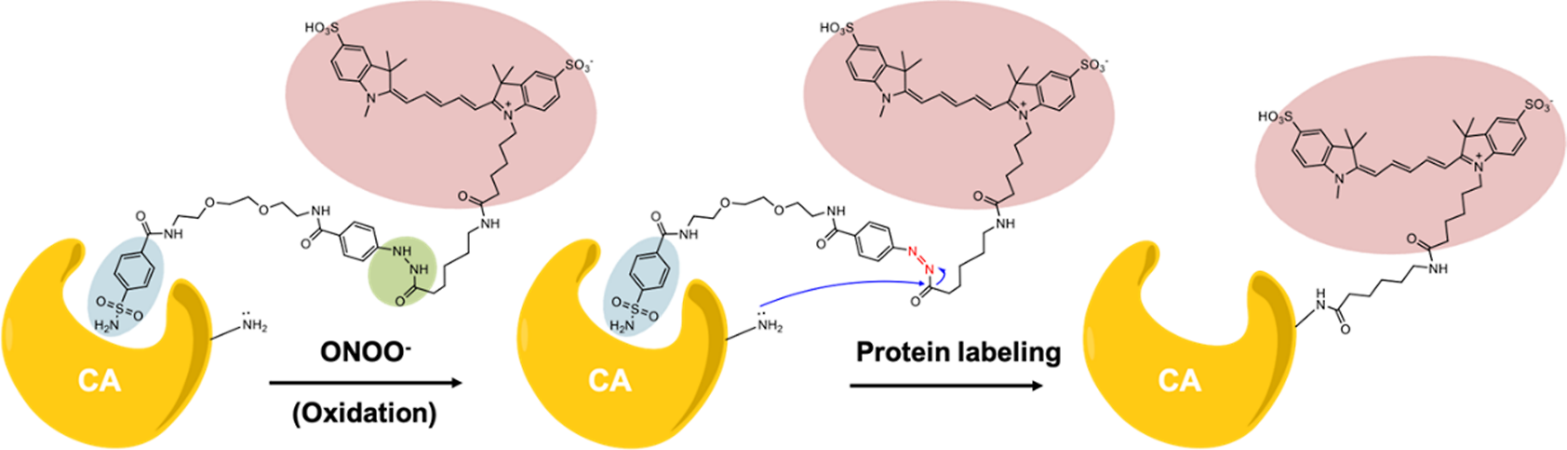
Schematic illustration of the peroxynitrite-activated affinity-based protein labeling strategy for the selective covalent modification of carbonic anhydrase protein (CA) with probe **1**.

## Experimental Section

### Fluorescence Imaging of Living A549 and MCF7 Cells

A549 and MCF7 cells were maintained in DMEM and MEM medium, respectively, supplemented with 10% FBS and 1% penicillin–streptomycin. 2 × 10^4^ cells were seeded in 8-well chamber slides (Ibidi GmbH, Germany) and cultured for 24 h at 37 °C in air with 5% CO_2_. The cells were washed three times with DMEM or MEM. The nucleus was stained with 0.1 μM Hoechst 34580 for 20 min. 1 μM probe **1** and 100 μM SIN-1 were added and incubated with the cells for 30 min in DMEM or MEM at 37 °C. After washing three times with the medium, live-cell imaging experiments were carried out using a laser scanning confocal microscope (Zeiss LSM 700, Germany). The fluorescent images were taken using a 639 nm laser and an LP640 emission filter. For Hoechst, a 405 nm laser and SP490 emission filter were used. The images were processed using ZEN 2009 Light Edition software (Zeiss, Germany). The fluorescence intensities were quantified using ImageJ software.

### Detection of the hCA Protein Residue from Cell Lysates

A549 and MCF7 cells (2 × 10^6^ cells) were seeded in a 100 mm dish and cultured in DMEM or MEM medium, respectively, with 10% FBS and 1% penicillin–streptomycin for 24 h at 37 °C. The cells were washed three times with serum-free medium and cultured for an additional 18 h in serum-free medium. The cells were labeled with 1 μM probe **1** in the presence of 100 μM SIN-1 for 30 min at 37 °C. For the analysis of protein residue on the cell surface, the cells were washed three times with cold PBS and lysed by using Pierce IP lysis buffer containing 1% halt protease and phosphatase inhibitor cocktail. The lysates were collected and centrifuged at 14000*g* for 10 min. The supernatant was mixed with 4× loading buffer (pH 6.8, 250 mM Tris–HCl, 40% glycerol, 8% SDS, 20% DTT). The samples were resolved by 10% SDS-PAGE gel and detected using the ChemiDoc Touch Imaging System (Biorad).

### Detection of Endogenous ONOO^–^ from the RAW264.7 Culture Medium

RAW264.7 cells (2 × 10^6^ cells) were seeded in a 100 mm dish and cultured in DMEM medium with 10% FBS and 1% penicillin–streptomycin for 24 h at 37 °C. The cells were washed three times with serum-free medium and cultured for an additional 18 h in serum-free medium. The cells were incubated with 1 μM probe **1**, 2 μM hCA II for the indicated times at 37 °C. 1.5 mL of culture media was collected, resolved by 10% SDS-PAGE gel, and detected using the ChemiDoc Touch Imaging System (Biorad).

### In Vivo Fluorescent Imaging Experiments

Four-week-old BALB/c nude mice were purchased from BioLASCO, Taiwan, and subsequently injected with 5 × 10^6^ A549 cells via a subcutaneous injection. All animal experiments were approved by National Yang Ming Chiao Tung University and conducted according to the manual of the National Laboratory Animal Center in Taiwan. Fluorescence images of the live mice and their dissected organs were then acquired using the Caliper IVIS Spectrum System equipped with an XGI-8 Anesthesia System. The stock solutions of control **2** and probe **1** were prepared in DMF to a concentration of 1 mM. SIN-1 was first dissolved in water to a concentration of 1 mM. All substances were diluted to the required working concentration by 10 mM PBS (pH 7.4). For intraperitoneal injection experiments, a 200 μL mixture of probe **1** (50 μM) and SIN-1 (500 μM) was intraperitoneally injected into the mouse. Fluorescence images were captured every 20 min for a duration of 2 h. After sacrificing the mouse, the organs were excised, and fluorescence images were taken using IVIS. For the intratumoral injection experiments, the tumor region of the mouse was injected with either the probe **1** (50 μM, 100 μL) only or a 100 μL mixture of **1** (50 μM) and SIN-1 (500 μM). Fluorescence images were taken every 20 min and lasted 100 min. Additionally, the tumor regions were injected with a control **2** (50 μM, 100 μL), and the fluorescence images were captured every 20 min and lasted 100 min. After 6 days of the intratumoral injection, the fluorescence images of the mice were captured again by an IVIS system.

## Results and Discussion

### Peroxynitrite-Activation of Probe 1 for the Labeling of Recombinant Human Carbonic Anhydrase

In general, 10 μM **1** was reacted with 20 μM purified recombinant human carbonic anhydrase (hCAII) and 100 μM ONOO^–^ generator 3-morpholinylsydnonimine chloride (SIN-1) for 30 min in pH 7.4 PBS buffer at 37 °C.^35^ Subsequently, the mixture was diluted 10-fold and analyzed using SDS-PAGE. The in-gel fluorescence images showed that an obvious fluorescent band was observed when the probe was treated together with hCAII and SIN-1 (Figures 2a and S1). In contrast, a weak fluorescent band was detected in the absence of SIN-1 or in the presence of the hCAII inhibitor ethoxzolamide (EZA). Probe **1** is not a fluorescence turn-on probe, as it does not exhibit a significant change in fluorescence intensity when labeled with hCAII (Figure S2). The labeling of the hCAII protein was not affected by the presence of other small nonsulfonamide inhibitors, as well as by 1 mM glycine and 100 μM glutathione (Figure S3). The protein labeling was significantly suppressed in the presence of ascorbic acid, likely due to the antioxidant’s reducing effect on the reactive intermediate (Figure S4). pH scanning indicated that the labeling of hCA occurred efficiently at neutral and basic pH, but it was less effective at acidic pH (Figure S5). The addition of SIN-1 and hCAII to control probe **2**, which does not contain a sulfonamide ligand, did not produce a fluorescent band in the SDS-PAGE gel (Figure S6). We also synthesized control probe **3**, which consists of a Cy5 fluorophore directly conjugated to sulfonamide but lacks the hydrazide moiety. In-gel fluorescence and live-cell imaging results showed that **3** failed to label hCA proteins with or without SIN-1 (Figure S7). These results indicate that this labeling reaction is stimulated by ONOO^–^ and is caused by a specific interaction between hCAII and the sulfonamide ligand.

**Figure 2:**
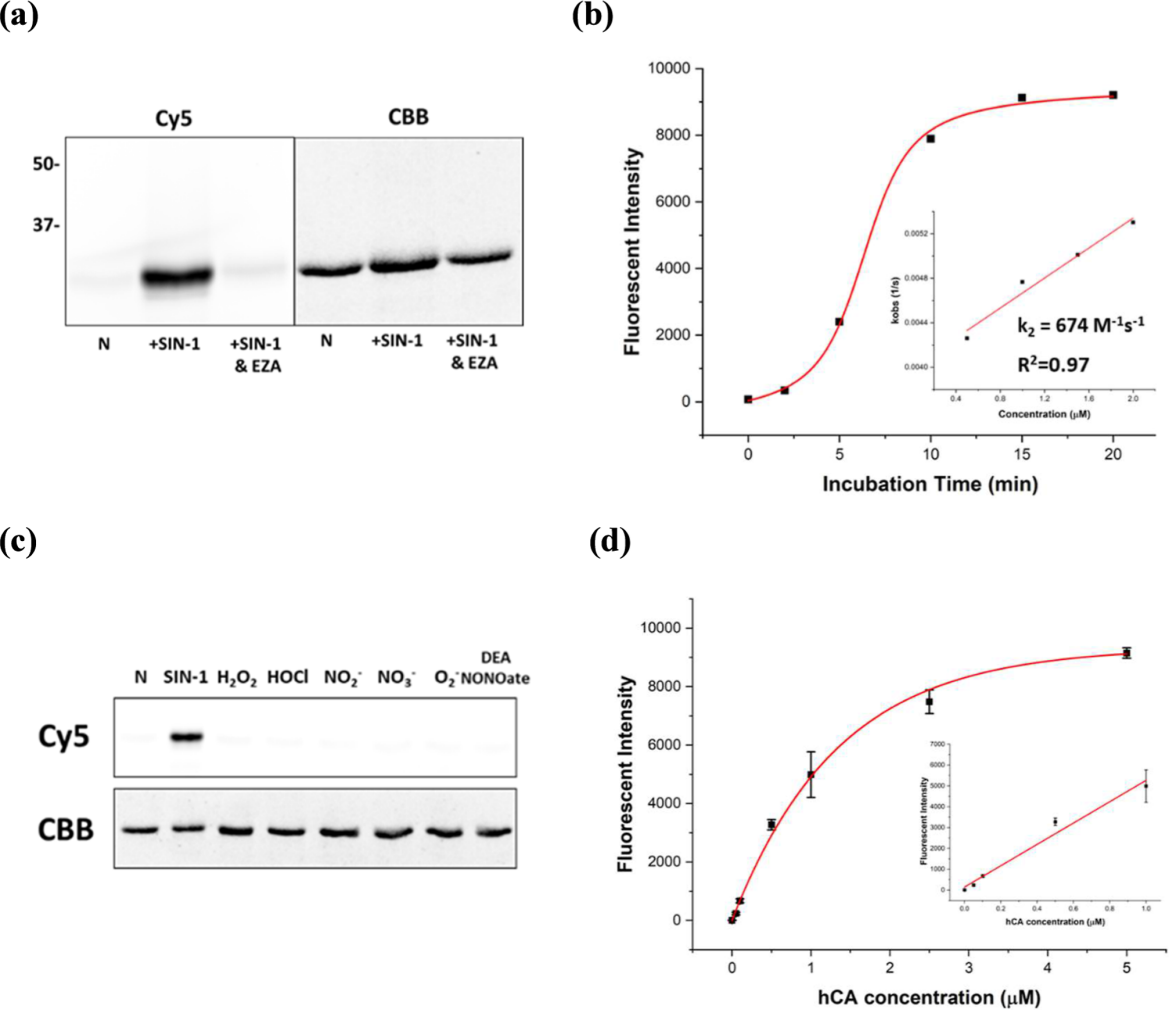
Peroxynitrite activation of probe **1** for the labeling of Cy5 fluorescent dye on hCAII. (a) SDS-PAGE in-gel fluorescent analysis of the hCAII labeling reaction with **1** in the absence or presence of 100 μM SIN-1 and EZA at 37 °C in pH 7.4 PBS buffer for 30 min. (b) Reaction time course of hCAII with **1** in the presence of 100 μM SIN-1 at 37 °C in pH 7.4 PBS buffer. The plot of apparent reaction rate constant *k*_obs_ (s^–1^) was shown in the inset. (c) Selectivity test of hCAII labeling with **1** in the presence of different oxidants (100 μM). *N* = without oxidant. (d) hCAII protein labeling sensitivity test in the presence of **1** and 100 μM SIN-1 at 37 °C in pH 7.4 PBS buffer for 30 min. The linear range was estimated from 0 to 1 μM hCAII (*R* ^2^ = 0.97).

The stability of the oxidized acyl diazo group was evaluated using HPLC. The reactive group is resistant to hydrolysis and remains intact after incubation with 100 μM SIN-1 in PBS buffer at 37 °C for 3 h (Figure S8). In comparison, the quinone methide electrophile has been reported to hydrolyze slowly in aqueous solutions.^20^ The labeling efficiency was determined to be approximately 27% by using HPLC (Figure S9). For the reaction kinetics, we observed rapid labeling of hCAII with probe **1**, and the reaction can be accomplished after 15 min of incubation (Figures 2b and S10). By varying the hCAII concentrations in the reaction, we determined the second-order rate constant (*k*_2_) to be approximately 674 M^–1^ s^–1^. The *k*_2_ value for probe **1** is comparable to the previous affinity-based protein labeling probes employing tosyl, alkyloxyacyl imidazole, dibromophenyl benzoate, and difluorophenyl ester, which exhibit *k*_2_ values ranging from 45 to 590  M^–1^ s^–1^.^36,37^ Given the known rapid reaction between peroxynitrite and hydrazide moieties, the electrophilic reaction between hCAII and the oxidized acyl diazo intermediate is probably the rate-limiting step in the overall labeling process.^38,39^

We proceeded to study the protein labeling specificity by performing the labeling reaction in the presence of other nontarget proteins. ONOO^–^-activated probe **1** exhibits a highly specific labeling reaction with hCAII but not with other nontarget proteins or in the presence of 10% fetal bovine serum (FBS), which contains 4.2 mg/mL of proteins (Figure S11). Next, we tested the selectivity of probe activation with various oxidative or reductive molecules. Of these tested molecules, only the addition of SIN-1 produced an intense fluorescent band in the SDS-PAGE gel (Figures 2c and S12). It is important to note that labeling of hCAII protein is not affected even in the presence of 5 mM H_2_O_2_ or DEA NoNOate, which is a NO donor (Figure S13).^40^ These results suggest that compound **1** is activated specifically by ONOO^–^. We also examined the minimum concentration of SIN-1 required to activate the hydrazide probe and the hCAII protein labeling sensitivity. By incubating 1 μM **1** with decreasing concentrations of hCAII or SIN-1 for 30 min at 37 °C, we determined that at least 0.32 μM SIN-1 was required to activate the probe, and the theoretical detection limit (LOD) for hCAII was estimated to be approximately 1.6 nM (Figures 2d and S14). Thus, **1** can be a robust ONOO^–^-responsive chemical probe for the rapid, sensitive, and specific labeling of hCA protein.

### Peroxynitrite-Activation of Probe 1 for the Labeling of Endogenous Transmembrane Human Carbonic Anhydrase Isozymes

To show that **1** can be used to label endogenous proteins, 1 μM **1** was incubated with MCF7 and A549 living cells for 30 min at 37 °C in the absence or presence of 100 μM SIN-1. The cell images were taken using a confocal laser scanning microscope after removing the unreacted probe and reagents by washing. We observed obvious fluorescence on the surface of the A549 and MCF7 cells (Figure 3a). In contrast, only very weak background fluorescence was detected when the cells were not treated with SIN1 or in the presence of 100 μM EZA. On the other hand, CHO cells, which do not express endogenous hCA protein on their surface, were not labeled by probe **1** in the presence of SIN-1 (Figure S15). To further characterize the labeling reaction, the **1**-labeled cell lysates were analyzed using SDS-PAGE in-gel fluorescence imaging. Two fluorescent bands with approximately 44 and 50 kDa sizes were detected on the A549 and MCF7 cell lysates, respectively (Figure 3b). The apparent molecular weight of these two bands corresponds closely with the sizes of hCAIX and hCAXII reported in literature, which indicate that MCF7 cells express 44 kDa hCAIX and A549 cells express 50 kDa hCAXII as the major extracellular hCA isozymes.^41–43^ Strong fluorescence on the cell surface is observed following treatment with SIN-1, but not with other oxidants (Figure S16). Thus, our ONOO^–^-activated protein labeling probe strategy can be applied for the rapid and selective analysis of extracellular hCA in cell lysates and living cells.

**Figure 3:**
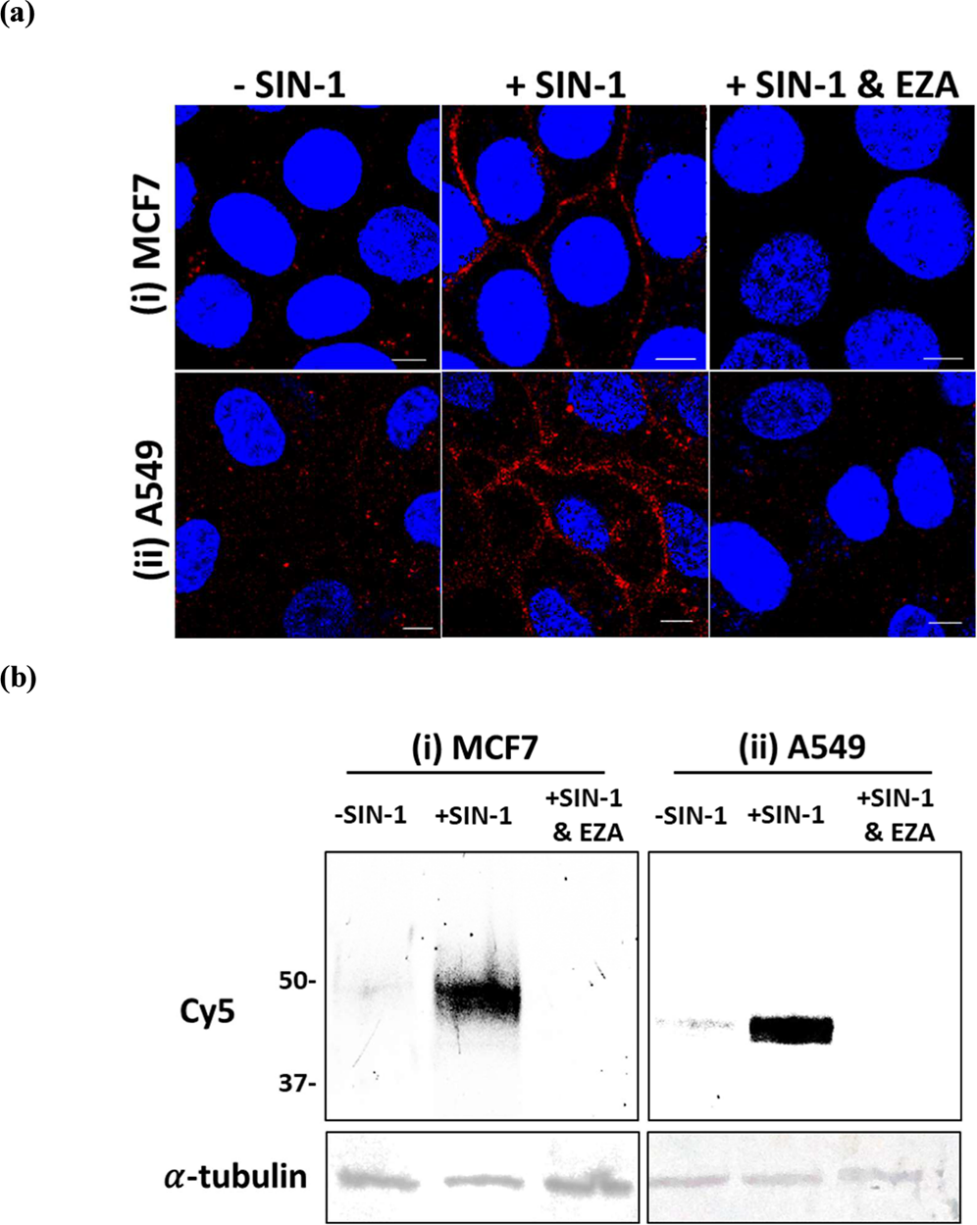
Activation of **1** by 100 μM SIN-1 for the labeling of native transmembrane hCA isozymes in living cells. (a) Fluorescent images of (i) MCF7 and (ii) A549 cells. Cy5 and Hoechst 33342 are shown in red and blue, respectively. Scale bar: 20 μm. (b) SDS-PAGE in-gel fluorescence analyses of the **1**-labeled MCF7 and A549 cell lysates.

### Labeling of Endogenous Carbonic Anhydrase after Hypoxia-Induced and Ectodomain Shedding Stimulation

It has been reported in the literature that expression of hCAXII in many tumor cell lines can be upregulated under hypoxic conditions or in the presence of hypoxia-mimetic agents, such as CoCl_2_ or deferoxamine mesylate.^44^ These hypoxia-mimetic agents can block the hypoxia-inducible factor (HIF-1α) degradation to induce artificial hypoxia conditions.^45^ On the other hand, the level of hCAXII can also be regulated by proteolytic cleavage, in which extracellular protein fragments are hydrolyzed and released into extracellular media.^41,46,47^ This process, which is termed ectodomain shedding, is a form of limited proteolysis catalyzed by various metalloproteases.^48,49^ Phosphokinase C (PKC) activation is one of the pathways to accelerate the ectodomain shedding event. Therefore, a PKC activator, e.g., phorbol 12-myristate 13-acetate (PMA), can also be used to accelerate ectodomain shedding.

To further demonstrate the applications of this ONOO^–^-responsive hydrazide group as a reactive module for biological studies, probe **1** was used to analyze the up- and down-regulation expression of hCAXII after hypoxia-induced and ectodomain shedding stimulation. To induce a hypoxic environment, A549 cells were incubated with 200 μM CoCl_2_ at 37 °C in different time intervals. The cells were washed with DMEM medium and incubated with 1 μM **1** and 100 μM SIN-1 for 30 min. Consistent with the literature reports, the hCAXII expression levels increased after prolonged incubation with CoCl_2_, and saturated steady-state was reached after 36 h (Figures 4a and S17). On the other hand, we observed a gradual decrease in the hCAXII expression levels for the A549 cells incubated with 10 μM PMA, which can be used for the activation of metalloprotease (Figures 4b and S18). These results imply that hCAXII protein turnover can be significantly affected under hypoxia and metalloprotease-activated conditions. The CCK-8 cytotoxicity assay showed that the incubation of the tested reagents and the probe at the specified concentrations did not result in significant toxicity to the cells (Figure S19).

**Figure 4:**
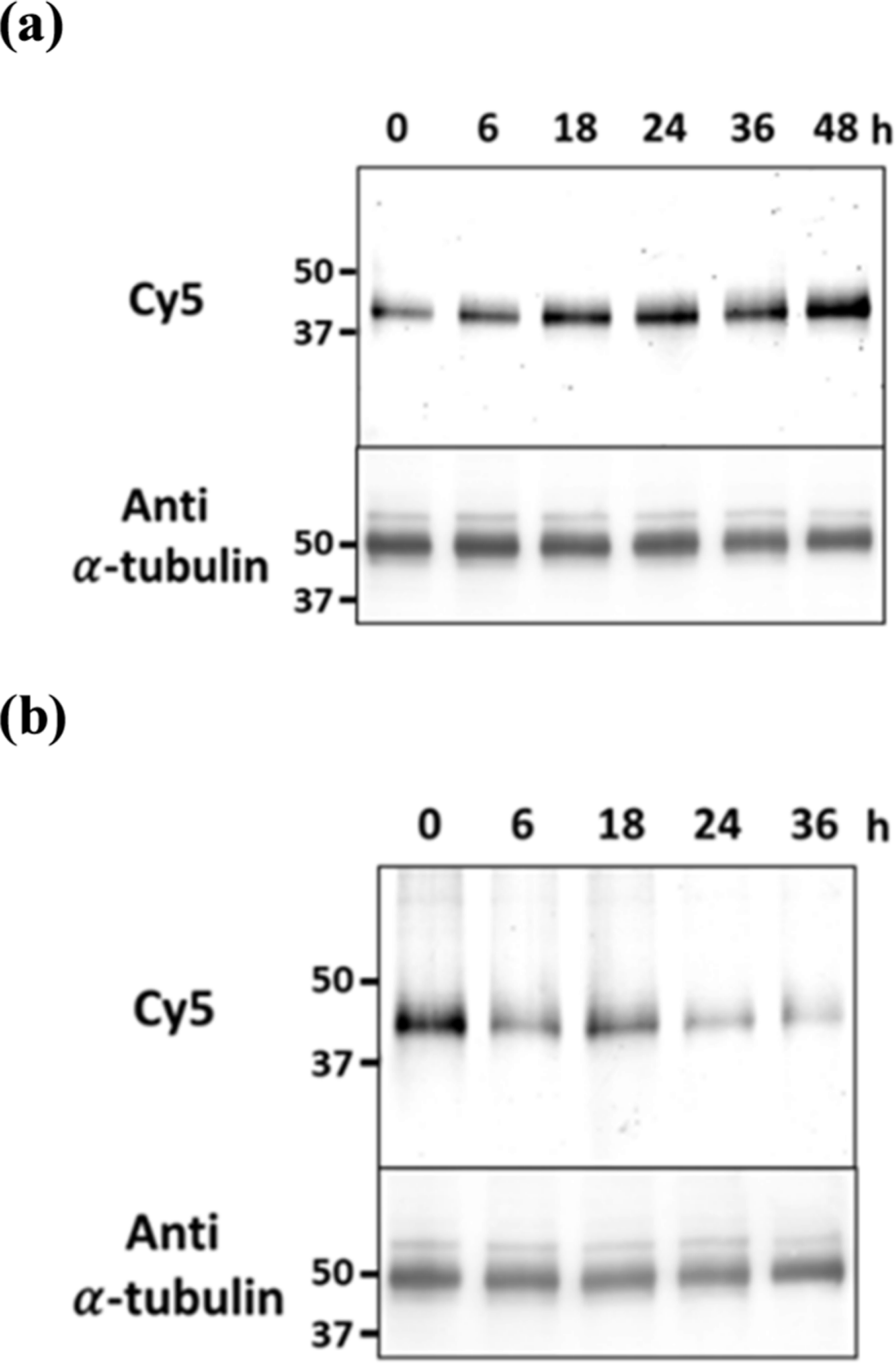
SDS-PAGE in-gel fluorescence analysis of **1**-labeled A549 cell lysates after incubation of the cells with (a) 200 μM CoCl_2_ or (b) 10 μM PMA in DMEM media at 37 °C for different time intervals.

### Detection of Endogenous Peroxynitrite in the Culture Media of RAW264.7 Macrophages

ONOO^–^ is an important reactive oxygen species (ROS) which can trigger a wide variety of biological processes, including immunodefense, signal transduction, and apoptosis after its secretion into the extracellular medium.^50,51^ Depending on the cellular environments and secretion levels, ONOO^–^ can either play a contributing role in nitrosylation signaling and microorganism defense or cause nitrosative stress to cells, resulting in lipid peroxidation, DNA strand breaks, and cell membrane damage.^52,53^ Currently, ONOO^–^ is commonly analyzed using fluorescent turn-on probes, which can react with ONOO^–^ to generate fluorescence signals.^54^

It has been reported that murine macrophage RAW264.7 cells can constantly release ONOO^–^ into culture medium.^55,56^ Therefore, RAW264.7 cells were used to demonstrate that probe **1** can be activated by endogenous ONOO^–^ for the subsequent labeling of hCA proteins. In this paper, RAW264.7 cells were treated with **1** and hCAII in the culture medium at 37 °C for 6 h. The medium was collected and analyzed by using SDS-PAGE to assess the secreted ONOO^–^ level. A strong fluorescent band was observed on the SDS-PAGE gel (Figures 5a and S20). The fluorescent bands became weaker when 1 mM ONOO^–^ quenchers, boronic acid, and uric acid were present in the media. We have shown in the test tubes that boronic acid and uric acid can react efficiently with ONOO^–^ to inhibit the oxidation of probe **1** by ONOO^–^ (Figure S21). To further investigate the kinetics of ONOO^–^ secretion, **1** and hCAII were incubated with RAW264.7 cells for different time intervals. The results showed that the amount of ONOO^–^ in the culture media increased gradually and peaked after 8 h (Figures 5b and S22). By coincubating peroxynitrite-releasing RAW264.7 cells with hCAXII-expressing A549 cells, the labeling of endogenous hCAXII with probe **1** can be triggered by the peroxynitrite released from RAW264.7 cells (Figure 5c). In the absence of RAW264.7 cells, probe **1** was unable to label A549 cells, resulting in very weak fluorescence signals observed within A549 cells (row 1). Conversely, the peroxynitrite secreted from RAW264.7 cells activates probe **1**, leading to the labeling of transmembrane hCAXII on A549 cells (row 3). The **1**-labeled transmembrane hCA is subsequently internalized through endocytosis, accumulating inside the cells due to the extended incubation time. This experiment demonstrates that this conditional affinity probe can be used for the detection of both endogenous ONOO^–^ and endogenous hCAs.

**Figure 5:**
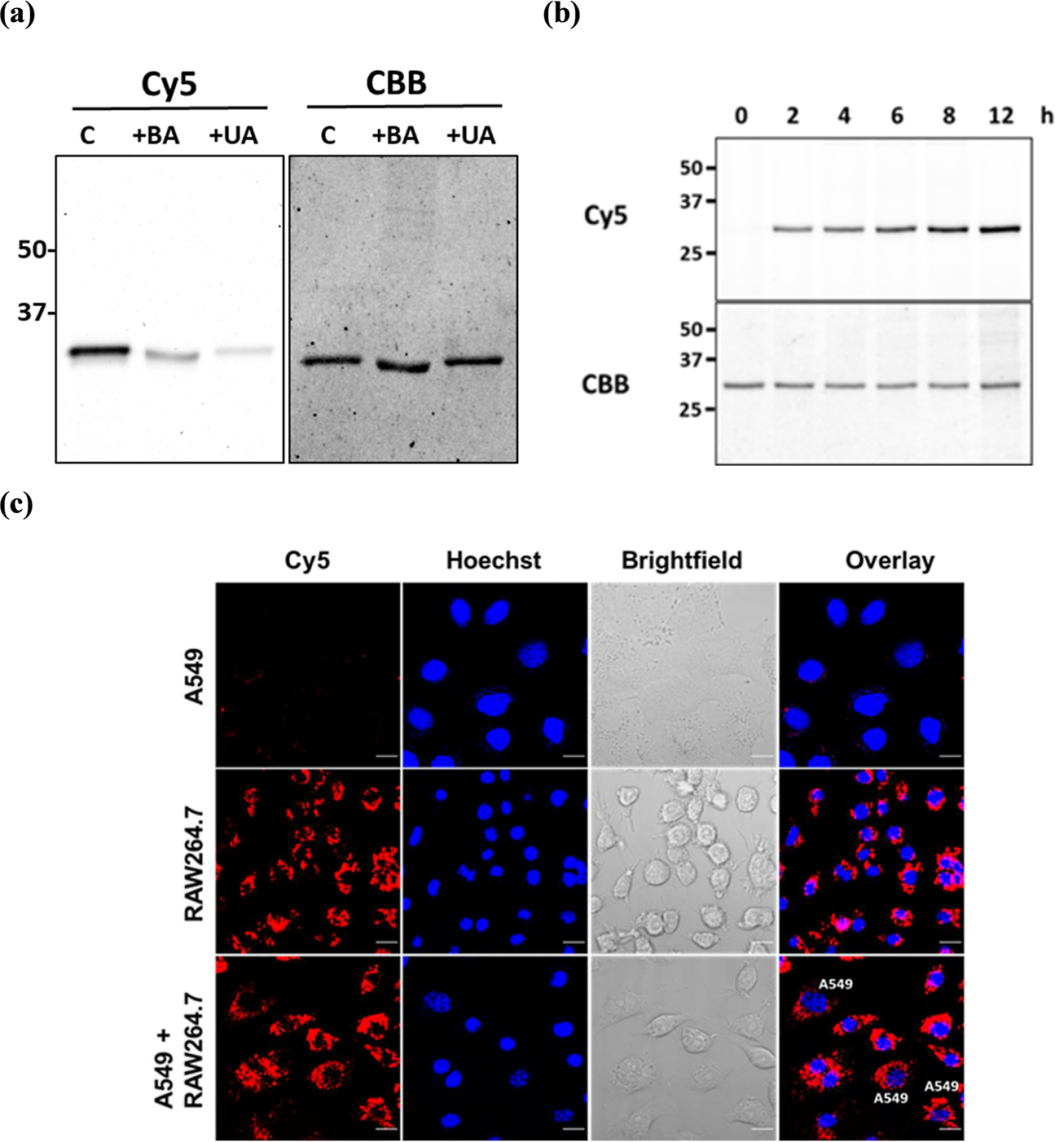
Detection of endogenous peroxynitrite secreted from RAW264.7 macrophages. (a) SDS-PAGE in-gel fluorescence analysis of **1**-labeled hCAII after incubation of probe **1** (1 μM) and hCAII (2 μM) with RAW264.7 macrophage cells in the absence or presence of uric acid (1 mM) and boronic acid (1 mM) at 37 °C for 6 h. C = condition without uric acid (UA) and boronic acid (BA). (b) Time course of peroxynitrite secretion from RAW264.7 macrophage cells. (c) Fluorescent images of A549 and RAW264.7 cells labeled with probe **1** after coincubation at 37 °C in DMEM media for 4 h. Cy5 and Hoechst 33342 are shown in red and blue, respectively. Scale bar: 20 μm.

### In Vivo Labeling and Imaging of Tumor-Bearing Mice Expressing Carbonic Anhydrase

Currently, almost all the existing in vivo protein labeling methods require a genetic tag (e.g., SNAP-tag or Halo-tag) to facilitate protein labeling.^57,58^ Previously, two protein labeling probes based on tosyl and difluorophenyl ester groups were employed for in vivo protein modification. However, the tosyl probe was used to label the abundant carbonic anhydrase I (CAI) of red blood cells, whereas the difluorophenyl ester probe might be sensitive to esterase, which is present in abundance in blood.^59,60^ To this end, we demonstrated that tumor cells expressing the hCAXII protein in living mice can be chemically modified by **1** and detected by using in vivo fluorescence imaging.

To demonstrate that the activation of **1** can achieve target protein labeling in vivo, tumor-bearing mice were used as animal xenograft models, which were prepared by subcutaneous inoculation of A549 cells. When the tumor lumps on the left leg grew to a suitable size, a premixed solution of 50 μM **1** with or without 500 μM SIN-1 was injected intratumorally and imaged using an IVIS imaging system. As shown in Figure 6a, bright fluorescence signals were detected from both groups of mice instantaneously after the injection. Subsequently, the fluorescence intensities decrease over time in both mice due to the rapid removal of the nonspecific accumulated probe by blood circulation. Whereas nonactivated **1** cannot be retained in the mice after 80 min of blood circulation, the mice that were injected with the activated **1** by SIN-1 showed prolonged and strong fluorescence at the tumor. Due to the formation of an irreversible covalent bond, we can still observe bright fluorescence at the tumor after 6 days of injection (Figure 6b). It is important to mention that premixing the control probe **2** with SIN-1 did not result in an accumulation of the probe at the tumor, implying that specific labeling of the target protein occurs in vivo. To understand the biodistribution of **1** in vivo, a 200 μL premixed solution of probe **1** and SIN-1 was intraperitoneally injected into the A549 tumor-bearing mouse, which was scarified after 2 h of injection (Figure S23). The ex vivo images showed that strong fluorescent signals can be observed in the tumor, despite some accumulation of the probe in the liver, stomach, and lung (Figure S24). As various carbonic anhydrase (CA) isozymes can be found in gastrointestinal mucosa, lung, red blood cells, and liver, it is reasonable to observe labeling of probe **1** in these organs.^61–64^ To confirm selective in vivo labeling of hCA, we performed SDS-PAGE in-gel fluorescence analysis on homogenized tumor tissue from mice treated with probe **1** and SIN-1. A distinct fluorescence band corresponding to endogenous hCAXII in A549 cells (MW ≈44 kDa) was observed (Figure S25). These results imply that the peroxynitrite-activated fluorescent chemical probe can be a useful strategy for long-term imaging and tracking of tumors in vivo.

**Figure 6:**
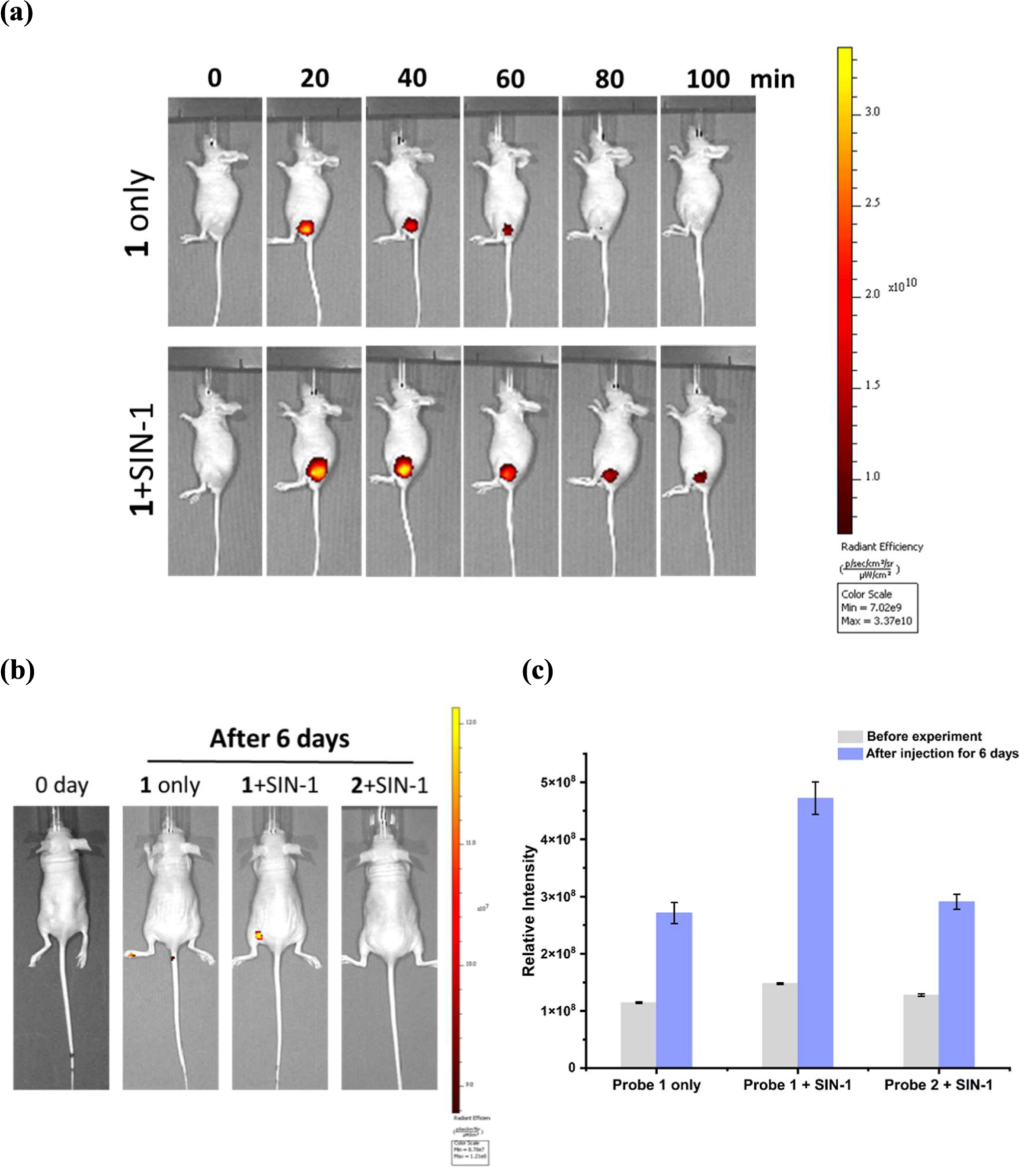
In vivo fluorescence images of A549 tumor-bearing mouse after intratumoral injection of (a) **1** only (50 μM, 100 μL) or (b) a 100 μL mixture of **1** (50 μM) and SIN-**1** (500 μM) into tumor at different times. (c) Fluorescence images of A549 tumor-bearing mouse after 6 days of intratumoral injection of (i) 50 μM **1** only, (ii) a 100 μL mixture of 50 μM **1** and 500 μM SIN-1, or (iii) a 100 μL mixture of 50 μM **2** (control) with 500 μM SIN-1. (c) Radiant efficiency of tumor region from fluorescence images in (b). λ_ex_ = 640 nm, λ_em_ = 680 nm.

## Conclusions

To close, we have presented the design, synthesis, and biological applications of a conditionally responsive chemical probe strategy for the analysis of peroxynitrite and hCA proteins. Unlike other condition-responsive probes, which use a latent quinone methide source for proximal covalent trapping upon activation, peroxynitrite oxidation of acyl hydrazide results in the formation of a reactive acyl diazo group, which can be readily displaced by amine to form a stable amide bond. As compared with the formation of quinone methide intermediate, our hydrazide probe can be easily synthesized and is more resistant to self-hydrolysis in aqueous solutions. By using gel electrophoresis, fluorescent live cell imaging, and a mice xenograft model, we demonstrated that this peroxynitrite-activated protein labeling probe can specifically and rapidly label hCAs in vitro and in vivo. Selectivity and sensitivity tests revealed that this probe is highly specific for peroxynitrite and hCAs, with the detection limit of 0.32 μM and 1.6 nM, respectively. After activation by the peroxynitrite precursor SIN-1, probe **1** can be employed to analyze upregulation and downregulation expression of hCAXII after hypoxia-induced and ectodomain shedding stimulation of A549 cells. In addition to protein labeling, this peroxynitrite-responsive protein labeling probe can also be employed to sense the peroxynitrite secreted from the macrophages. This conditionally responsive probe **1** remains in a dormant state until oxidation of the hydrazide group by peroxynitrite triggers a subsequent protein labeling reaction with the target protein. Therefore, the probe can be used for long-term imaging and tracking target proteins in vivo. We anticipate that such a conditionally responsive reagent will become an invaluable tool in providing important information on the target proteins in complex biological environments.

## Supplementary Material



## Notes

Probe synthesis, protein expression, fluorescent imaging, quantitative analysis, and NMR and HRMS spectra (PDF)
