# Supplementary material for: Peroxynitrite-Activated Conditional Chemical Probe for the Affinity-Based Protein Modification
Source: Anal Chem. 2025 Jul 10;97(28):14912–20. doi: 10.1021/acs.analchem.4c05580 (PMC12291043; doi:10.1021/acs.analchem.4c05580)
Supplement: Supplementary file 1 [file ac4c05580_si_001.pdf]

Supporting Information

Peroxynitrite-Activated Conditional Chemical Probe for the Affinity-based Protein Modification

Chien-Chi Wu,[†,#] Szu-Hsien Lee,[†,#] Ting-Ju Huang,[†,#] Jing-Cyun Lin,[†] Yueh-Hsun Lu,[§] Shu-Pao Wu[§,*] and Kui-Thong Tan[†,‡,*]

[†] Department of Chemistry, National Tsing Hua University, 101 Section 2, Kuang-Fu Road, Hsinchu 300044, Taiwan

[‡] Department of Medicinal and Applied Chemistry, Kaohsiung Medical University, Kaohsiung 80708, Taiwan

[§] Department of Applied Chemistry, National Yang Ming Chiao Tung University, Hsinchu 30010, Taiwan

[#] C.-C.W., S.-H. L., and T.-J. H. and contributed equally to this work

[*] Corresponding Author: kttan@mx.nthu.edu.tw and spwu@nycu.edu.tw

Table of Content

Materials and Instruments……………………………………………………………………S5

Experimental Section……………………………………………………………………S6

Figure S1……………………………………………………………………...S8

Figure S2…...…………………………………………………………………..S9

Figure S3…...…………………………………………………………………S10

Figure S4…...…………………………………………………………………S11

Figure S5…...…………………………………………………………………S12

Figure S6…...…………………………………………………………………S13

Figure S7…...…………………………………………………………………S14

Figure S8…...…………………………………………………………………S15

Figure S9…...…………………………………………………………………S16

Figure S10…...………………………………………………………………S17

Figure S11…...………………………………………………………………S18

Figure S12…...………………………………………………………………S19

Figure S13…...………………………………………………………………S20

Figure S14…...………………………………………………………………S21

Figure S15…...………………………………………………………………S22

Figure S16…...………………………………………………………………S23

Figure S17…...………………………………………………………………S24

Figure S18…...………………………………………………………………S25

Figure S19…...………………………………………………………………S26

Figure S20…...………………………………………………………………S27

Figure S21…...………………………………………………………………S28

Figure S22…...………………………………………………………………S29

Figure S23……S30

Figure S24……S31

Figure S25……S32

Scheme S1……S33

Scheme S2……S34

Synthesis of Compound **3**……S35

Synthesis of Compound **4**……S35

Synthesis of Compound **5**……S36

Synthesis of Compound **6**……S36

Synthesis of Compound **7**……S37

Synthesis of Compound **8**……S37

Synthesis of Compound **9**……S38

Synthesis of Compound **10**……S38

Synthesis of Compound **11**……S39

Synthesis of **Probe 1**……S40

Synthesis of Compound **14**……S41

Synthesis of Compound **15**……S41

Synthesis of **Probe 2**……S42

Synthesis of **Probe 3**……S43

Figure 26……S44

Figure 27……S45

Figure 28……S46

Figure 29……S47

Figure 30……S48

Figure 31……S49

Figure 32......S50

Figure 33......S51

Figure 34......S52

Figure 35......S53

Figure 36......S54

Figure 37......S55

Figure 38......S56

Figure 39......S57

Figure 40......S58

Figure 41......S59

Figure 42......S60

Figure 43......S61

Figure 44......S62

Figure 45......S63

Figure 46......S64

Figure 47......S65

Figure 48......S66

Figure 49......S67

Figure 50......S68

Figure 51......S69

Figure 52......S70

Figure 53......S71

Figure 54......S72

Figure 55......S73

Materials and Instruments

Chemicals and reagents were purchased from Sigma-Aldrich and TCI and used without further purification. All solvents (DMSO, DMF, acetonitrile, dichloromethane, hexane, ethyl acetate, and methanol) were purchased from Sigma-Aldrich and TCI and used without further treatment or distillation. Thin layer chromatography (TLC) was performed on TLC-aluminum sheets (Silica gel 60 F254, Merck). Flash column chromatography was performed with silica gel (230-400 mesh, Merck). HPLC analysis was performed with an analytical column (XBridge BEH C18 Column, 130Å, 5 mm, 4.6 mm x 250 mm). Products were purified either by HPLC semi-preparative column (Cosmosil 20ID X 150 mm, 5C18-AR-300, Nacalai Tesque) or by Lichroprep RP-18 (40-63 µm) packed column.

^{1}H, and ^{13}C nuclear magnetic resonance (NMR) spectra were recorded either on Varian Unityinova-500, Varian MR-400 or Bruker-400 with ^{1}H chemical shifts (δ) reported in ppm relative to the solvent residual signals of d-chloroform (7.24 ppm), d-methanol (3.31 ppm), d-DMSO (2.49 ppm), D_2O (4.79 ppm). ^{13}C chemical shifts (δ) were reported in ppm relative to the solvent residual signals of d-chloroform (77.0 ppm), d-methanol (49.0 ppm) and d-DMSO (39.5 ppm). High-resolution mass spectra (HRMS) with electrospray ionization (ESI) were measured on JOEL JMS-T100LP 4G. In-gel fluorescence images were recorded by using ChemiDoc Touch Imaging system (Bio-rad Inc, CA, USA) and analyzed by using Image lab software (Bio-rad Inc, CA, USA). BCA (BCA Protein Assay Kit from Thermo Scientific Inc, USA) and MTT assays were analyzed by using TECAN Infinite M200 PRO (Tecan Group Ltd, ZH, Switzerland). The fluorescent images were taken by using Laser Scanning Confocal Microscope (LSM 700, Zeiss, Germany). For Cy5 dye, the images were taken by using 639 nm excitation laser and LP640 emission filter. For Hoechst, 405 nm excitation laser and SP490 emission filter were used. The fluorescence intensities were quantified by Image J software.

Experimental Section

Preparation of various ROS and RNS solutions for selectivity studies

1. Peroxynitrite ($ONOO^-$) was generated from SIN-1 (Sigma Aldrich). SIN-1 was dissolved in ddH_2O to prepare a 10 mM stock solution.
2. Nitric oxide (NO) donor was generated from DEA NONOate (diethylamine nonoate, abcam). DEA NONOate was dissolved in ddH_2O to prepare a 10 mM stock solution.
3. H_2O_2 in ddH_2O was diluted from commercial H_2O_2 solution (10 M, SHOWA).
4. HOCl in ddH_2O was diluted from commercial NaOCl solution (5% available chlorine, J.T. Baker).
5. Nitrate (NO_3^-) and nitrite (NO_2^-) were prepared individually from sodium nitrate and sodium nitrite. They were dissolved in ddH_2O to make up a 1 mM stock solution.
6. Superoxide (O_2^-) solution was prepared by dissolving KO_2 in DMSO (10 mM).

hCAII protein expression and purification. Plasmid pET51b-hCAII with C-terminal His-tag was transformed to *E. coli* strain BL21. The bacteria were cultured at 37 °C in LB broth medium to OD_{600nm} of approximately 1.0. Protein expression was induced by adding 0.24 g/mL IPTG. The bacteria were grown for an additional 18 hours at 18 °C and harvested by centrifugation. The cells were lysed by sonication. Insoluble proteins and cell debris were removed by centrifugation. The recombinant hCAII protein was then purified by His-Tag Purification Resin (Roche, Basel, Switzerland) and eluted by imidazole elution buffer. The purified protein was concentrated and transferred to a PBS buffer using 10K Amicon Ultra centrifugal filters (Merck KGaA, Darmstadt, Germany) and stored at -20 °C. The concentration of the protein was determined using BCA assay. The purity of the protein was checked by SDS-PAGE and stained by Commassie brilliant blue dye.

Cytotoxicity Assay Using CCK-8 reagent. A549 cells were cultured in DMEM supplemented with 10% fetal bovine serum (FBS) and 1% penicillin-streptomycin. Cells were seeded at a density of 5,000 cells per well in a 96-well tissue culture-treated

plate (Falcon, Cat. No. 353072) and incubated for 24 hours at 37 °C in a humidified atmosphere containing 5% CO_2. After incubation, the cells were washed once with fresh DMEM. Subsequently, 1 µM of probe **1** and indicated additives were added to the wells and incubated at 37 °C for the specified durations. After treatment, cells were washed twice with DMEM. Then, 10 µL of CCK-8 solution was added to each well containing 100 µL of DMEM, and the plate was incubated for 2 hours at 37 °C. Absorbance was measured at 450 nm using a microplate reader (Tecan Infinite M200 Pro).

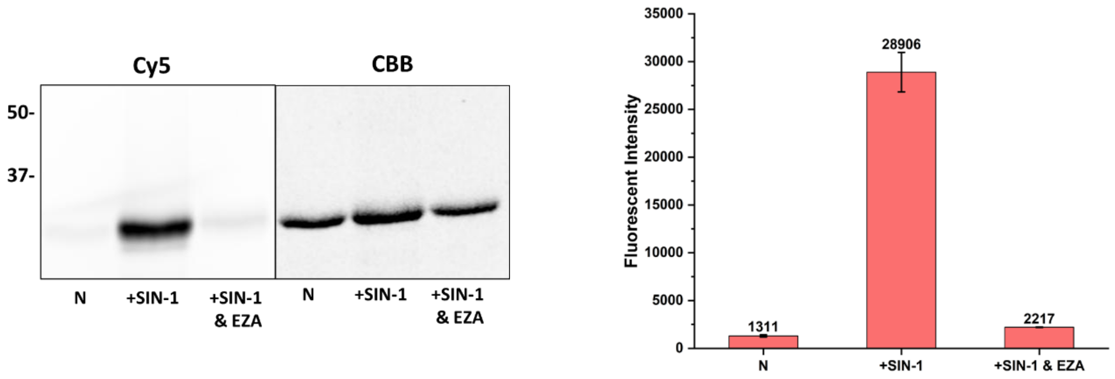

Figure S1. SDS-PAGE in-gel fluorescence image and quantitative analysis. The labeling reaction was conducted with 20 μM hCAII and 10 μM probe **1** in the absence or presence of 100 μM SIN-1 or 100 μM EZA at 37 °C in pH 7.4 PBS buffer for 30 minutes. N = without SIN-1.

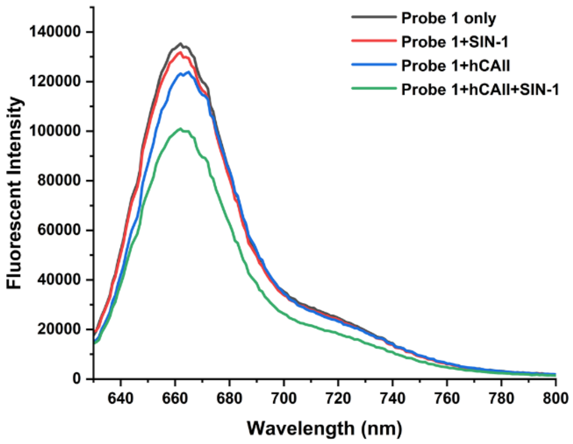

Figure S2. Fluorescence spectra analysis of 10 µM probe **1** in the absence or presence of 20 µM hCAII and 100 µM SIN-1 at 37 °C in PBS buffer for 30 minutes. The mixture was diluted 10-fold in PBS buffer and the fluorescence spectra were recorded using a TECAN Infinite M200Pro fluorescent spectrometer.

(a)

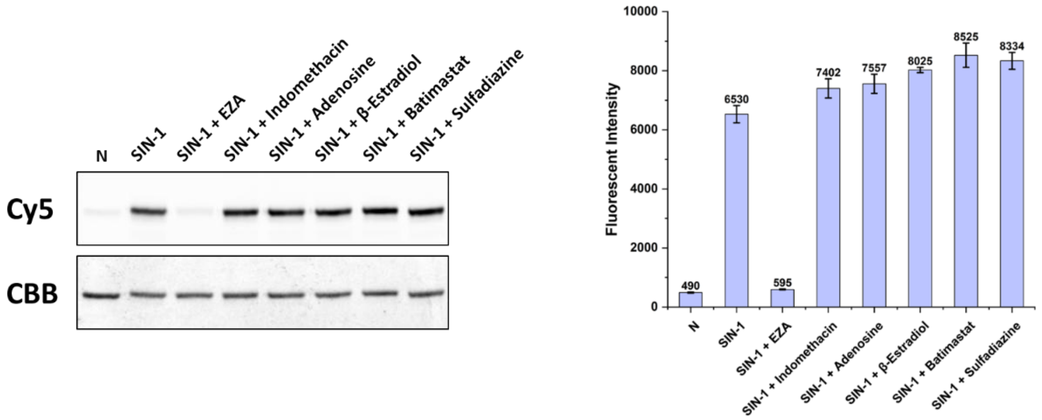

(b)

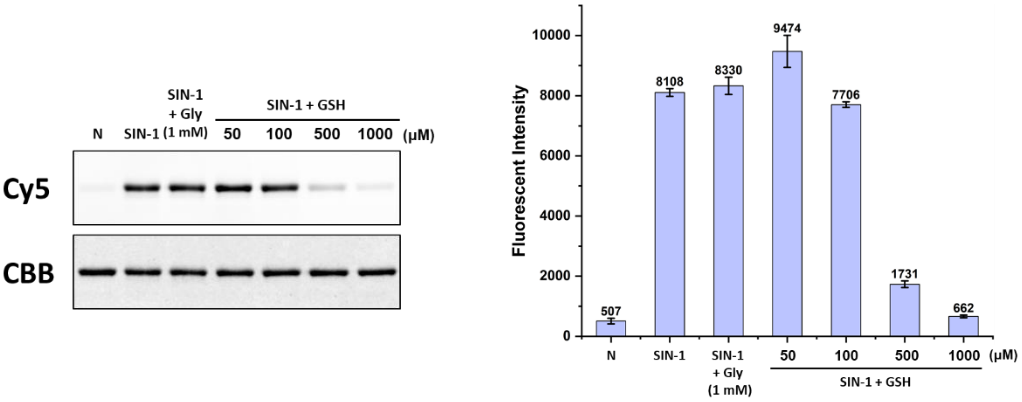

Figure S3. SDS-PAGE in-gel fluorescence analysis following the labeling of 20 μM hCA protein with 10 μM probe **1** and 100 μM SIN-1 (a) in the presence of various non-sulfonamide inhibitors (100 μM) and (b) 1 mM glycine and various concentrations of glutathione. The reaction was conducted at 37 °C in pH 7.4 PBS buffer for 30 minutes. N = without SIN-1.

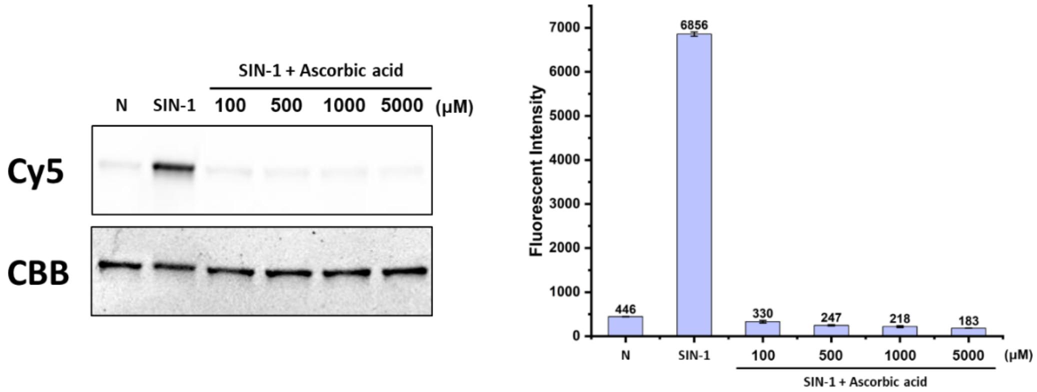

Figure S4. SDS-PAGE in-gel fluorescence analysis following the labeling of 20 μM hCA protein with 10 μM probe **1** and 100 μM SIN-1 in the presence or various concentrations of ascorbic acid. The reaction was conducted at 37 °C in pH 7.4 PBS buffer for 30 minutes. N = without SIN-1.

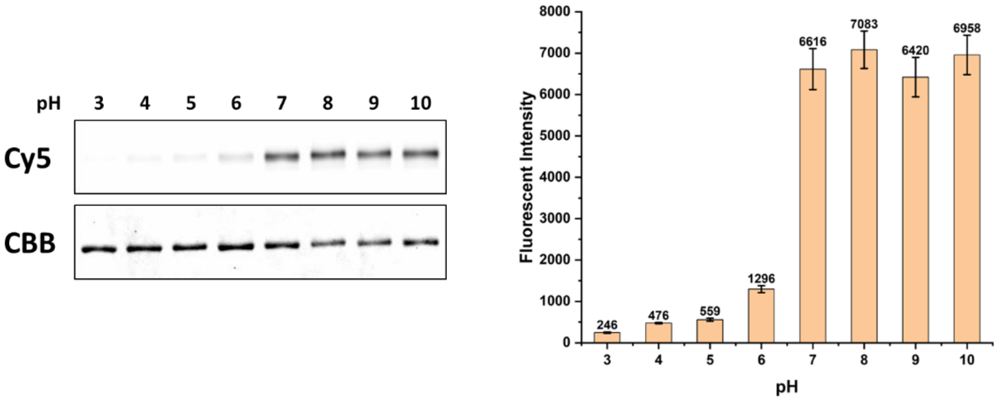

Figure S5. SDS-PAGE in-gel fluorescence analysis following the labeling of 20 μM hCA protein with 10 μM probe **1** and 100 μM SIN-1 at different pH levels. The reaction was conducted at 37 °C in PBS buffer for 30 minutes.

(a)

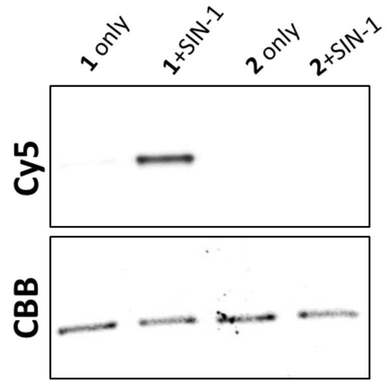

(b)

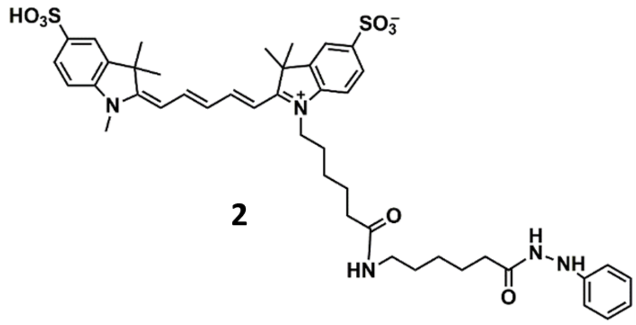

Figure S6. (a) SDS-PAGE in-gel fluorescent analysis of the hCAII (2 μM) labeling reaction with 1 μM of **1** and **2**, respectively, in the absence or presence of 100 μM SIN-1 for 30 minutes at 37 °C in pH 7.4 PBS buffer. (b) Chemical structure of control probe **2**.

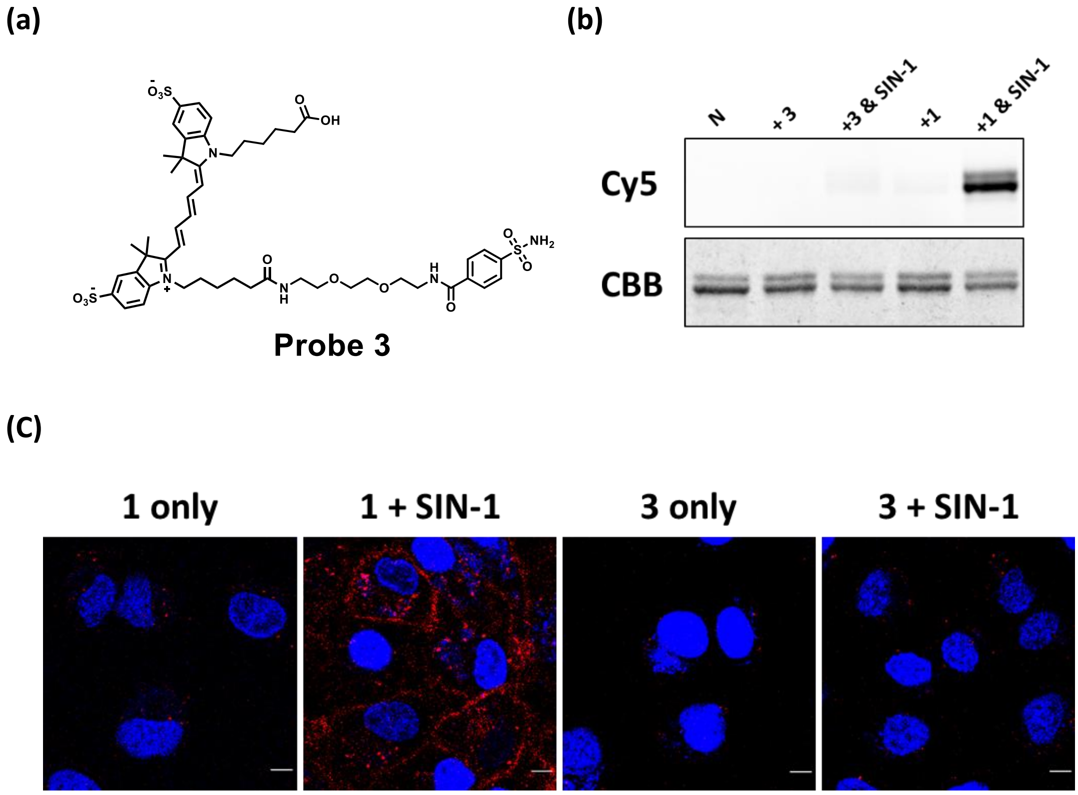

Figure S7. Comparison of probe **1** and control probe **3** for hCAII labeling. (a) Chemical structures of control probe **3**. (b) SDS-PAGE in-gel fluorescence analysis following the labeling of 20 μM hCA protein with 10 μM probe **1** or **3**, in the absence or presence of 100 μM SIN-1. N = without SIN-1. (c) Fluorescence images of live A549 cells incubated with probe **1** or **3**, with or without SIN-1 treatment.

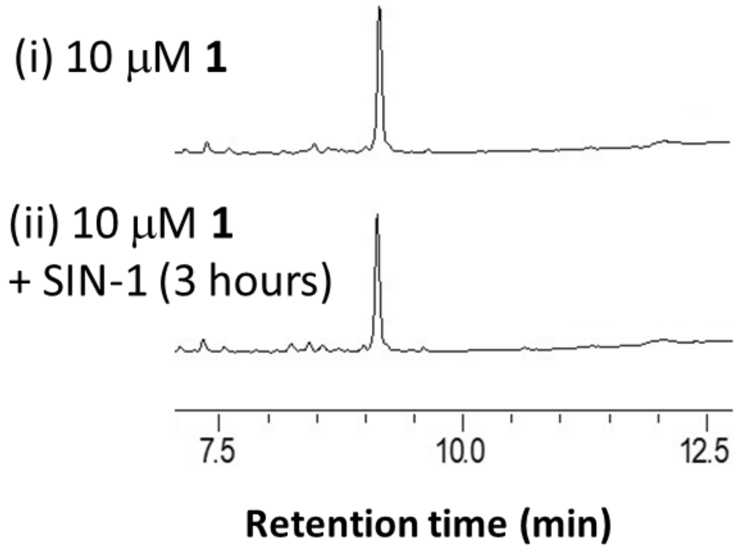

Figure S8. HPLC trace of (i) probe **1** and (ii) after incubation with 100 μM SIN-1 in PBS buffer at 37 °C for 3 hours.

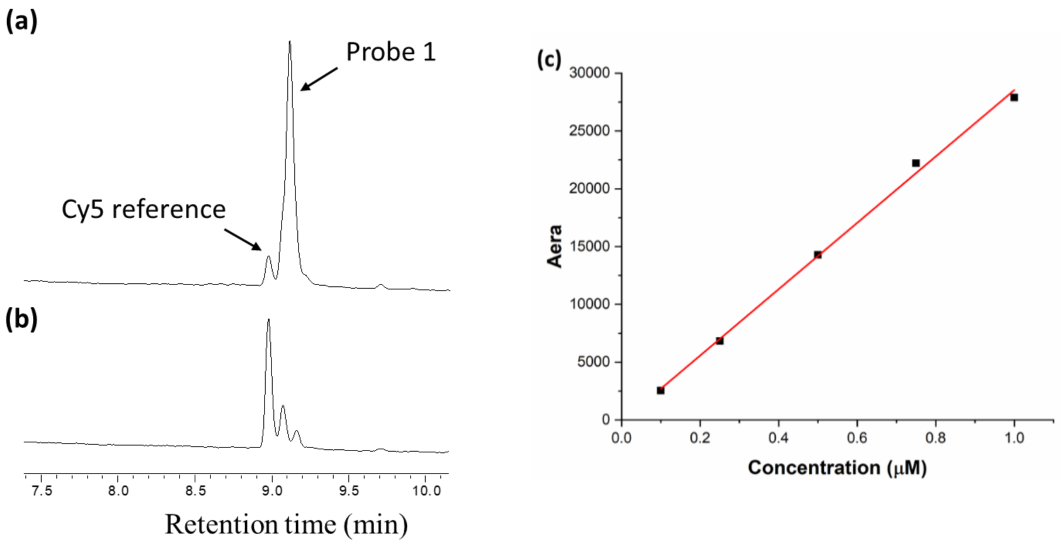

Figure S9. (a) HPLC trace of probe **1** and internal standard Cy5 dye. (b) HPLC trace of probe **1** and internal standard Cy5 dye after hCAII protein labeling in the presence of 100 μM SIN-1 in pH 7.4 PBS buffer at 37 °C for 30 minutes. (c) Titration curve of probe **1** determined by integrating the peak area of **1**.

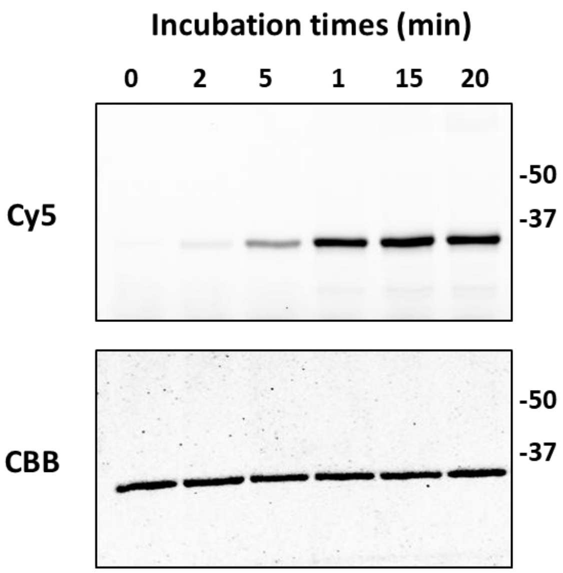

Figure S10. SDS-PAGE in-gel fluorescence images after incubation of hCAII (2 μM) with **1** (1 μM) and 100 μM SIN-1 at 37 °C in pH 7.4 PBS buffer for the indicated times.

(a)

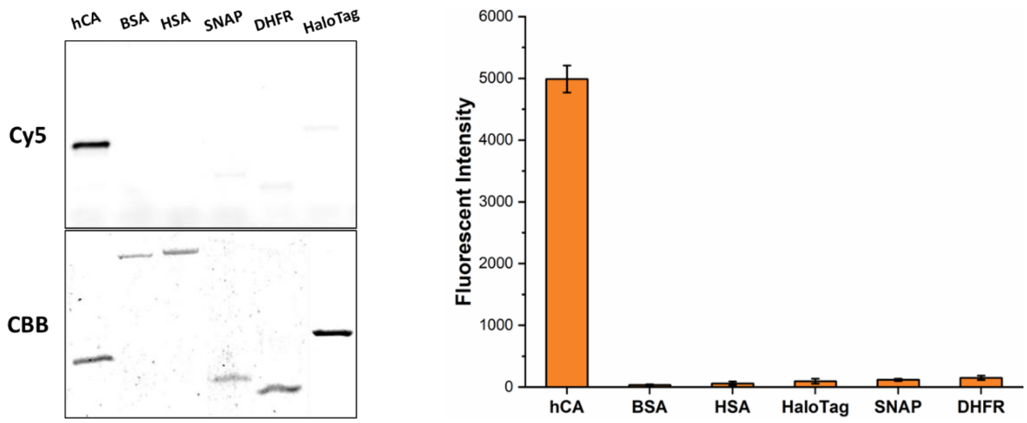

(b)

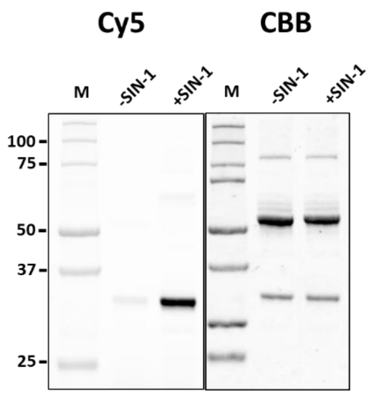

Figure S11. (a) SDS-PAGE in-gel fluorescence images and quantitative analysis of the fluorescent bands after labeling of different proteins (2 μM each for hCA, BSA, HSA, recombinant SNAP-tag, DHFR and Hal-tag proteins) with 1 μM **1** and 100 μM SIN-1. (b) SDS-PAGE in-gel fluorescence analysis following the labeling of hCA protein with probe **1** and SIN-1 in 10% fetal bovine serum (FBS). The labeling was performed in a pH 7.4 PBS buffer at 37 °C for 30 minutes.

(a)

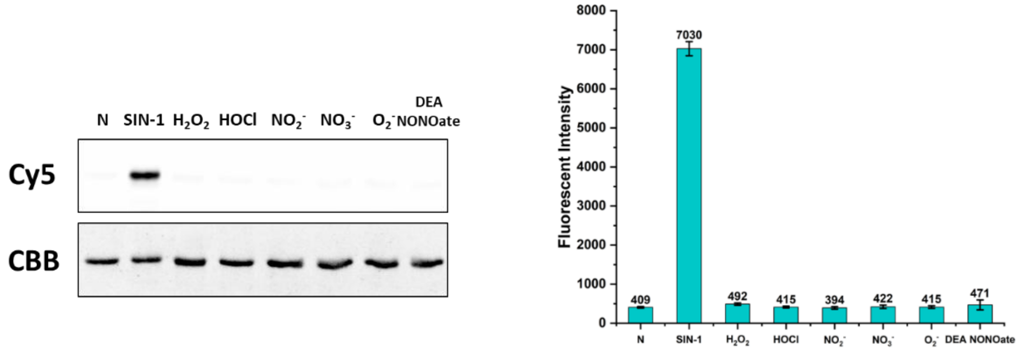

(b)

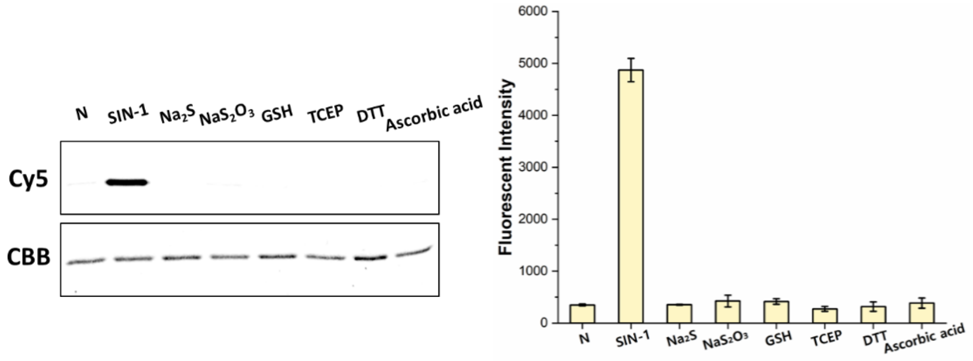

Figure S12. SDS-PAGE in-gel fluorescence images and quantitative analysis of the fluorescent bands after labeling of 20 μM hCA with 10 μM **1** and in the presence of (a) 100 μM SIN-1, H_2O_2, HOCl, NO_2^-, NO_3^-, O_2^-, and DEA NONOate (NO donor), or (b) 100 μM oxidants and reductants, including SIN-1, Na_2S, NaS_2O_3, GSH, TCEP, DTT and ascorbic acid. The labeling was performed in a pH 7.4 PBS buffer at 37 °C for 30 minutes. N = without SIN-1.

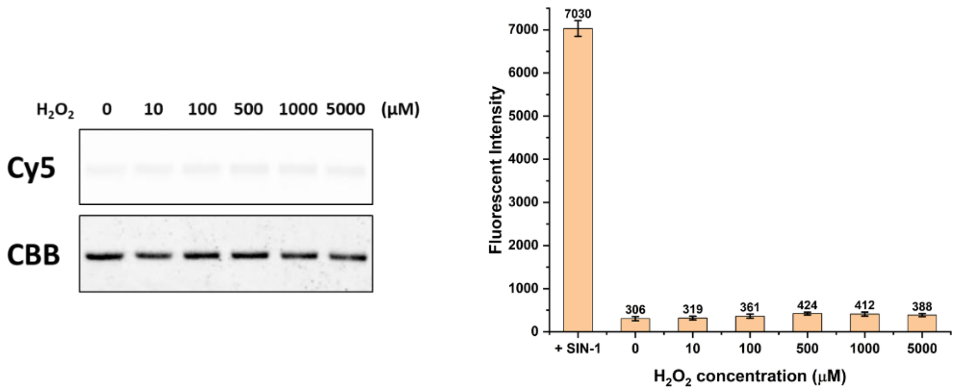

Figure S13. SDS-PAGE in-gel fluorescence analysis following the labeling of 20 μM hCA protein with 10 μM probe **1** in the presence of various concentrations of H_2O_2. The reaction was conducted at 37 °C in pH 7.4 PBS buffer for 30 minutes.

(a)

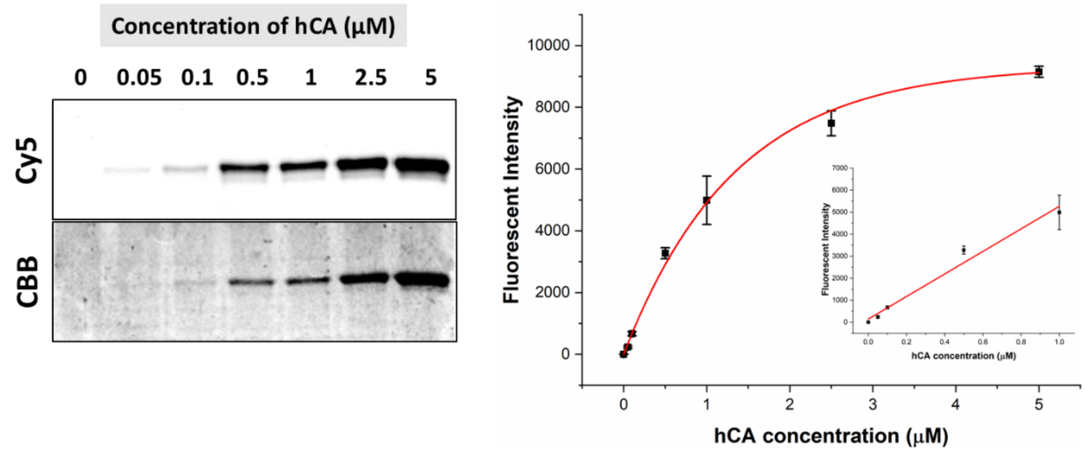

(b)

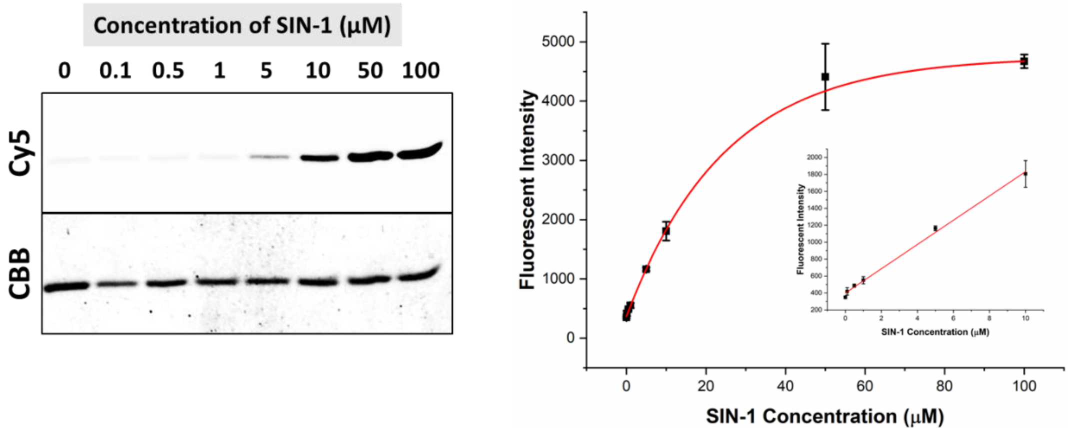

Figure S14. SDS-PAGE in-gel fluorescence images and quantitative analysis of the fluorescent bands for the determination of detection limit of (a) hCA protein and (b) $ONOO^{-}$ using probe **1**. For (a), 1 μM **1** was incubated with different concentrations of hCAII (0.05 μM – 5 μM) in pH 7.4 PBS buffer at 37 °C for 30 minutes in the presence of 100 μM SIN-1. For (b)

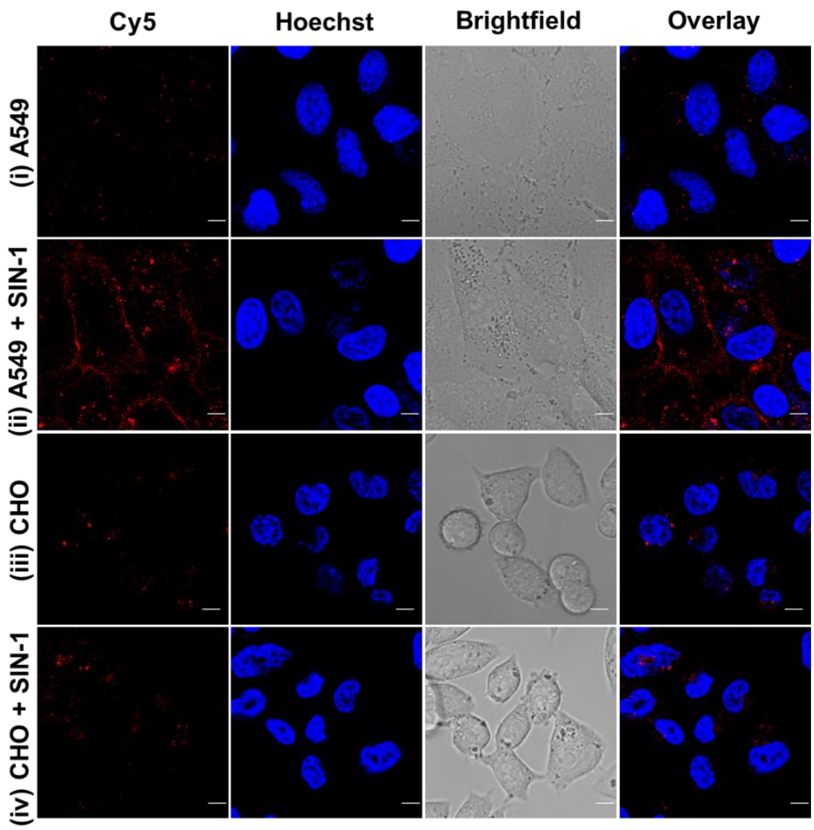

Figure S15. Fluorescent images of (i) and (ii) A549, (iii) and (iv) CHO cells after incubation with **1** μM probe **1** and 100 μM SIN-1 at 37 °C in DMEM media for 30 minutes. Cy5 and Hoechst 33342 are shown in red and blue, respectively. Scale bar: 20 μm.

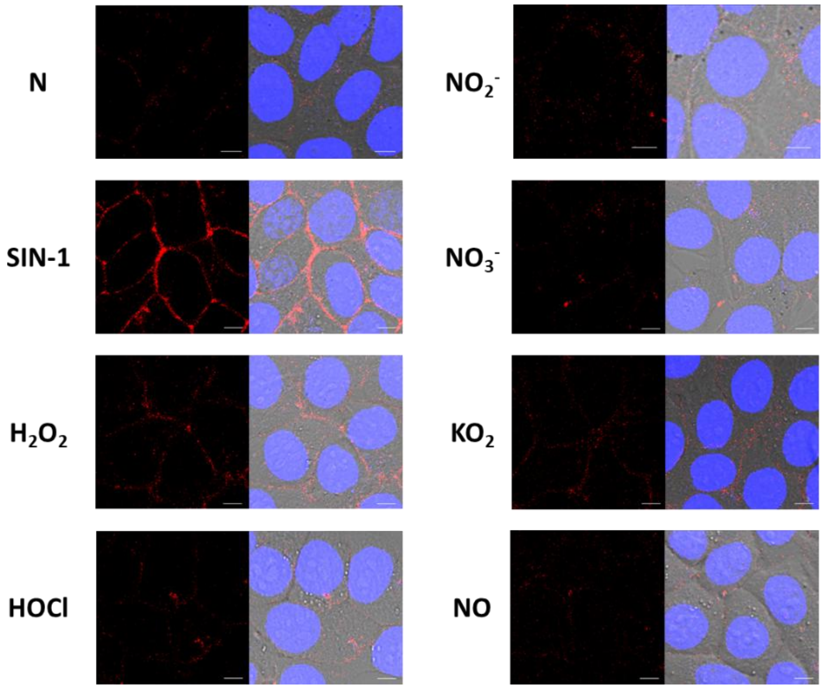

Figure S16. Live cell fluorescent imaging of **1**-labeled MCF7 cells in the absence or presence of 100 μM SIN-1, H2O2, HOCl, NO2-, NO3-, KO2 or NO donor. Cell surfaces stained with probe 1 are shown in red and the nuclei labeled with Hoechst 34580 are shown in blue. Scale bar: 20 μm.

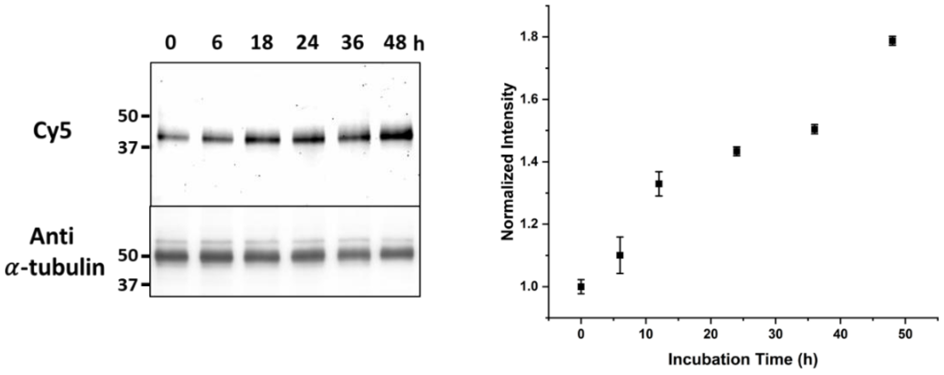

Figure S17. Labeling of transmembrane carbonic anhydrase after hypoxia-induced condition using $CoCl_2$. SDS-PAGE in-gel fluorescence and quantitative analysis of **1**-labeled A549 cell lysates after incubation of the cells with 200 μM $CoCl_2$ in DMEM media at 37 °C for different time intervals.

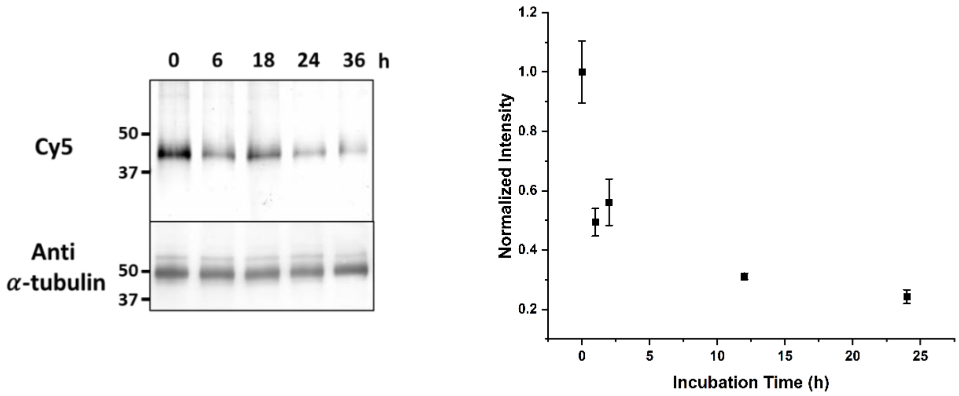

Figure S18. Labeling of transmembrane carbonic anhydrase after ectodomain shedding stimulation. SDS-PAGE in-gel fluorescence and quantitative analysis of **1**-labeled A549 cell lysates after incubation of the cells with 10 μM PMA in DMEM media at 37 °C for different time intervals.

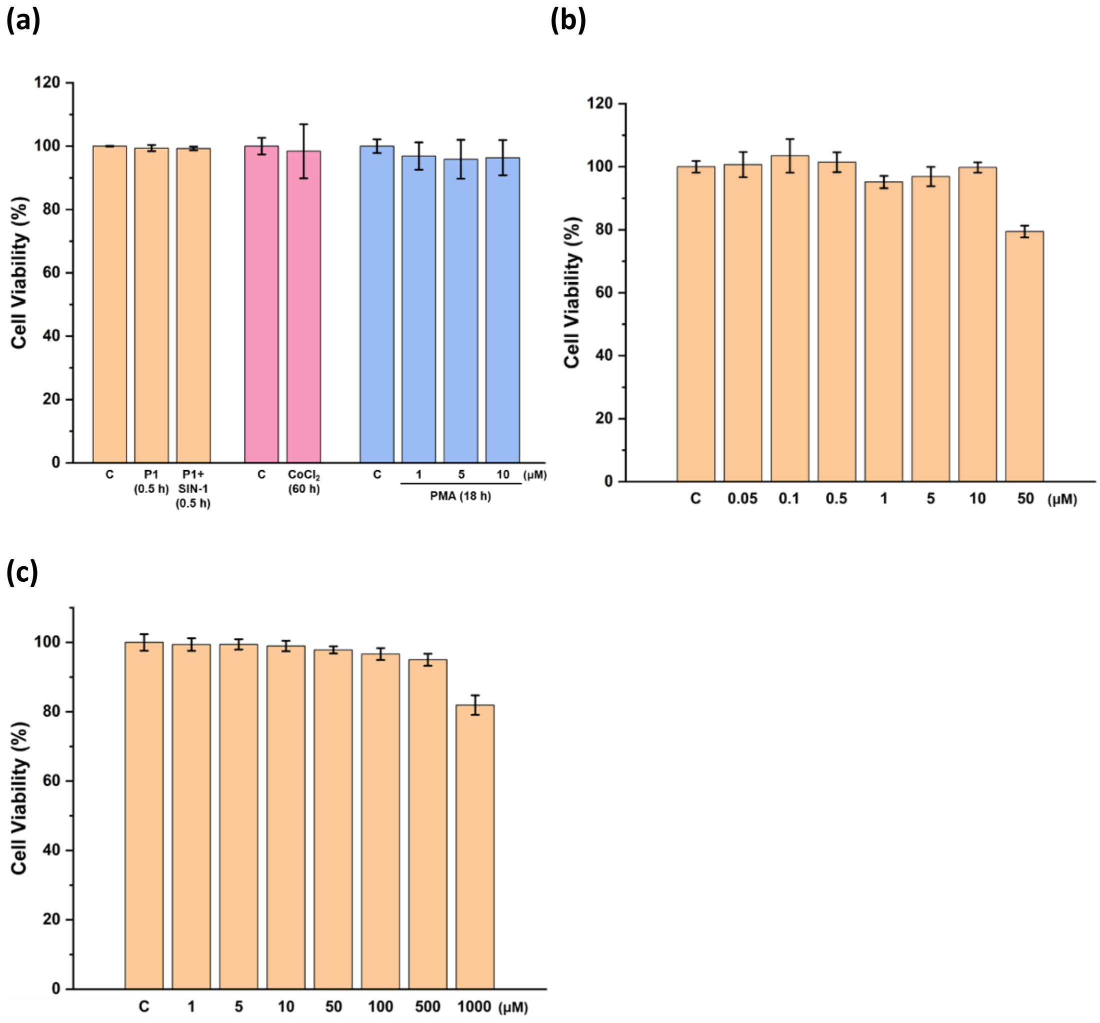

Figure S19. Determination of the cellular toxicity of (a) PMA and $CoCl_2$ and (b) probe **1** and (c) SIN-1 on A549 cells using the CCK-8 assay. The cells were incubated with the tested reagents for the specified durations at 37 °C in culture medium. C = control (without the tested reagents).

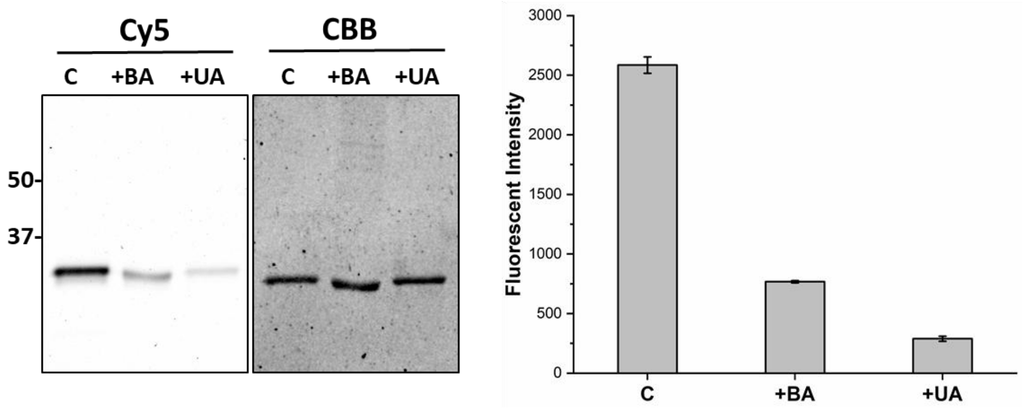

Figure S20. Detection of endogenous peroxynitrite in the culture media of RAW264.7 macrophages. SDS-PAGE in-gel fluorescence and quantitative analysis of **1**-labeled hCAII after incubation of probe **1** (1 μM) and hCAII (2 μM) with RAW264.7 macrophage cells in the absence or presence of 1 mM uric acid and boronic acid at 37 °C for 6 hours. C = condition without uric acid (UA) and boronic acid (BA).

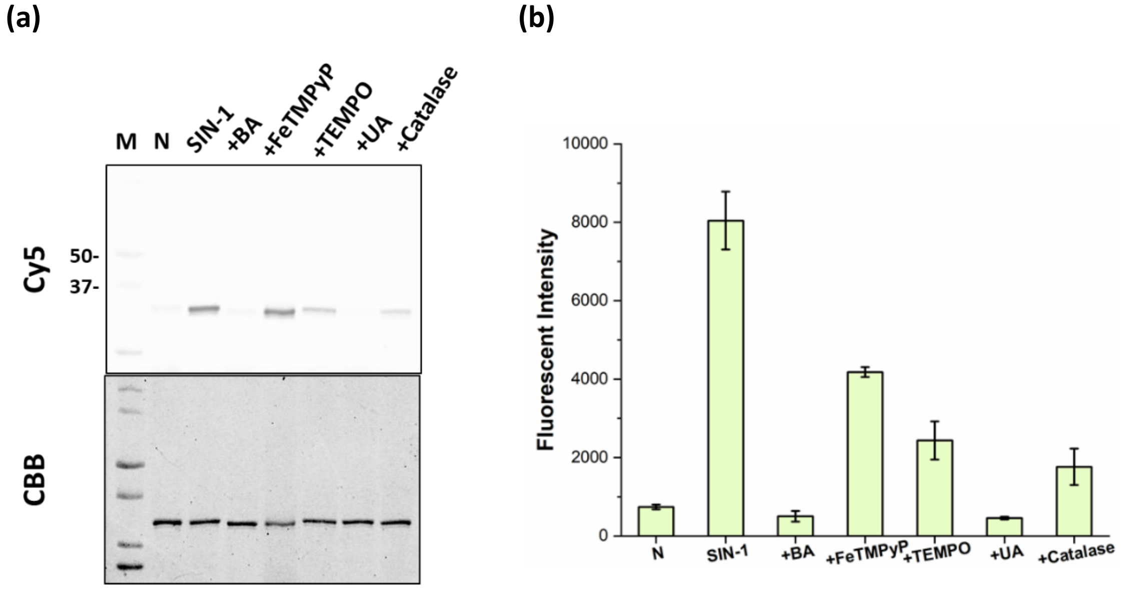

Figure S21. (a) SDS-PAGE in-gel fluorescence images and (b) quantitative analysis of the fluorescent bands after labeling of hCA with probe 1 and 100 μM SIN-1 or in the presence of 1 mM boronic acid (BA), 1 mM FeTMPyP, 1 mM TEMPO, 1 mM Uric acid (UA), 1 μg/mL catalase. The reaction mixture was incubated for 30 minutes at 37 °C in PBS buffer.

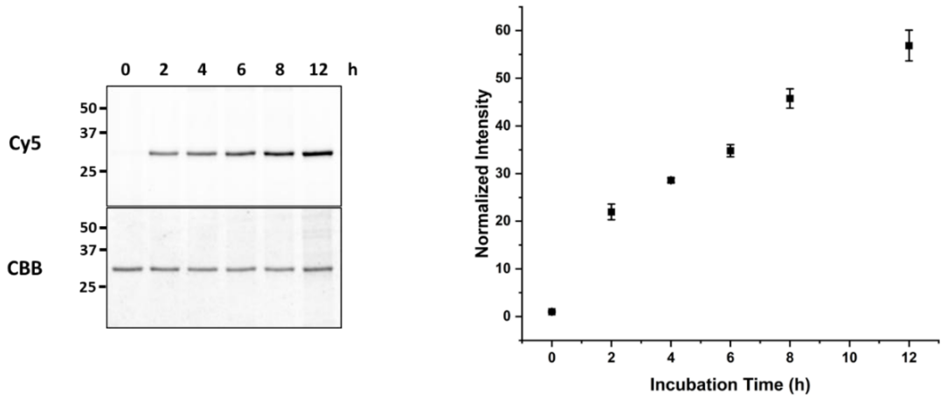

Figure S22. SDS-PAGE in-gel fluorescence images and quantitative analysis to determine the time course of peroxynitrite secretion from RAW264.7 macrophage cells.

(a)

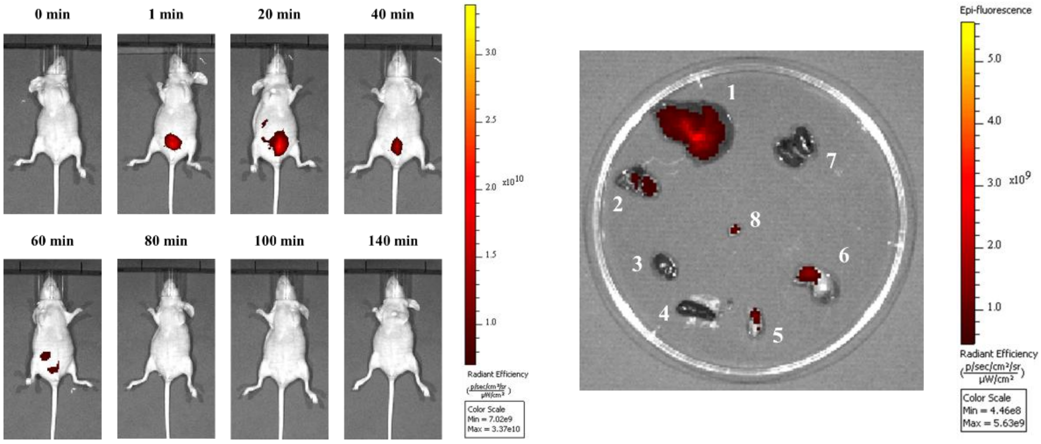

(b)

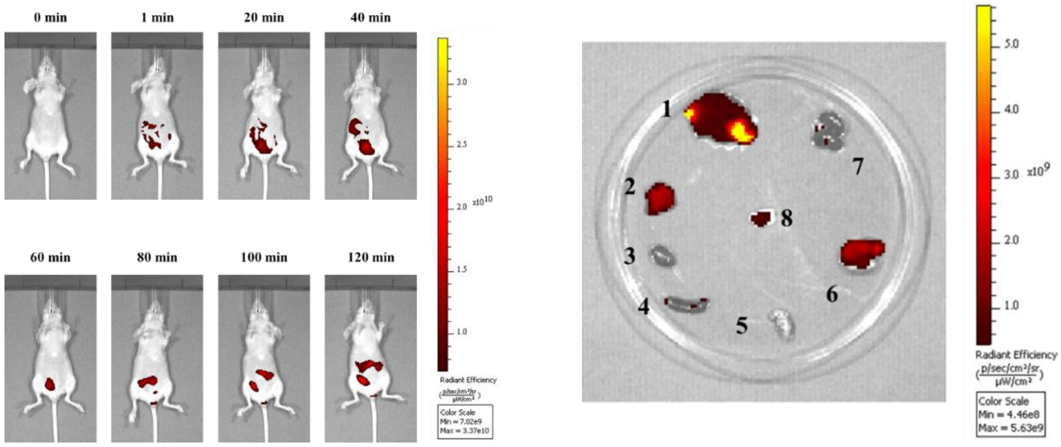

Figure S23. In vivo and ex vivo fluorescence images of A549 tumor-bearing mice at different times after intraperitoneal injection of a (a) 200 μL mixture of probe **1** (50 μM) only and (b) with SIN-1 (500 μM). Ex vivo fluorescence images of dissected organs from A549 tumor-bearing mouse after intraperitoneal injection of a 200 μL mixture of probe **1** (50 μM) and SIN-1 (500 μM) for 3h. 1. Liver, 2. Lung, 3. Heart, 4. Spleen, 5. Pancreas, 6. Stomach, 7. Kidney, 8. Tumor. Fluorescence: λex = 640 nm, λem = 680 nm.

(a) (b)

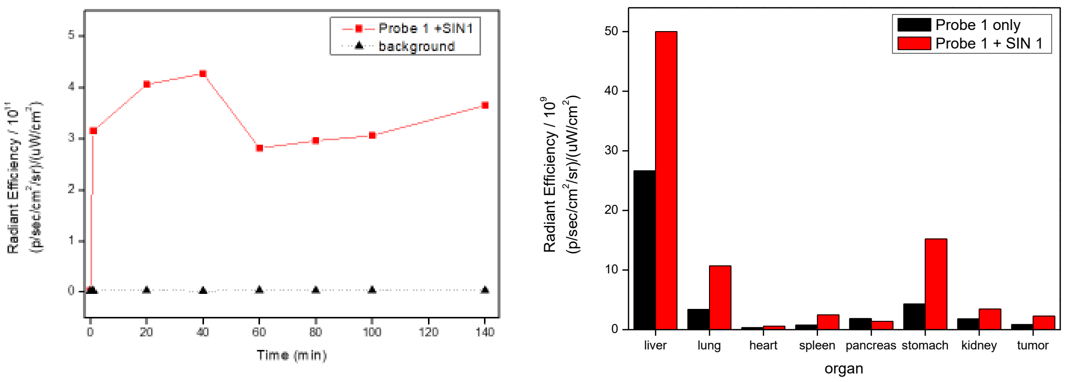

Figure S24. In vivo fluorescence analysis of tumor-bearing mice injected with probe **1** and SIN-1. (a) Comparison of fluorescence signals in the chest region and the intraperitoneal cavity of tumor-bearing mice following injection with probe **1** and SIN-1. (b) Quantification of fluorescence intensity across major organs of tumor-bearing mice after injection with probe **1**, with or without co-administration of SIN-1.

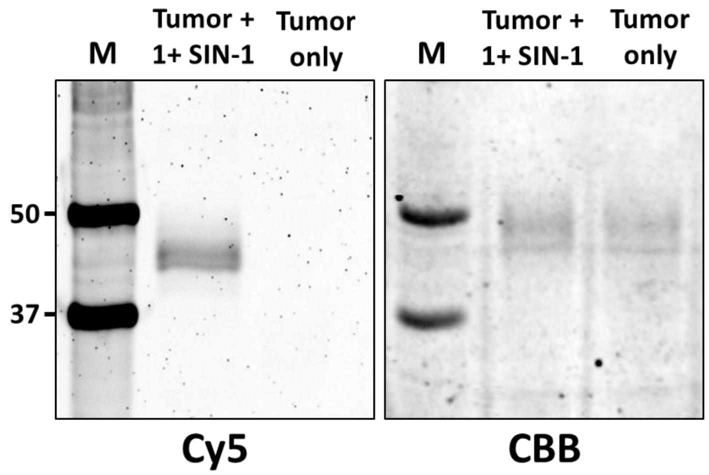

Figure S25. SDS-PAGE In-gel fluorescence analysis of tumor homogenates from mice injected with probe **1** and SIN-1.

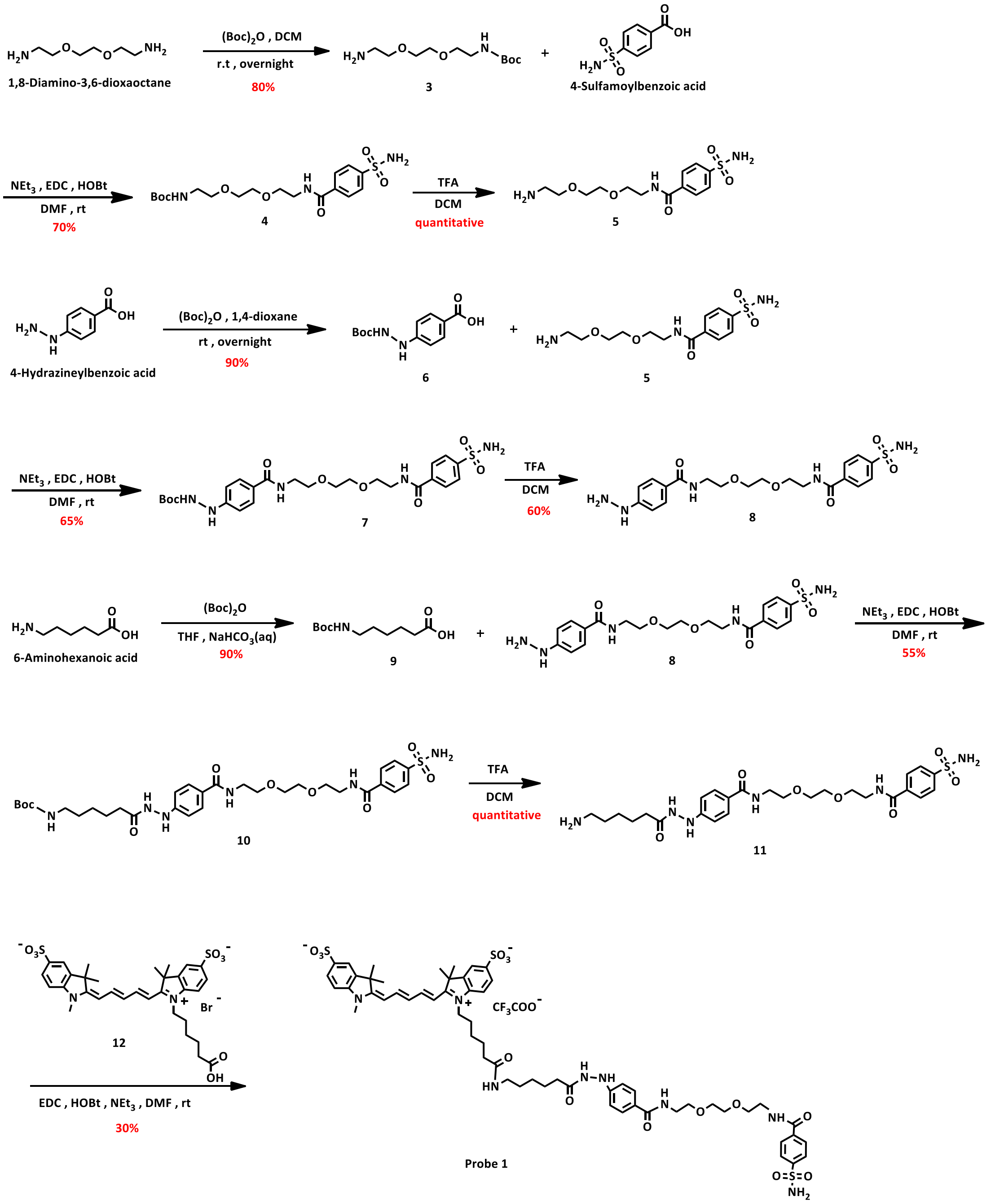

Scheme S1. Synthesis of probe **1**.

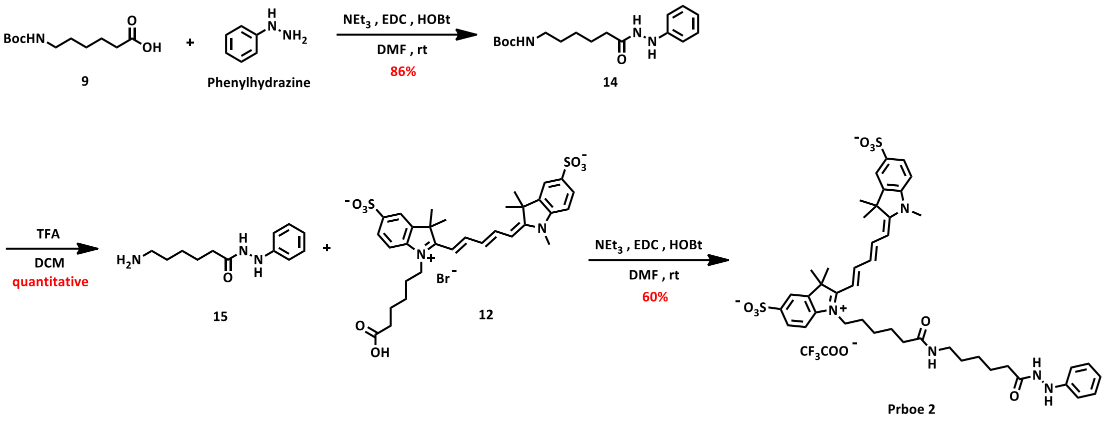

Scheme S2. Synthesis of probe **2**.

Synthesis of Compound 3

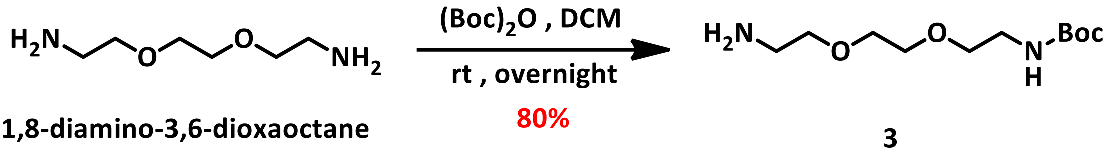

To a stirred solution of 1,8-Diamino-3,6-dioxaoctane (4.0 g, 27 mmol) in 25 mL DCM was added dropwise Di-tert-butyl dicarbonate (1.96 g, 9 mmol) in 25 mL DCM at 0 °C. The resulting mixture was stirred at room temperature for 12 hours. The mixture was extracted with DCM and water. The combined organic phase was washed with brine, dried over Na_2SO_4, filtered and concentrated to obtain the desired product as a colorless oil in 80% yield.

^{1}H NMR (500 MHz, CD_3OD) δ 3.61 (s, 4H), 3.52 (t, J = 5.2 Hz, 2H), 3.51 (t, J = 5.5 Hz, 2H), 3.22 (t, J = 5.6 Hz, 2H), 2.78 (t, J = 5.3 Hz, 2H), 1.44 (s, 9H) ppm. **^{13}C NMR** (126 MHz, CD_3OD) δ 158.41, 80.04, 73.57, 71.30, 71.24, 71.03, 42.09, 41.22, 28.76 ppm. **HRMS** (ESI) m/z: calc. for $[C_{11}H_{25}N_2O_4]^+$ $[M+H]^+$ 249.18143 found 249.18131.

Synthesis of Compound 4

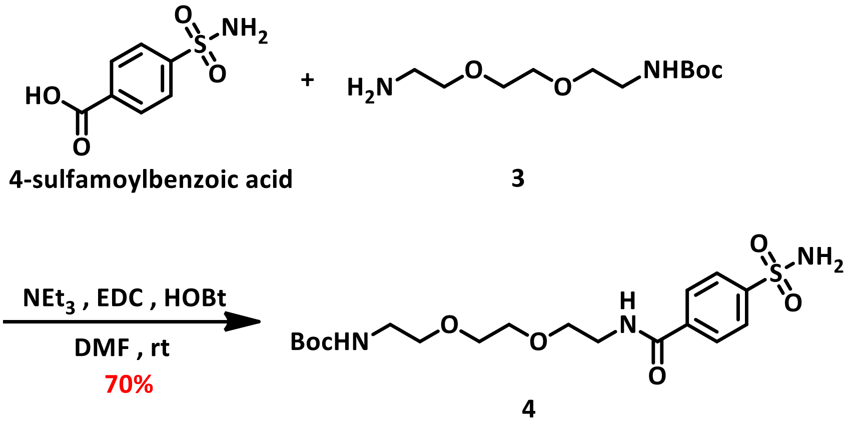

Mixture of 4-Sulfamoylbenzoic acid (81 mg, 0.40 mmol), HOBt (108 mg, 0.80 mmol), EDC·HCl (116 mg, 0.60 mmol) and NEt_3 (84 µL, 0.60 mmol) in 5 mL DMF was stirred for 10 minutes in a reaction flask. Compound **3** (100 mg, 0.40 mmol) was added and the mixture was reacted for 4 hours at room temperature. The reaction mixture

was concentrated under reduced pressure and purified by column chromatography (MeOH/DCM) to obtain the desired product as a white solid in 70% yield.

^{1}H NMR (500 MHz, CD_3OD) δ 7.97 (s, 4H), 3.70 – 3.68 (m, 2H), 3.67 – 3.64 (m, 2H), 3.63 – 3.58 (m, 4H), 3.50 (t, *J* = 5.6 Hz, 2H), 3.20 (t, *J* = 5.6 Hz, 2H), 1.42 (s, 9H) ppm. **^{13}C NMR** (176 MHz, CD_3OD) δ 168.80, 158.37, 147.53, 138.91, 129.01, 127.27, 80.10, 71.23, 71.19, 70.99, 70.38, 41.11, 40.98, 28.72 ppm. **HRMS** (ESI): m/z calc. for $[C_{18}H_{29}N_3O_7S_1Na]^+$ $[M+Na]^+$ 454.07427 found 454.07442.

Synthesis of Compound 5

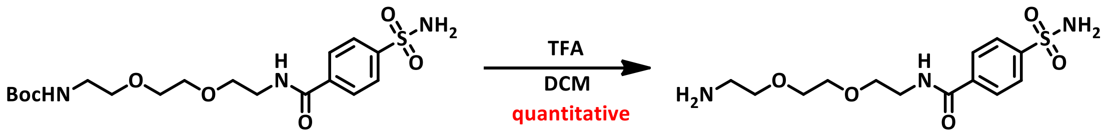

4 5

To a solution of compound **4** (100 mg, 0.23 mmol) in DCM (4 mL) was added, with ice-bath cooling, Trifluoroacetic acid (1 mL). The reaction mixture was stirred at ambient temperature for 2 hours. The reaction mixture was concentrated under reduced pressure to afford compound **5** which was used in the next step without further purification.

Synthesis of Compound 6

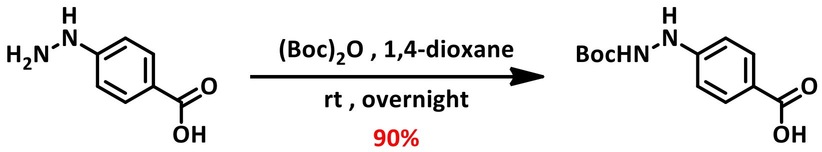

4-hydrazinylbenzoic acid 6

To a stirred solution of 4-Hydrazinobenzoic acid (1.0 g, 6.6 mmol) in 20 mL 1,4-Dioxane was added di-tert-butyl dicarbonate (1.72 g, 7.9 mmol) in 10 mL 1,4-Dioxane. The resulting mixture was stirred at room temperature for 12 hours. The reaction mixture was concentrated under reduced pressure. The residue was precipitated

using a mixture of ethyl acetate and hexane to obtain the desired product as a yellow solid in 90% yield.

^{1}H NMR (700 MHz, CD_3OD) δ 7.85 (d, *J* = 8.8 Hz, 2H), 6.76 (d, *J* = 8.8 Hz, 2H), 1.49 (s, 9H) ppm. **^{13}C NMR** (176 MHz, CD_3OD) δ 170.33, 158.67, 154.95, 132.46, 121,73, 112.05, 81.58, 28.63 ppm. **HRMS** (ESI): m/z calc. for $[C_{12}H_{15}N_2O_4]^-$ $[M\text{-}H]^-$ 251.10318 found 251.10335.

Synthesis of Compound 7

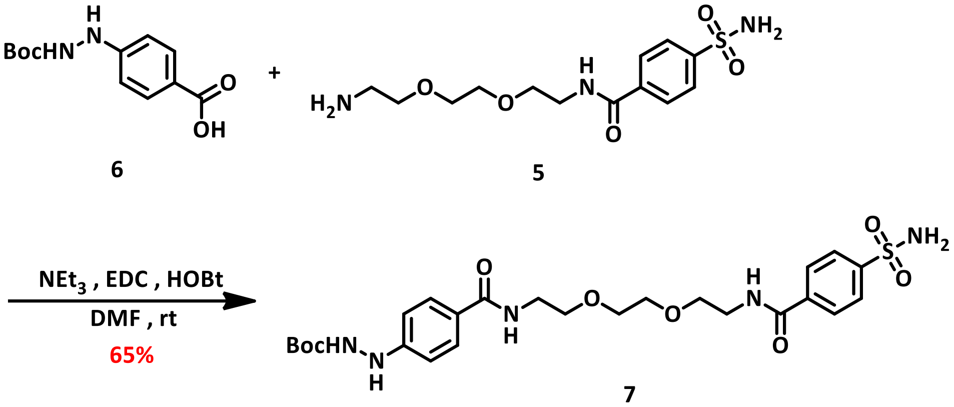

7

Mixture of compound **6** (70 mg, 0.28 mmol), HOBt (75 mg, 0.56 mmol), EDC·HCl (80 mg, 0.42 mmol) and NEt_3 (58 µL, 0.42 mmol) in 5 mL DMF was stirred for 10 minutes in a reaction flask. Compound **5** (110 mg, 0.33 mmol) was added and the mixture was reacted for 3 hours. The reaction mixture was concentrated under reduced pressure and purified by column chromatography (MeOH/DCM) to obtain the desired product as a pale yellow oil in 65% yield.

^{1}H NMR (500 MHz, CD_3OD) δ 7.95 (s, 4H), 7.68 (d, *J* = 8.8 Hz, 2H), 6.76 (d, *J* = 8.8 Hz, 2H), 3.67 – 3.65 (m, 6H), 3.64 (t, *J* = 5.6 Hz, 2H), 3.57 (t, *J* = 5.5 Hz, 2H), 3.52 (t, *J* = 5.6 Hz, 2H), 1.48 (s, 9H) ppm. **^{13}C NMR** (126 MHz, CD_3OD) δ 170.25, 168.91, 158.73, 147.63, 138.97, 129.73, 129.02, 127.32, 125.57, 112.38, 81.57, 71.33, 70.74, 70.46, 41.03, 40.71, 28.64 ppm. **HRMS** (ESI): m/z calc. for $[C_{25}H_{35}N_5Na_1O_8S_1]^+$ $[M+Na]^+$ 588.21040 found 588.21108.

Synthesis of Compound 8

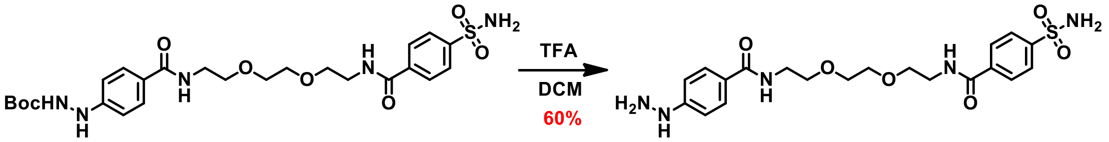

7 8

To a solution of compound **7** (100 mg, 0.18 mmol) in DCM (4 mL) was added, with ice-bath cooling, trifluoroacetic acid (1 mL). The reaction mixture was stirred at ambient temperature for 1 hour. The reaction mixture was concentrated under reduced pressure and purified by reverse-phase column chromatography (ACN/H_2O) to obtain the desired product as a pale yellow oil in 60% yield.

Synthesis of Compound 9

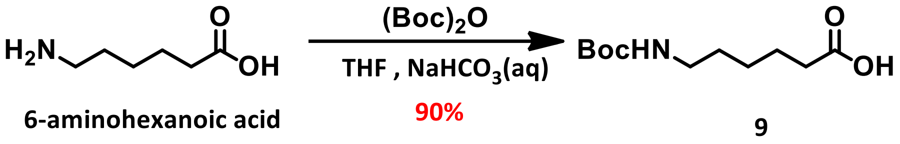

To a stirred solution of 6-Aminohexanoic acid (1.0 g, 7 mmol) in 10 mL THF and 20 mL $NaHCO_{3(aq)}$ was added dropwise Di-tert-butyl dicarbonate (2.0 g, 9.2 mmol) in 10 mL THF. The resulting mixture was stirred at room temperature for 12 hours. The reaction mixture was concentrated under reduced pressure then dissolved in HCl(aq). The mixture was extracted with DCM and water. The combined organic phase was washed with brine, dried over Na_2SO_4, filtered and concentrated to obtain the desired product as a colorless oil in 90% yield.

^{1}H NMR (500 MHz, CD_3OD) δ 3.03 (t, *J* = 7 Hz, 2H), 2.29 (t, *J* = 7.4 Hz, 2H), 1.61 (q, *J* = 7.4 Hz, 2H), 1.49 – 1.45 (m, 2H), 1.43 (s, 9H), 1.38 – 1.33 (m, 2H) ppm. **^{13}C NMR** (126 MHz, CD_3OD) δ 177.50, 158.53, 79.79, 41.17, 34.83, 30.64, 28.79, 27.38, 25.75 ppm. **HRMS** (ESI): m/z calc. for $[C_{11}H_{21}N_1Na_1O_4]^+$ $[M+Na]^+$ 254.13683 found 254.13658.

Synthesis of Compound 10

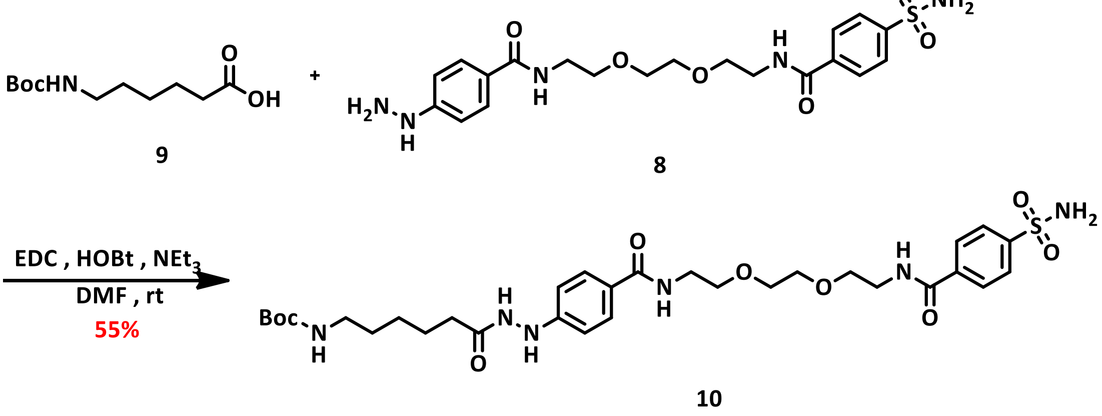

Mixture of compound **9** (42 mg, 0.18 mmol), HOBt (49 mg, 0.36 mmol), EDC·HCl (69 mg, 0.36 mmol) and NEt_3 (75 µL, 0.54 mmol) in 5 mL DMF was stirred for 10 minutes in a reaction flask. Compound **8** (100 mg, 0.21 mmol) was added and the mixture was reacted for 1 hour. The reaction mixture was concentrated under reduced pressure and purified by reverse-phase column chromatography (ACN/H_2O) to obtain the desired product as a white solid in 55% yield.

^{1}H NMR (700 MHz, CD_3OD) δ 7.95 (d, *J* = 3.2 Hz, 4H), 7.69 (d, *J* = 8.8 Hz, 2H), 6.78 (d, *J* = 8.8 Hz, 2H), 3.68 – 3.66 (m, 6H), 3.64 (t, *J* = 5.6 Hz, 2H), 3.57 (t, *J* = 5.5 Hz, 2H), 3.52 (t, *J* = 5.6 Hz, 2H), 3.06 – 3.03 (m, 2H), 2.30 (t, *J* = 7.4 Hz, 2H), 1.69 (quint, *J* = 7.4 Hz, 2H), 1.51 (quint, *J* = 7 Hz, 2H), 1.43 (s, 9H), 1.41 – 1.38 (m, 2H) ppm. **^{13}C NMR** (176 MHz, CD_3OD) δ 175.99, 170.17, 168.91, 158.59, 153.22, 147.67, 138.94, 129.76, 129.03, 127.32, 125.91, 112.74, 79.86, 71.34, 70.74, 70.47, 41.18, 41.04, 40.73, 34.82, 30.64, 28.79, 27.48, 26.40 ppm. **HRMS** (ESI): m/z calc. for $[C_{31}H_{46}N_6Na_1O_9S_1]^+$ $[M+Na]^+$ 701.29447 found 701.29481.

Synthesis of Compound 11

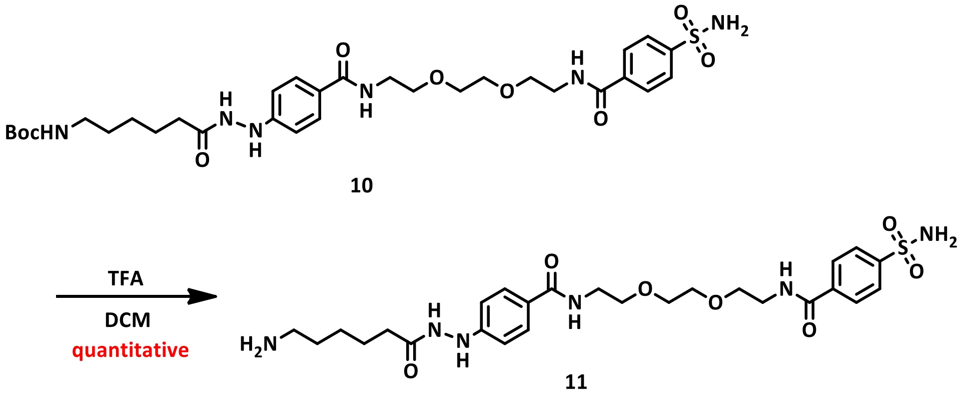

To a solution of compound **10** (100 mg, 0.15 mmol) in DCM (4 mL) was added, with ice-bath cooling, trifluoroacetic acid (1 mL). The reaction mixture was then stirred at ambient temperature for 2 hours. The reaction mixture was concentrated under reduced pressure to afford compound **11** which was used in the next step without further purification.

Synthesis of Probe 1

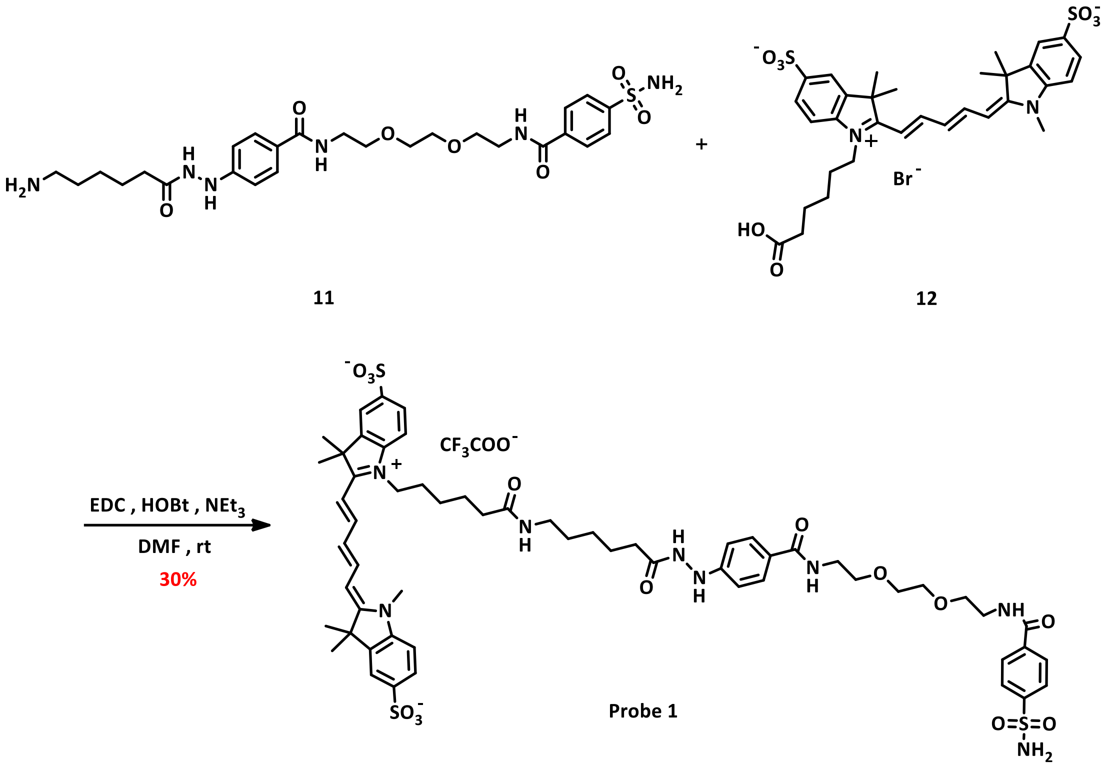

Mixture of compound **12** (10 mg, 0.019 mmol), HOBt (5.5 mg, 0.041 mmol), EDC·HCl (7.9 mg, 0.041 mmol) and NEt_3 (8.6 µL, 0.061 mmol) in 3 mL DMF was stirred for 10 minutes in a reaction flask. Compound **11** (34 mg, 0.059 mmol) was added and the mixture was reacted for 6 hours. The reaction mixture was concentrated under reduced pressure and purified by reverse-phase column chromatography (ACN/H_2O) to obtain the desired product as a blue solid in 30% yield.

^{1}H NMR (700 MHz, CD_3OD) δ 8.32 – 8.26 (m, 2H), 7.94 (s, 4H), 7.90 – 7.87 (m, 4H), 7.68 (d, J = 8.9 Hz, 2H), 7.32 (d, J = 8.9 Hz, 2H), 6.76 (d, J = 8.9 Hz, 2H), 6.71 – 6.63 (m, 1H), 6.35 – 6.29 (m, 2H), 4.09 (t, J = 7.3 Hz, 2H) , 3.67 – 3.62 (m, 11H), 3.56 (t, J = 5.5 Hz, 2H) , 3.51 (t, J = 5.6 Hz, 2H) , 3.16 (t, J = 7.2 Hz, 2H) , 2.30 (t, J = 7.4 Hz, 2H) , 2.19 (t, J = 7.4 Hz, 2H) , 1.84 – 1.78 (m, 2H), 1.75 (s, 6H), 1.73 (s, 6H) , 1.68 (quint, J = 7 Hz, 4H), 1.52 – 1.47 (m, 2H), 1.45 – 1.36 (m, 4H) ppm. **^{13}C NMR** (176 MHz, CD3OD) δ 176.00, 175.84, 175.40, 175.15, 170.08, 168.85, 156.15, 156.12, 153.24, 147.62, 145.57, 144.94, 143.40, 143.33, 142.57, 142.49, 138.94, 129.80, 129.05, 128.01, 127.66, 127.33, 125.77, 121.35, 121.24, 116.85, 115.24, 112.77, 112.34, 111.67, 111.46, 71.34, 70.70, 70.44, 50.52, 45.00, 41.04, 40.78, 40.19, 36.70, 35.81, 34.73, 33.64, 31.78,

30.03, 28.21, 28.13, 27.87, 27.69, 27.49, 27.30, 26.53, 26.31, 25.94 ppm. **HRMS** (ESI): m/z calc. for $[C_{58}H_{75}N_8O_{14}S_3]^+$ $[M+H]^+$ 1203.4565 found 1203.4559.

Synthesis of Compound 14

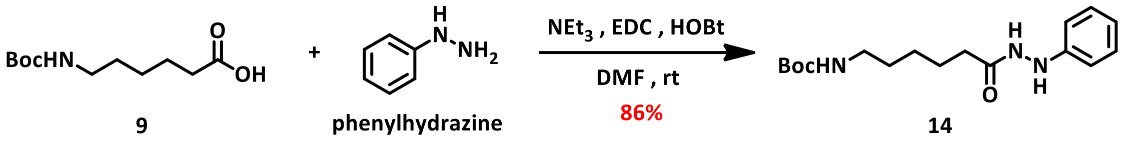

Mixture of compound **9** (30 mg, 0.13 mmol), HOBt (35 mg, 0.26 mmol), EDC·HCl (49 mg, 0.26 mmol) and NEt_3 (54 µL, 0.39 mmol) in 5 mL DMF was stirred for 10 minutes in a reaction flask. Phenylhydrazine (15 µL, 0.16 mmol) was added and the mixture was reacted for 2 hours. The reaction mixture was concentrated under reduced pressure then purified by column chromatography (MeOH/DCM) to obtain the desired product as a pale yellow oil in 86% yield.

^{1}H NMR (400 MHz, CD_3OD) δ 7.19 – 7.15 (m, 2H), 6.81 – 6.77 (m, 3H), 3.07 – 3.02 (m, 2H), 2.28 (t, J = 7.4 Hz, 2H), 1.69 (quint, J = 7.4 Hz, 2H), 1.55 – 1.47 (m, 2H), 1.43 (s, 9H), 1.42 – 1.39 (m, 2H) ppm. **^{13}C NMR** (176 MHz, CD_3OD) δ 175.84, 158.45, 149.85, 129.91, 121.01, 114.03, 79.77, 41.15, 34.83, 30.55, 28.79, 27.40, 26.43 ppm. **HRMS** (ESI): m/z calc. for $[C_{17}H_{28}N_3O_3]^+$$[M+H]^+$ 322.2125 found 322.2125.

Synthesis of Compound 15

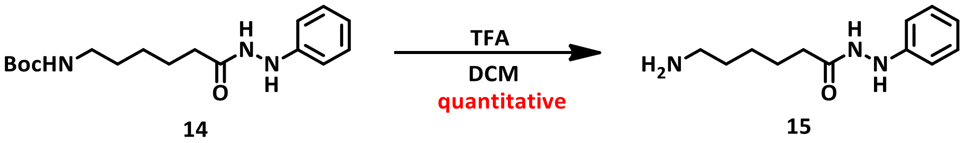

To a solution of compound **14** (100 mg, 0.31 mmol) in DCM (4 mL) was added, with ice-bath cooling, trifluoroacetic acid (1 mL). The reaction mixture was then stirred at ambient temperature for 1 hour. The reaction mixture was concentrated under reduced pressure to afford compound **15** which was used in the next step without further purification.

Synthesis of Probe 2

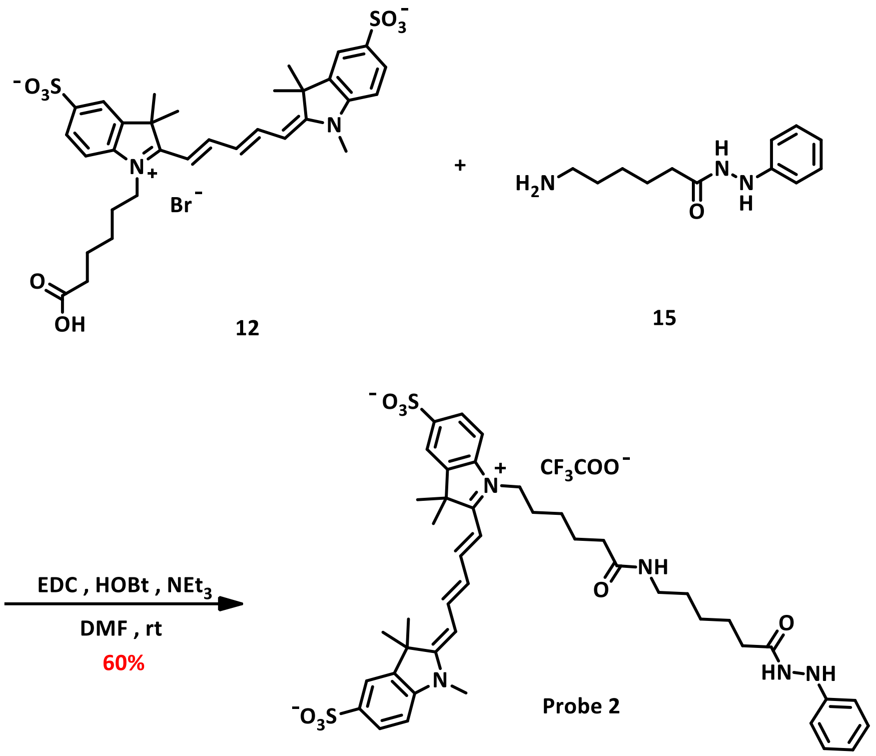

Mixture of compound **12** (30 mg, 0.077 mmol), HOBt (21 mg, 0.16 mmol), EDC·HCl (30 mg, 0.16 mmol) and NEt_3 (33 μL, 0.23 mmol) in 3 mL DMF was stirred for 10 minutes in a reaction flask. Compound **15** (40 mg, 0.38 mmol) was added and the mixture was reacted for 4 hours. The reaction mixture was concentrated under reduced pressure then purified by reverse-phase column chromatography (ACN/H_2O) to obtain the desired product as a blue solid in 60% yield.

^{1}H NMR (500 MHz, CD_3OD) δ 8.30 (d, *J* = 15 Hz, 2H), 7.90 – 7.87 (m, 4H), 7.32 (d, *J* = 8 Hz, 2H), 7.16 (t, *J* = 7.6 Hz, 3H), 6.78 (d, *J* = 7.6 Hz, 3H), 6.67 (t, *J* = 12.4 Hz, 1H), 6.32 (d, *J* = 13.5 Hz, 1H), 4.11 (t, *J* = 7.5 Hz, 2H), 3.63 (s, 3H), 3.20 – 3.10 (m, 2H), 2.28 (t, *J* = 7 Hz, 2H), 2.19 (t, *J* = 7 Hz, 2H), 1.81 (t, *J* = 7.6 Hz, 2H), 1.74 (s, 14H), 1.68 (t, *J* = 8 Hz, 4H), 1.53 – 1.36 (m, 4H) ppm. **^{13}C NMR** (176 MHz, CD_3OD) δ 176.06, 175.84, 175.21, 175.15, 156.22, 156.12, 156.11, 145.58, 144.96, 144.93, 143.43, 143.36, 142.58, 142.50, 129.98, 128.06, 128.02, 127.62, 121.36, 121.25, 121.25, 114.21, 111.65, 111.62, 111.46, 50.53, 44.99, 40.25, 36.64, 34.80, 34.64, 31.76, 30.01, 28.13, 27.86, 27.68, 27.51, 27.43, 27.33, 26.54, 26.38, 25.62 ppm. **HRMS** (ESI): m/z calc. for $[C_{44}H_{56}N_5O_8S_2]^+$ $[M+H]^+$ 846.3571 found 846.3565.

Synthesis of Probe 3

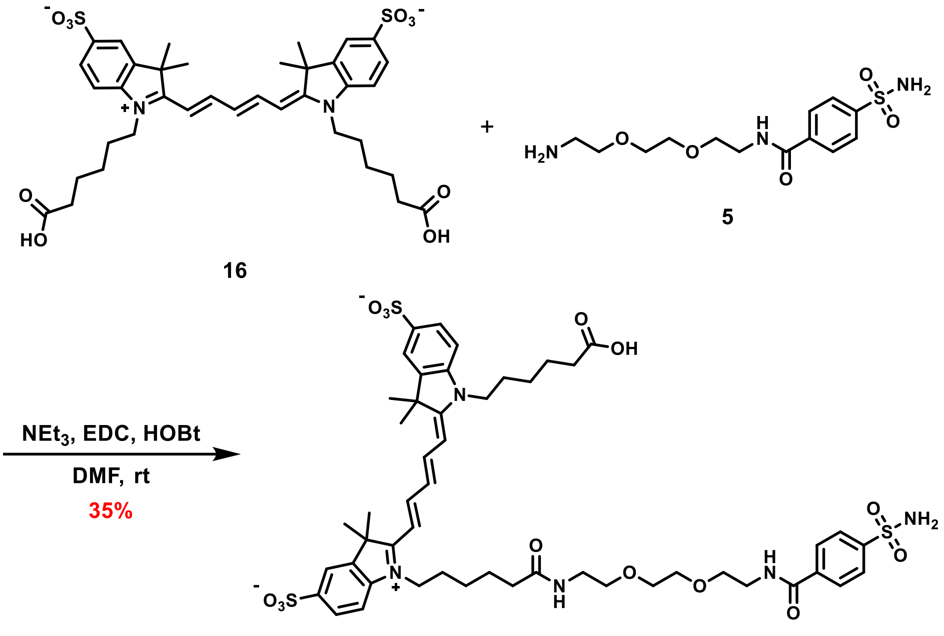

Mixture of compound **16** (50 mg, 0.067 mmol), HOBt (20.5 mg, 0.13 mmol), EDC·HCl (25.8 mg, 0.13 mmol) and NEt_3 (28 μL, 0.20 mmol) in 3 mL DMF was stirred for 10 minutes in a reaction flask. Compound **5** (7.4 mg, 0.033 mmol) was added and the mixture was reacted for 4 hours. The reaction mixture was concentrated under reduced pressure then purified by reverse-phase column chromatography (ACN/H_2O) to obtain the desired product as a blue solid in 35% yield.

^{1}H NMR (700 MHz, CD_3OD) δ 8.33 – 8.28 (m, 2H), 7.98 – 7.95 (m, 4H), 7.90 – 7.87 (m, 4H), 7.34 (quint, *J* = 4.2 Hz, 2H), 6.70 (t, *J* = 12.3 Hz, 1H), 6.34 (d, *J* = 13.7 Hz, 1H), 6.33 (d, *J* = 13.7 Hz, 1H), 4.15 – 4.10 (m, 4H), 3.67 (t, *J* = 5.5 Hz, 2H), 3.65 – 3.63 (m, 2H), 3.62 – 3.59 (m, 2H), 3.58 (t, *J* = 5.6 Hz, 2H), 3.50 (t, *J* = 5.3 Hz, 2H), 3.30 (t, *J* = 5.6 Hz, 2H), 2.33 – 2.30 (m, 2H), 2.18 – 2.15 (m, 2H), 1.85 – 1.81 (m, 4H), 1.754 (s, 3H), 1.75 (s, 3H), 1.74 (s, 6H), 1.70 – 1.65 (m, 4H), 1.50 – 1.47 (m, 2H), 1.42 (quint, *J* = 7.8 Hz, 2H) ppm. **^{13}C NMR** (176 MHz, CD_3OD) δ 177.32, 175.95, 175.79, 175.32, 168.81, 156.19, 147.68, 147.55, 144.92, 143.29, 142.61, 138.89, 129.07, 128.05, 127.83, 127.34, 121.44, 121.34, 111.67, 105.29, 71.30, 71.25, 70.52, 70.37, 50.58, 49.52, 45.01, 40.98, 40.24, 36.55, 34.59, 34.47, 28.11, 28.07, 27.83, 27.25, 26.43, 25.63 ppm. **HRMS** (ESI): m/z calc. for $[C_{50}H_{64}N_5O_{14}S_3]^{2-}$ $[M]^{2-}$ 527.1801 found 527.1783.

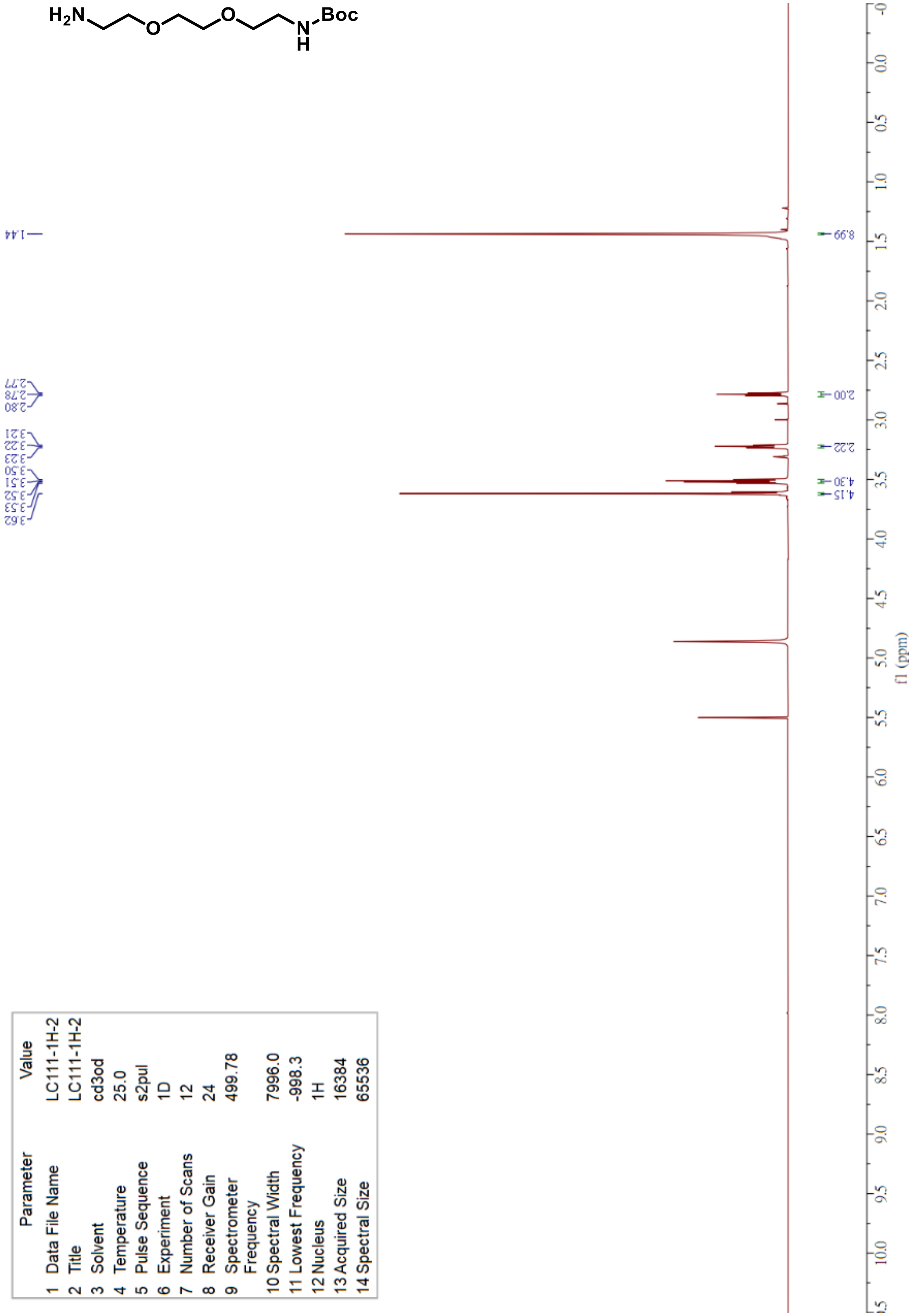

Figure S26 ^{1}H NMR spectra of compound **3**.

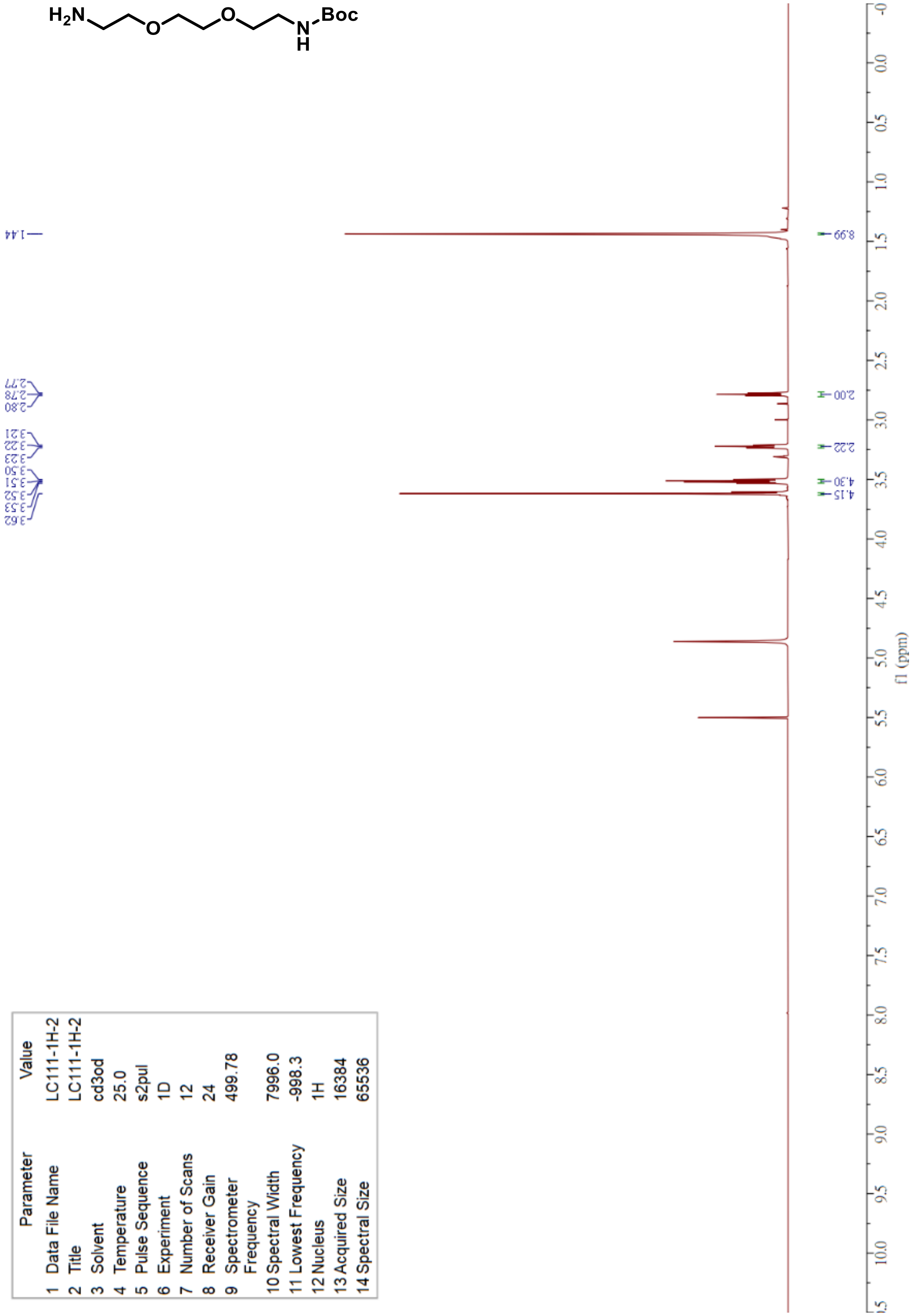

Figure S27 ^{13}C NMR spectra of compound **3**.

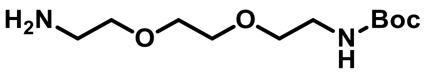

Data:LC111 Acquired:2/17/2023 1:26:11 PM
Comment: Operator:AccuTOF
Description: m/z Calibration File:20230112-TFANa_...
Ionization Mode:ESI+ Created:2/17/2023 5:15:29 PM
History:Average(MS[1] 0.39..0.41) Created by:AccuTOF

Charge number:1 Tolerance:200.00[ppm], 250.00 .. 250.... Unsaturation Number:-100.5 .. 200.0 (...
Element:^{12}C:11 .. 11, ^{1}H:20 .. 25, ^{35}Cl:0 .. 1, ^{14}N:2 .. 2, ^{23}Na:0 .. 1, ^{16}O:4 .. 4

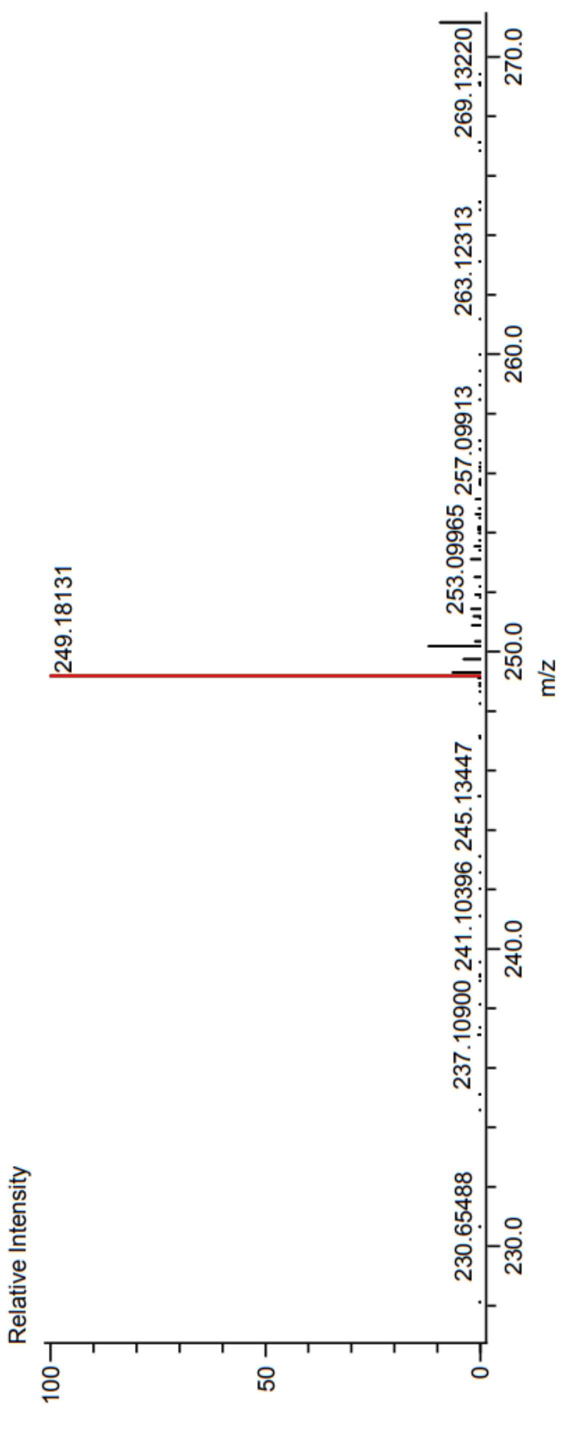

Mass	Intensity	Calc. Mass	Mass Difference [mDa]	Mass Difference [ppm]	Possible Formula
249.18131	471163.50	249.18143	-0.12	-0.49	$^{12}C_{11}{}^{1}H_{25}{}^{14}N_2{}^{16}O_4$

Figure S28 HRMS-ESI spectra of compound **3**.

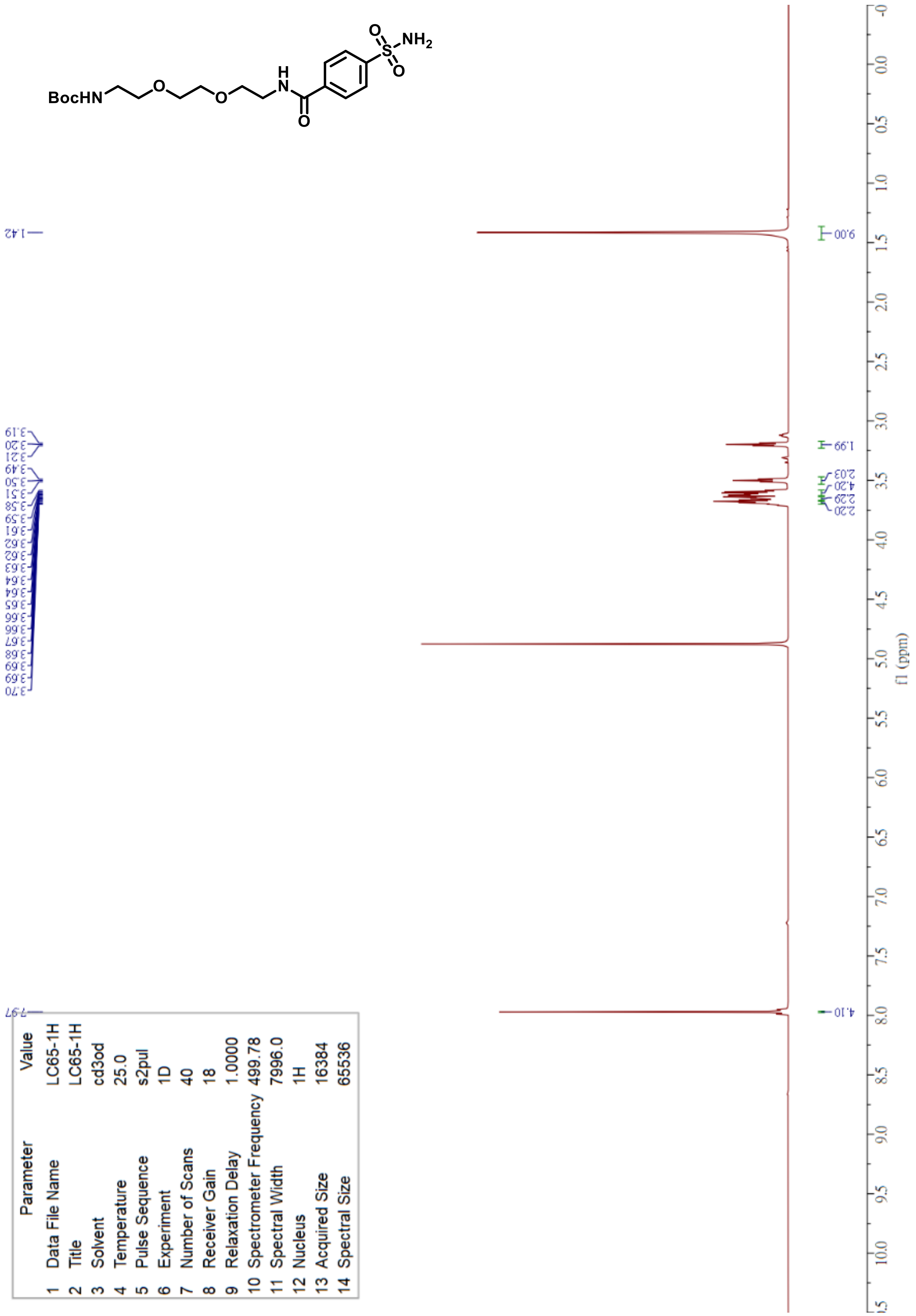

Figure S29 ^{1}H NMR spectra of compound **4**.

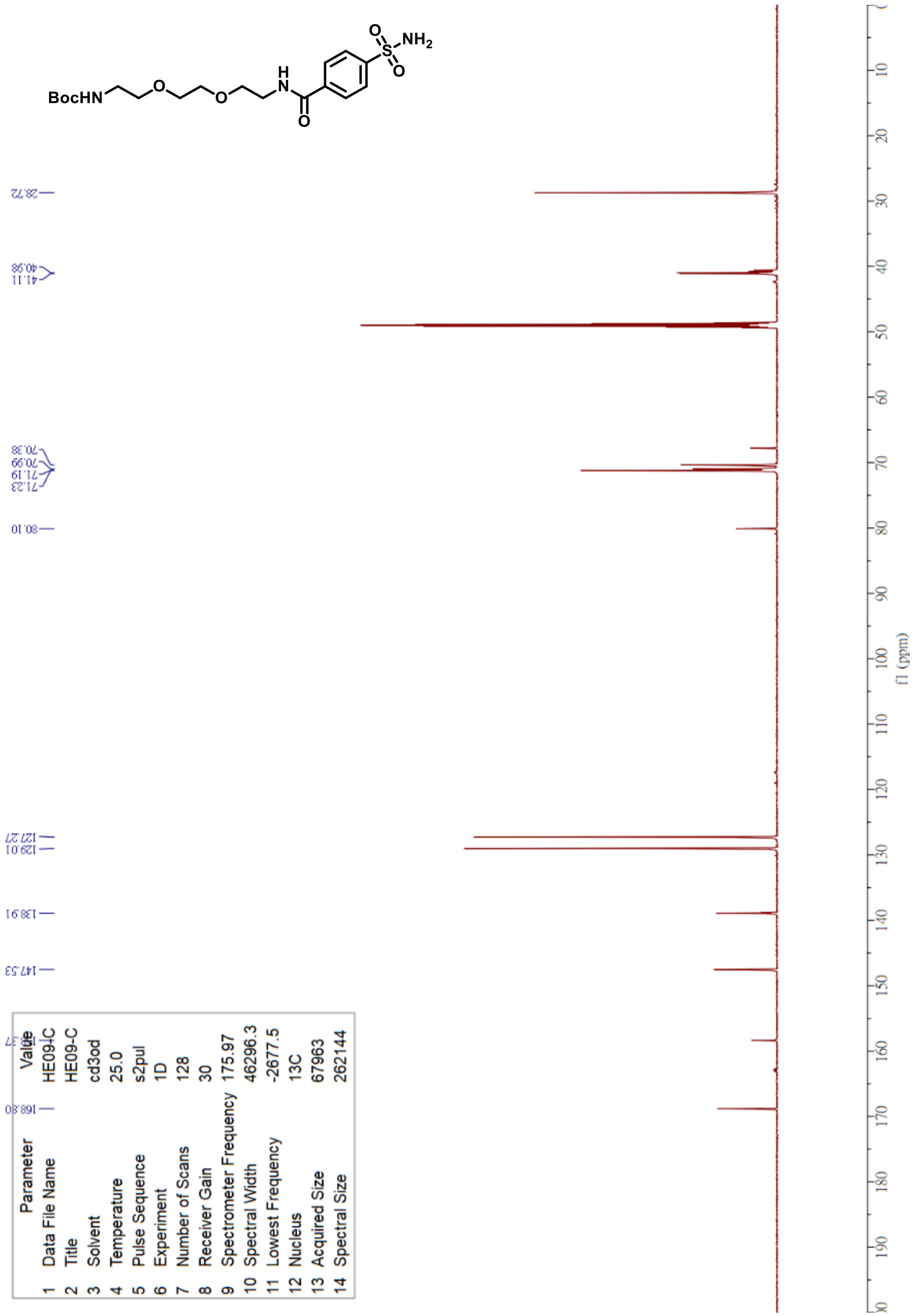

Figure S30 ^{13}C NMR spectra of compound **4**.

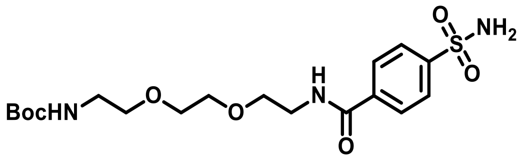

Data:LC65
Comment:
Description:
Ionization Mode:ESI+
History:Average(MS[1] 0.17..0.22)

Acquired:3/24/2023 3:56:25 PM
Operator:AccuTOF
m/z Calibration File:20230317-TFANa_...
Created:3/25/2023 9:37:26 AM
Created by:AccuTOF

Charge number:1
Tolerance:300.00[ppm], 300.00 .. 300....
Unsaturation Number:-200.5 .. 200.0 (...
Element: ^{12}C:18 .. 18, ^{1}H:0 .. 30, ^{14}N:3 .. 3, ^{23}Na:0 .. 1, ^{16}O:7 .. 7, ^{32}S:2 .. 2

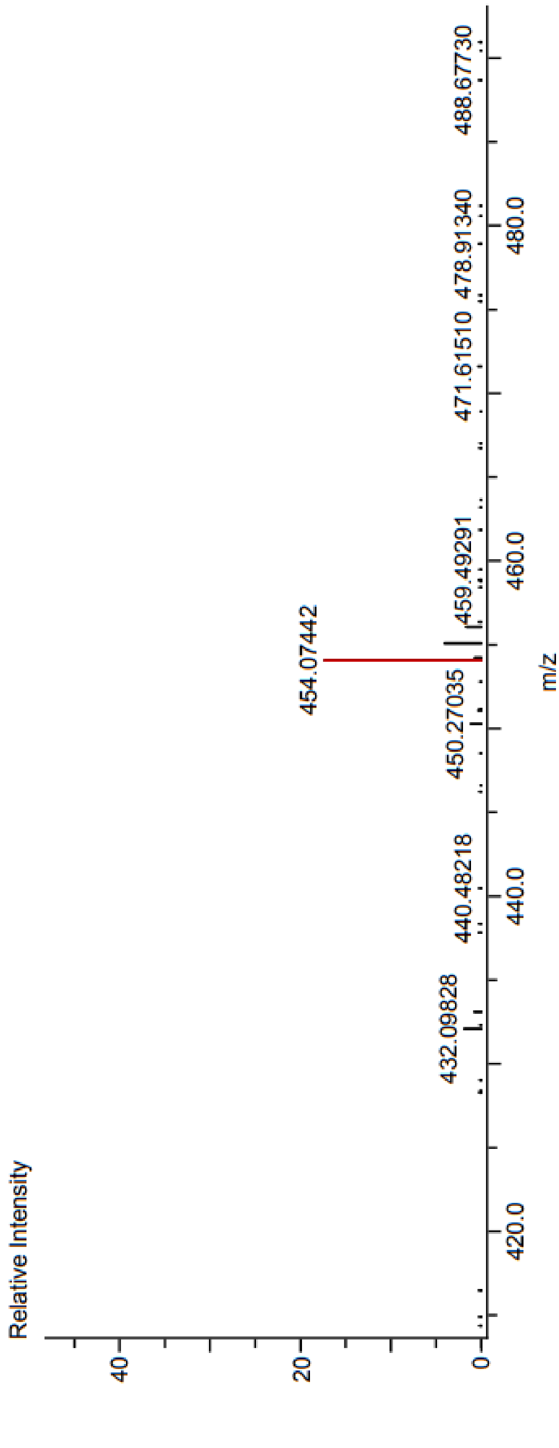

Mass	Intensity	Calc. Mass	Mass Difference [mDa]	Mass Difference [ppm]	Possible Formula
454.07442	11151.76	454.07427	0.15	0.34	$^{12}C_{18}{}^{1}H_{20}{}^{14}N_{3}{}^{16}O_{7}{}^{32}S_{2}$

Figure S31 HRMS-ESI spectra of compound **4**.

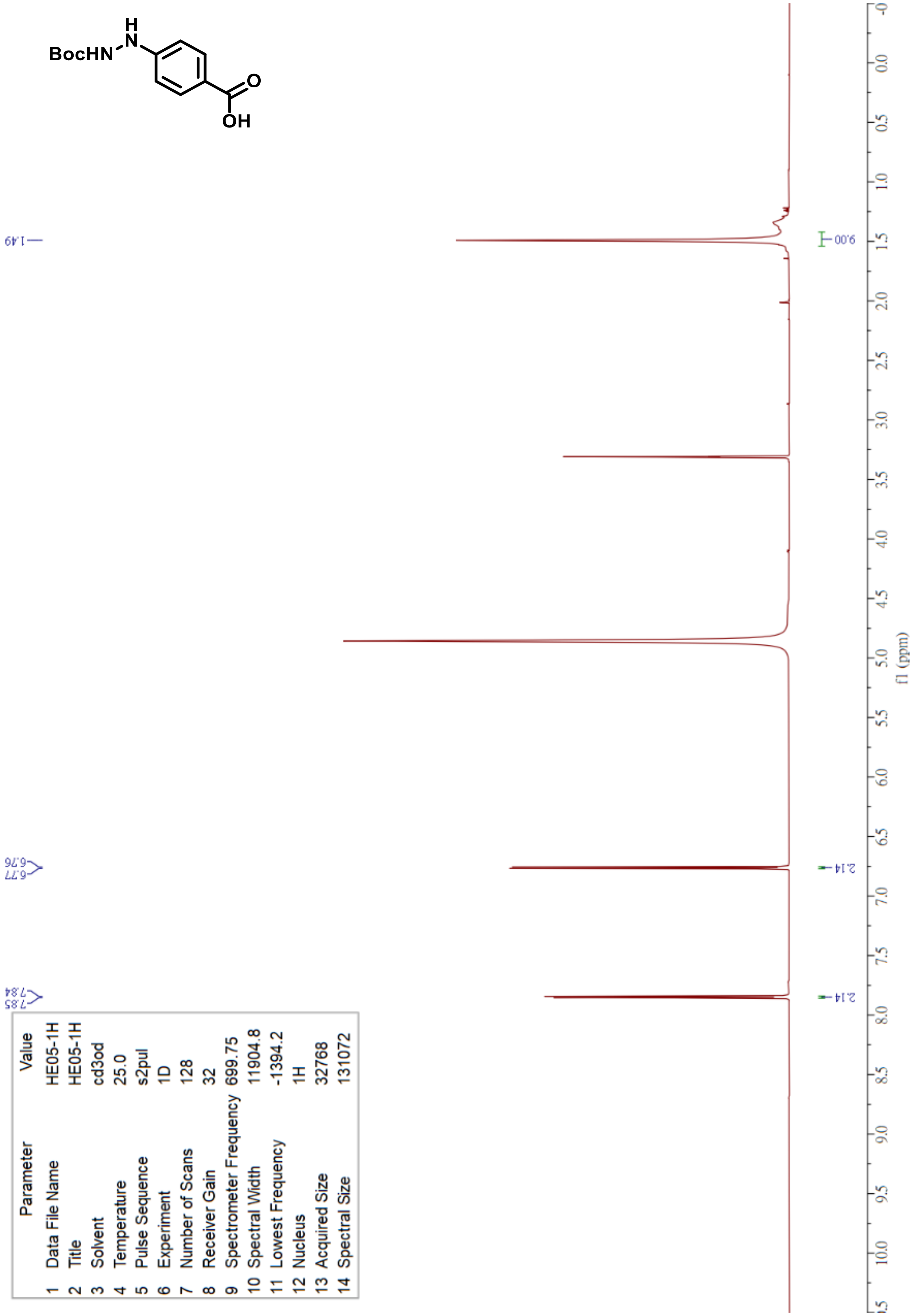

Figure S32 ^{1}H NMR spectra of compound **6**.

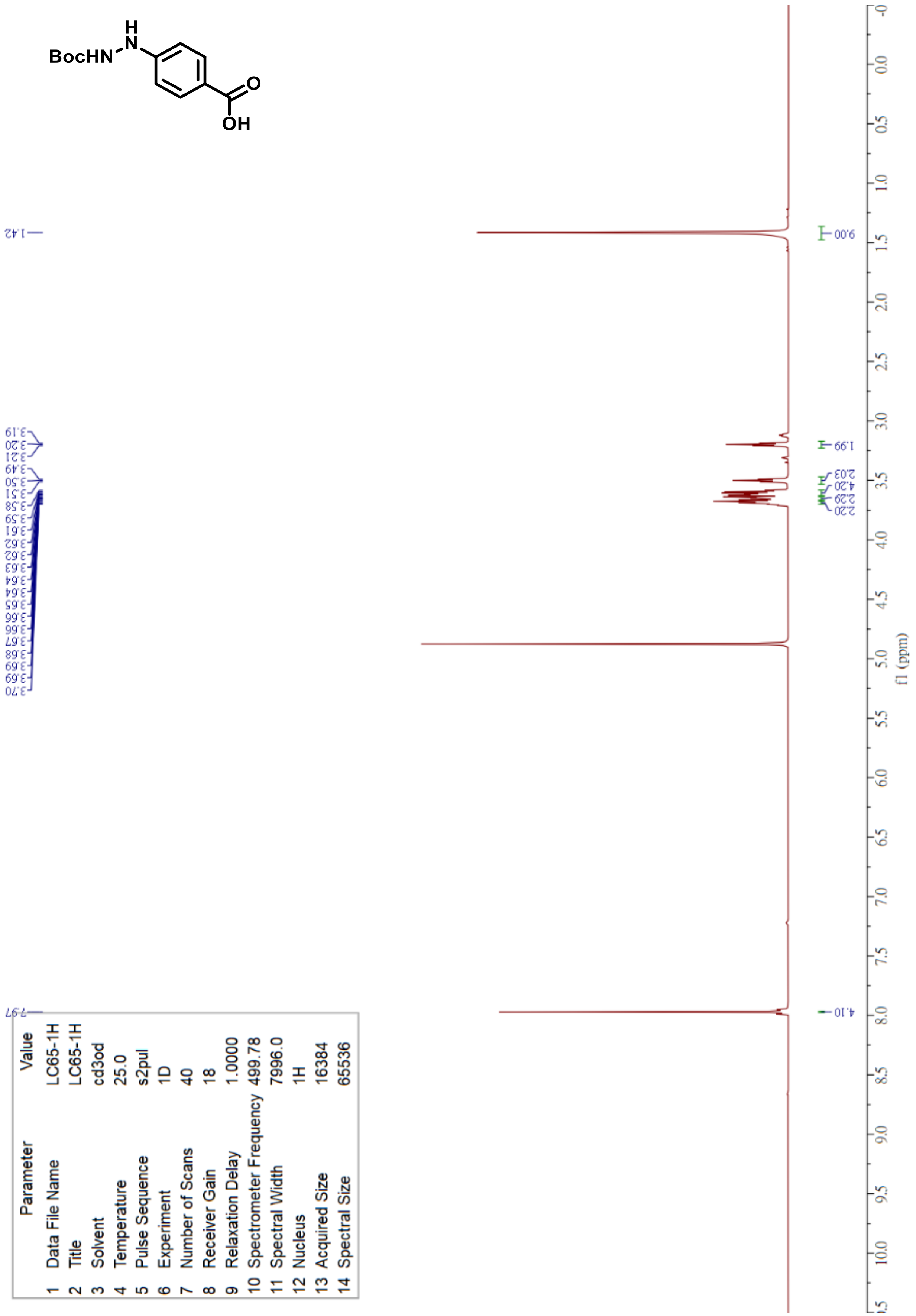

Figure S33 ^{13}C NMR spectra of compound **6**.

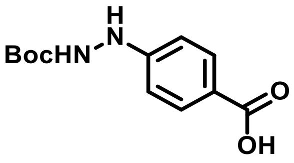

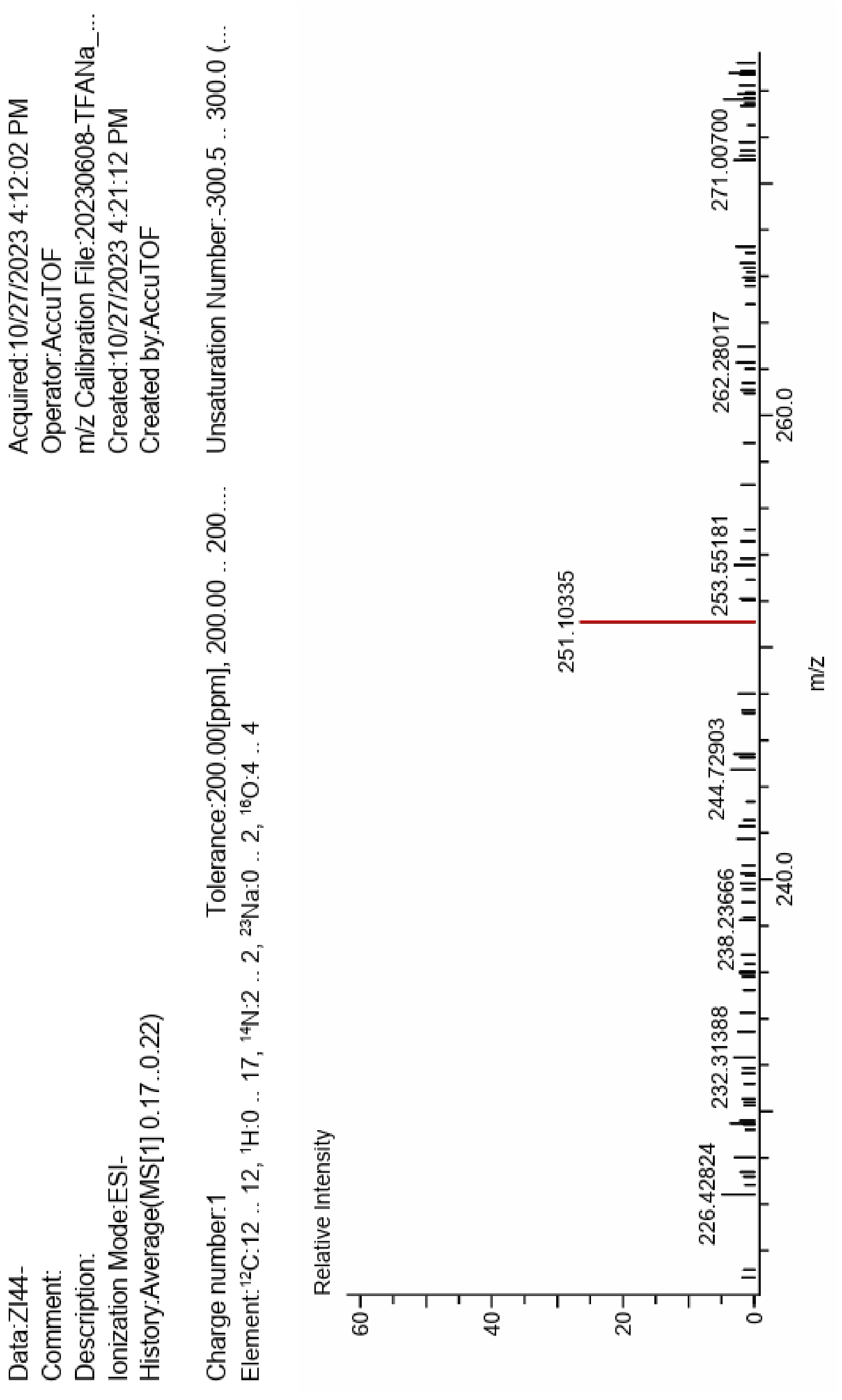

Mass	Intensity	Calc. Mass	Mass Difference [mDa]	Mass Difference [ppm]	Possible Formula
251.10335	1078.19	251.10318	0.16	0.65	$^{12}C_{12}{}^{1}H_{15}{}^{14}N_{2}{}^{16}O_{4}$

Figure S34 HRMS-ESI spectra of compound **6**.

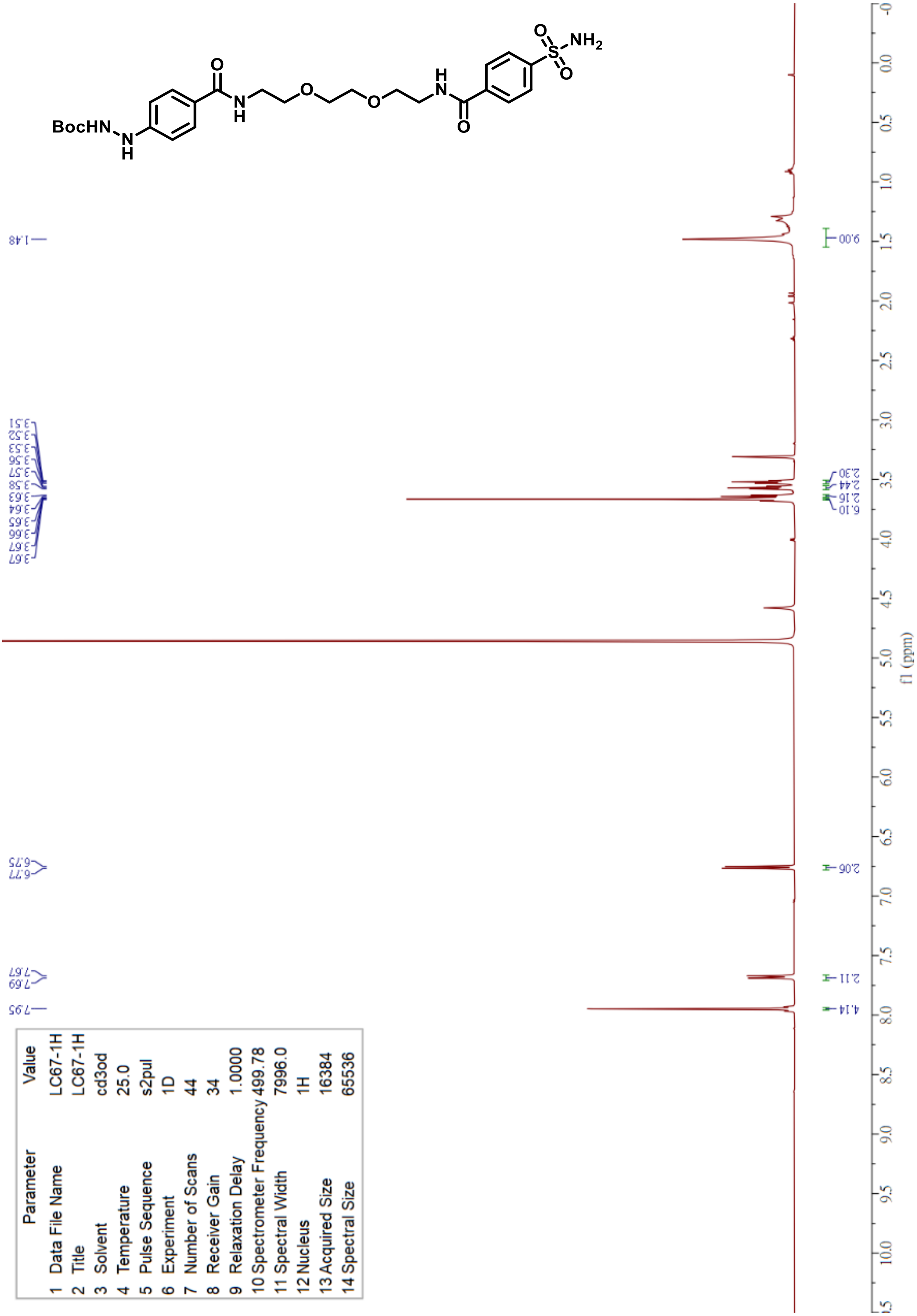

Figure S35 ^{1}H NMR spectra of compound **7**.

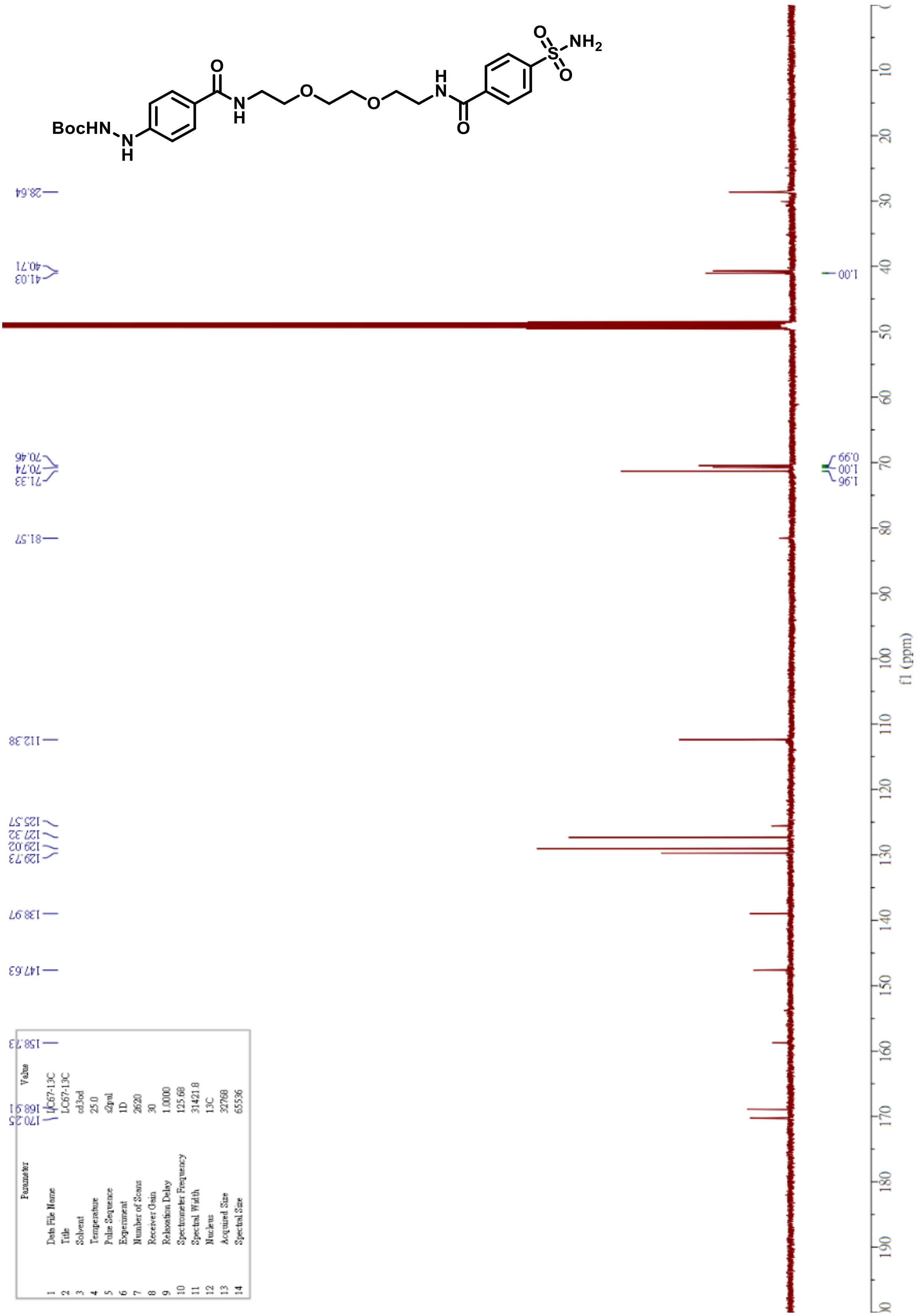

Figure S36 ^{13}C NMR spectra of compound **7**.

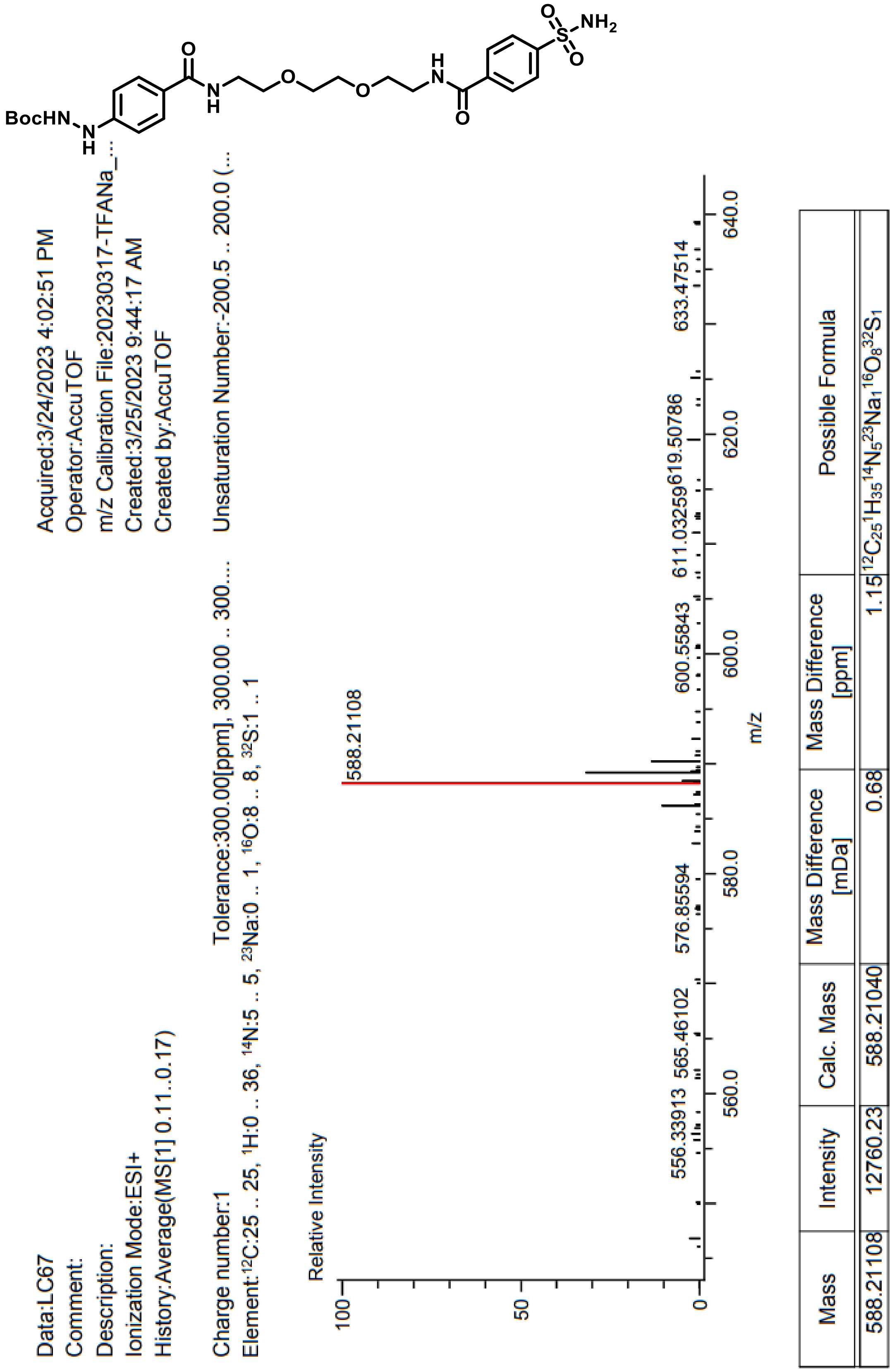

Figure S37 HRMS-ESI spectra of compound **7**.

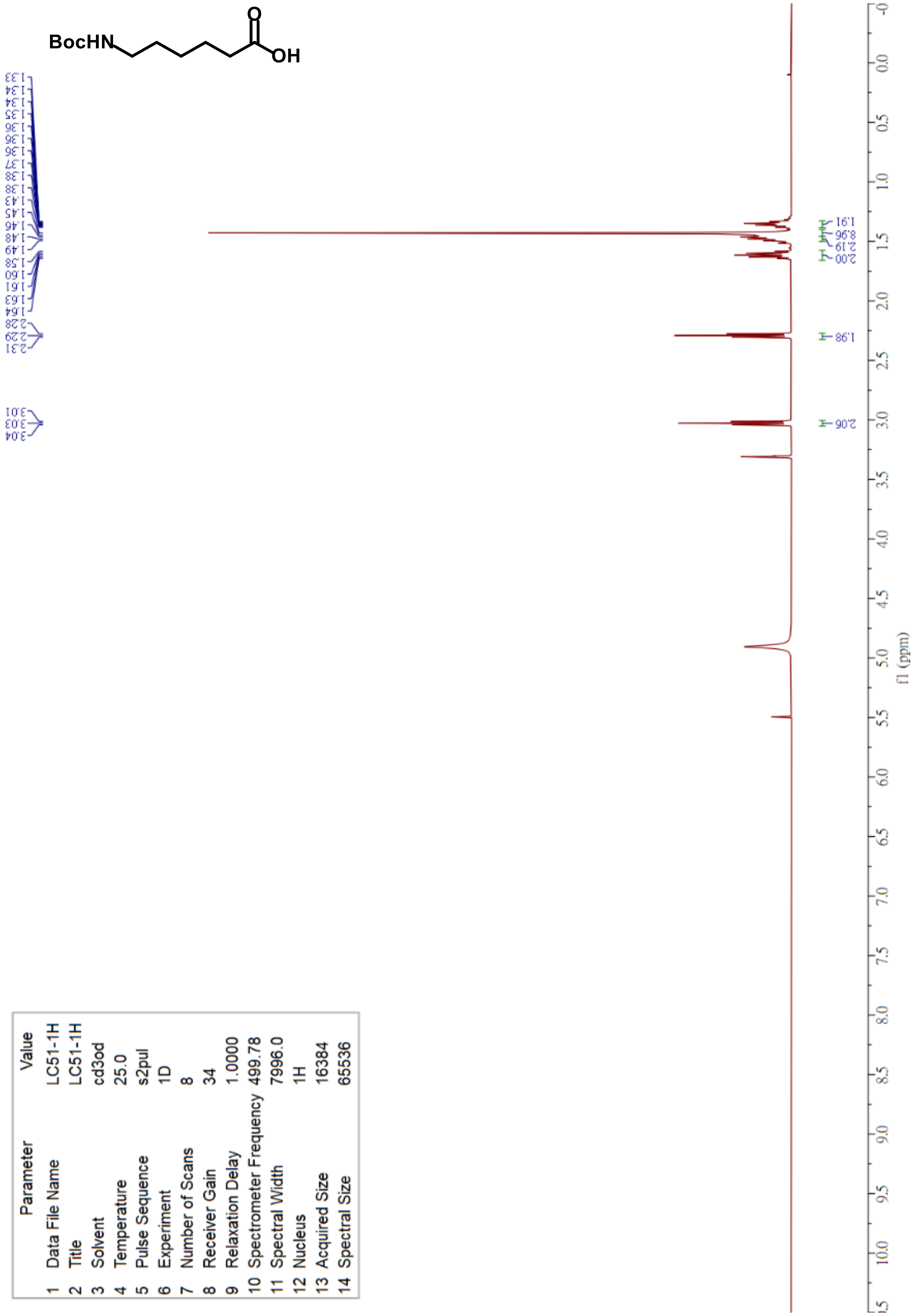

Figure S38 ^{1}H NMR spectra of compound **9**.

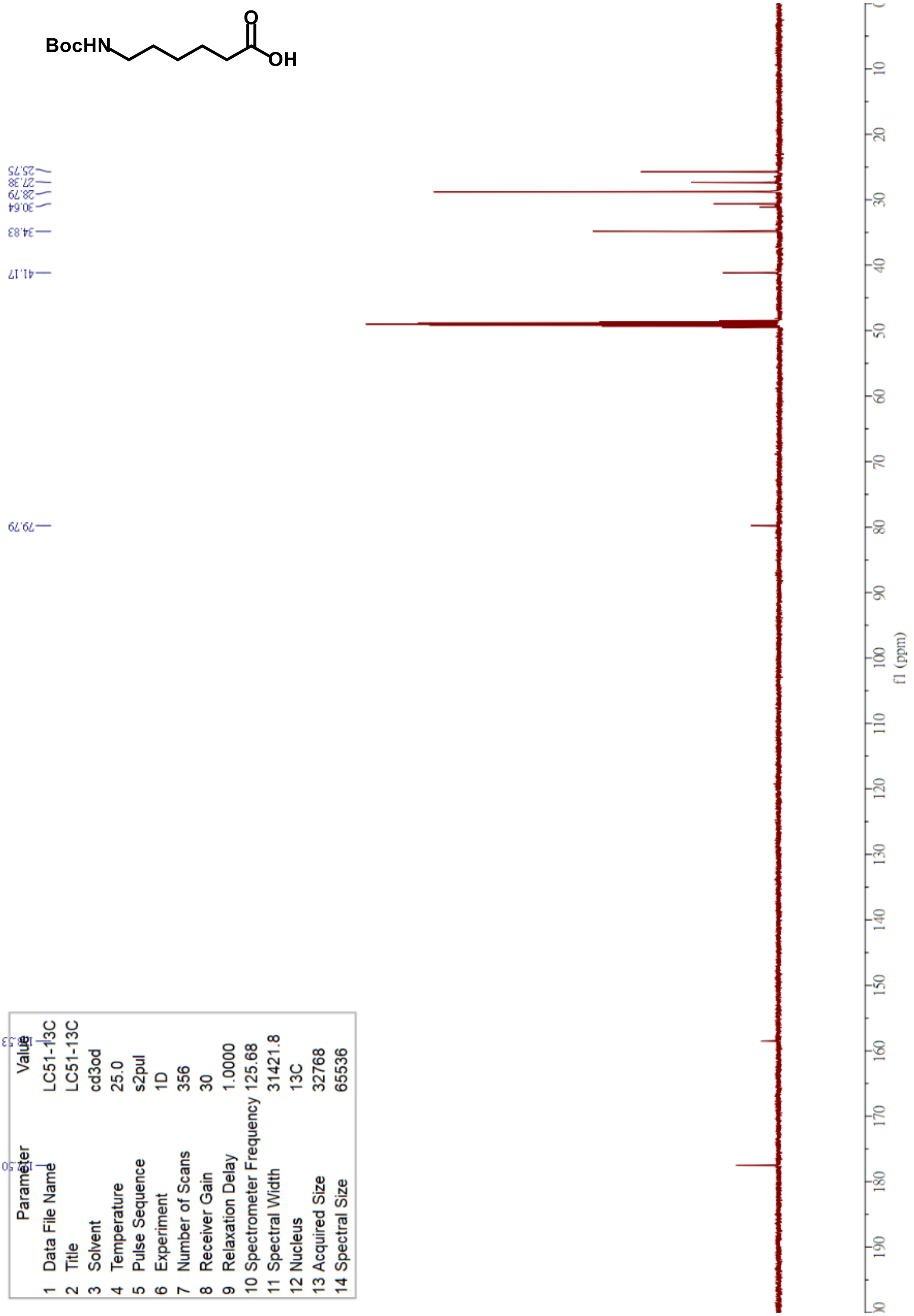

Figure S39 ^{13}C NMR spectra of compound **9**.

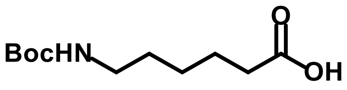

Data:LC51 Acquired:3/24/2023 4:05:53 PM
Comment: Operator:AccuTOF
Description: m/z Calibration File:20230317-TFANa_...
Ionization Mode:ESI+ Created:3/25/2023 9:46:22 AM
History:Average(MS[1] 0.11..0.11) Created by:AccuTOF

Charge number:1 Tolerance:300.00[ppm], 300.00 .. 300.... Unsaturation Number:-200.5 .. 200.0 (...
Element:^{12}C:11 .. 11, ^{1}H:0 .. 22, ^{14}N:1 .. 1, ^{23}Na:0 .. 1, ^{16}O:4 .. 4

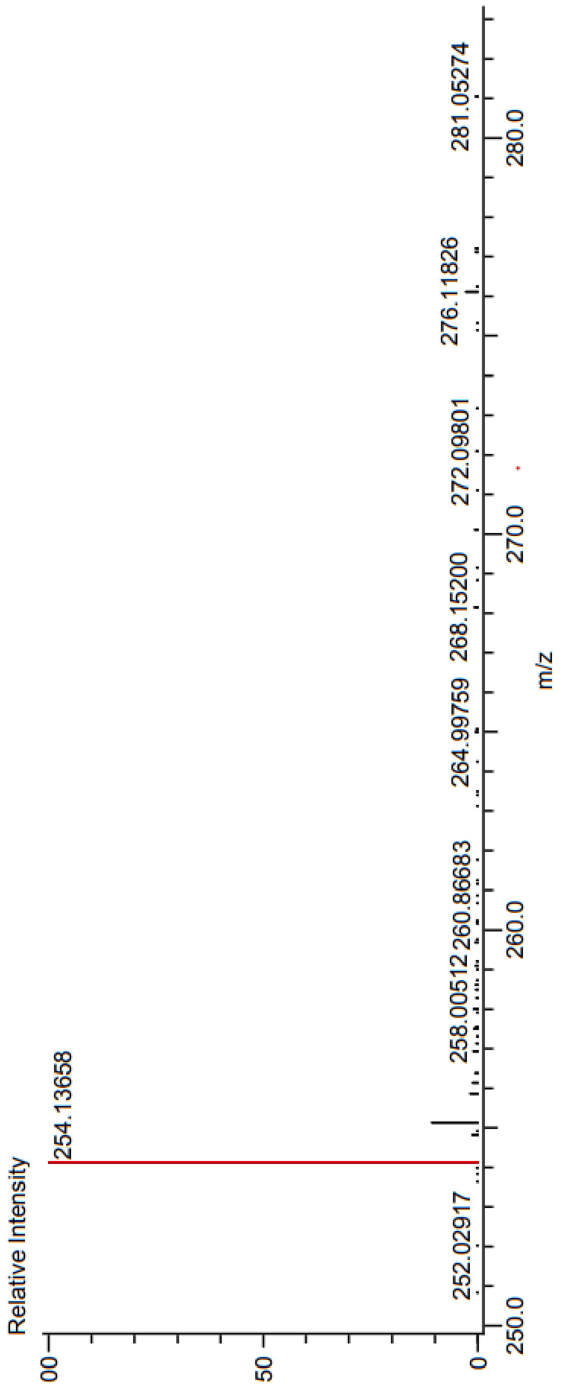

Mass	Intensity	Calc. Mass	Mass Difference [mDa]	Mass Difference [ppm]	Possible Formula
254.13658	408454.47	254.13683	-0.25	-0.98	$^{12}C_{11}{}^{1}H_{21}{}^{14}N_{1}{}^{23}Na_{1}{}^{16}O_{4}$

Figure S40 HRMS-ESI spectra of compound **9**.

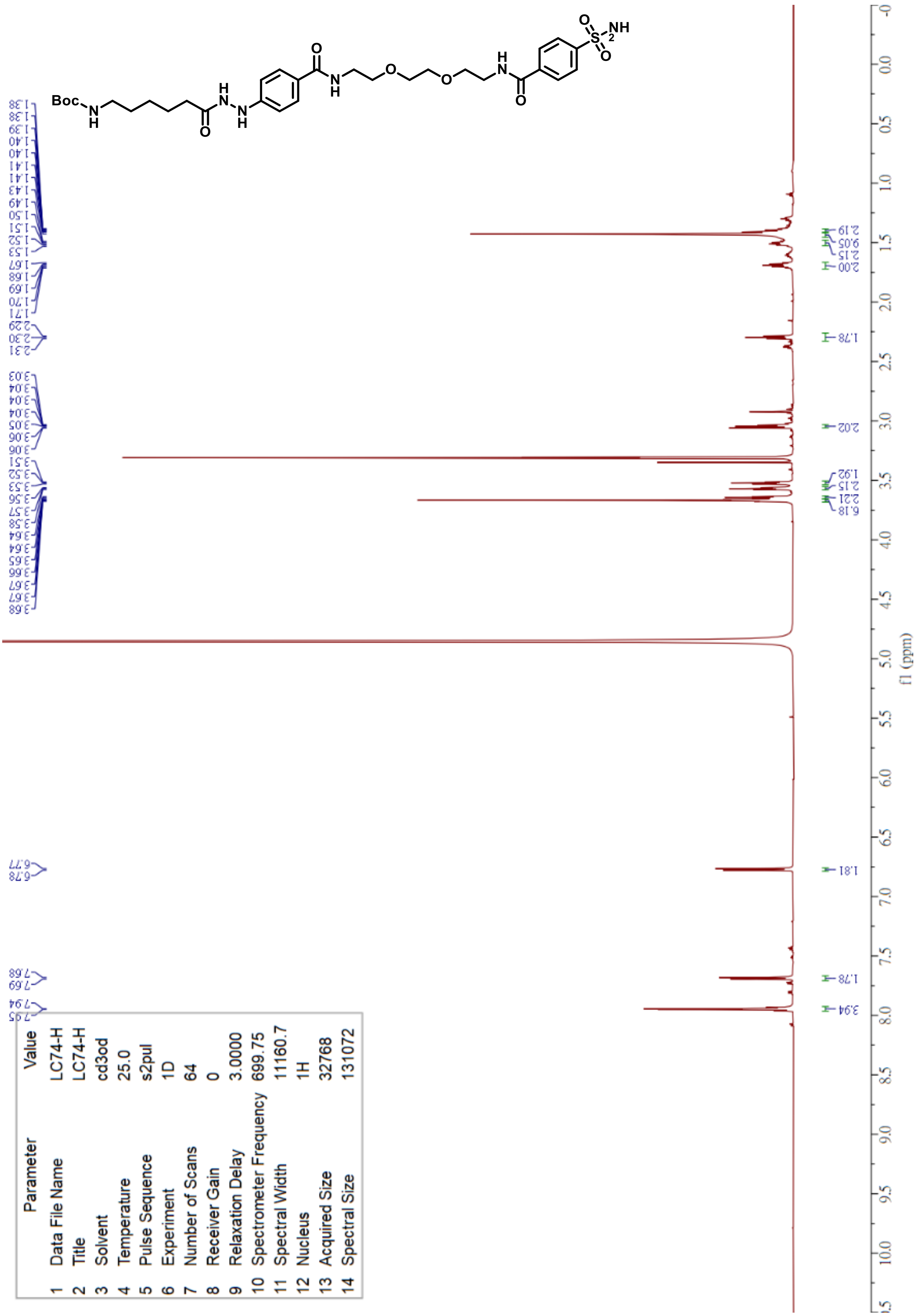

Figure S41 ^{1}H NMR spectra of compound **10**.

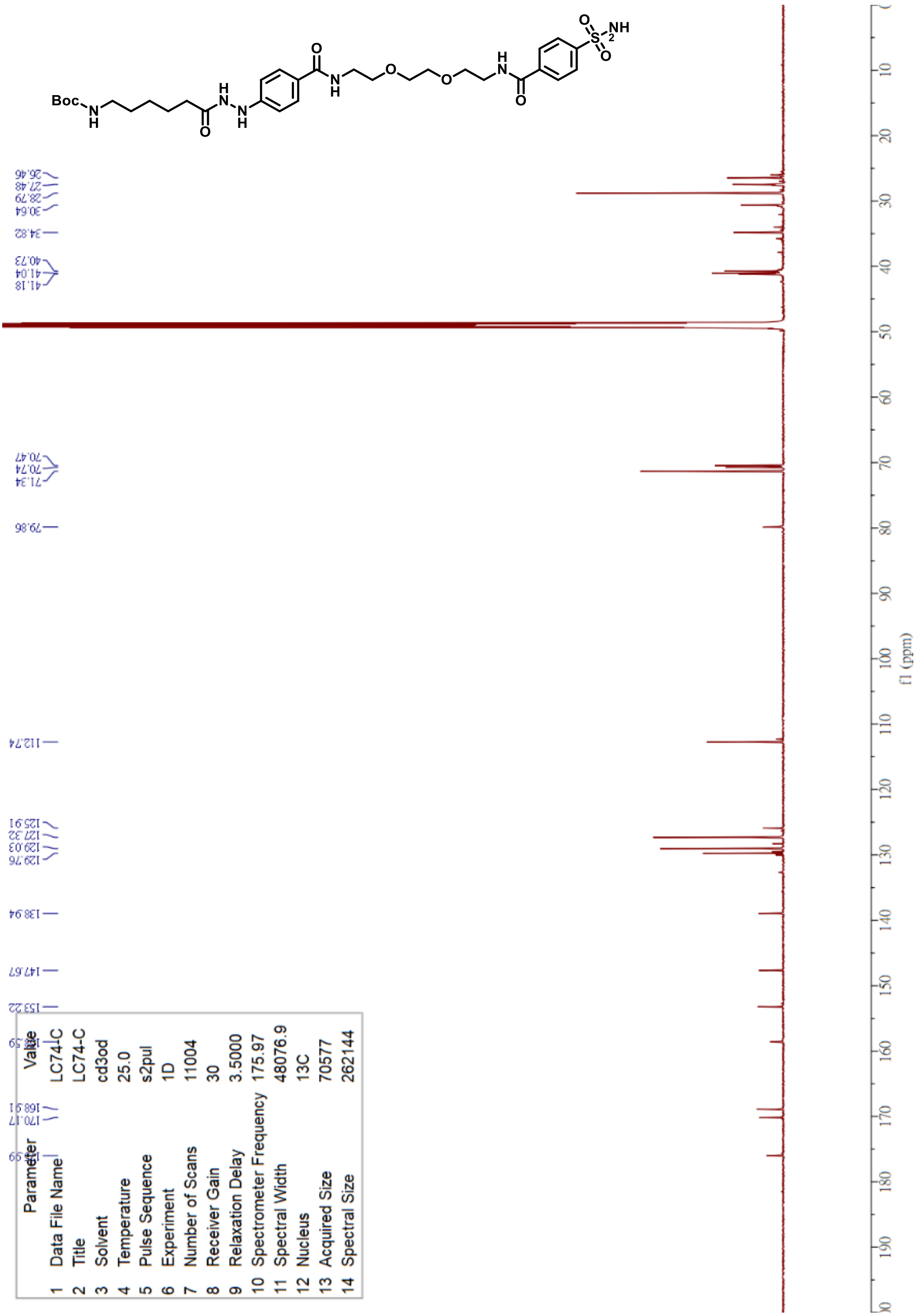

Figure S42 ^{13}C NMR spectra of compound **10**.

Data:LC74
Comment:
Description:
Ionization Mode:ESI+
History:Average(MS[1] 0.21..0.26)

Acquired:3/24/2023 3:59:53 PM
Operator:AccuTOF
m/z Calibration File:20230317-TFANa_...
Created:3/25/2023 9:43:30 AM
Created by:AccuTOF

Charge number:1
Tolerance:300.00[ppm], 300.00 .. 300....
Unsaturation Number:-200.5 .. 200.0 (...
Element:^{12}C:31 .. 31, ^{1}H:0 .. 47, ^{14}N:6 .. 6, ^{23}Na:0 .. 1, ^{16}O:9 .. 9, ^{32}S:1 .. 1

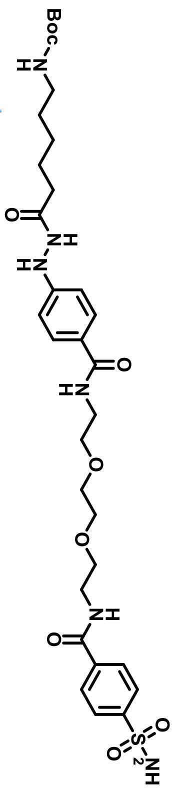

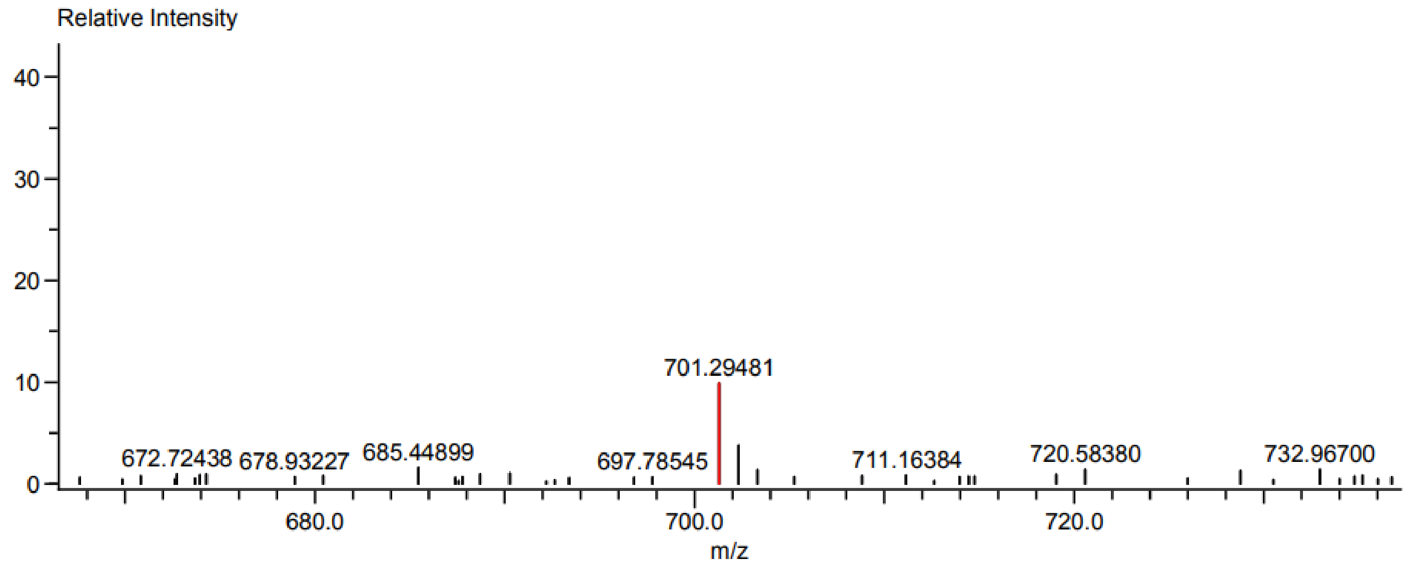

Mass	Intensity	Calc. Mass	Mass Difference [mDa]	Mass Difference [ppm]	Possible Formula
701.29481	1887.21	701.29447	0.34	0.49	$^{12}C_{31}{}^{1}H_{46}{}^{14}N_{6}{}^{23}Na_{1}{}^{16}O_{9}{}^{32}S_{1}$

Figure S43 HRMS-ESI spectra of compound **10**.

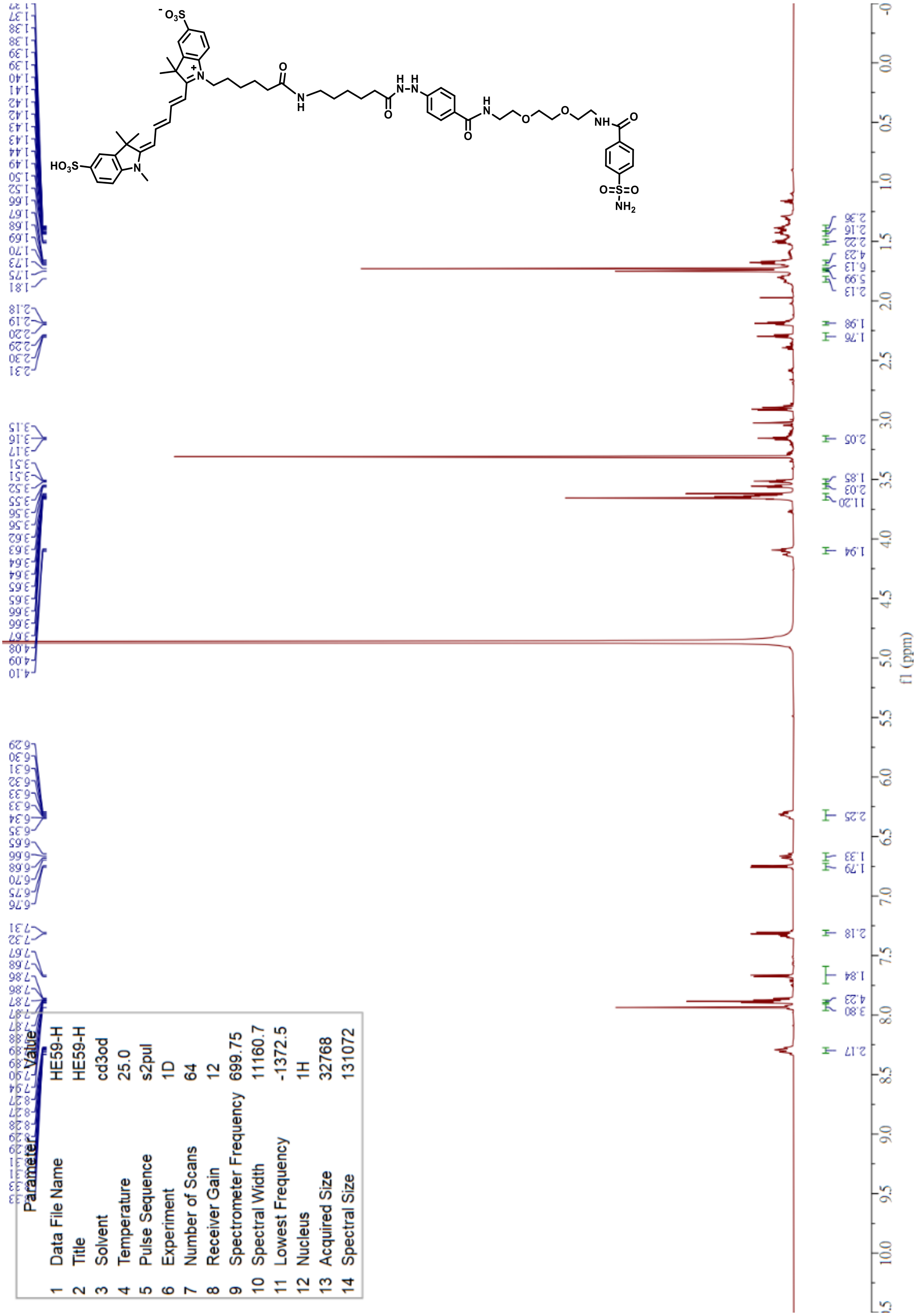

Figure S44 ^{1}H NMR spectra of **Probe 1**.

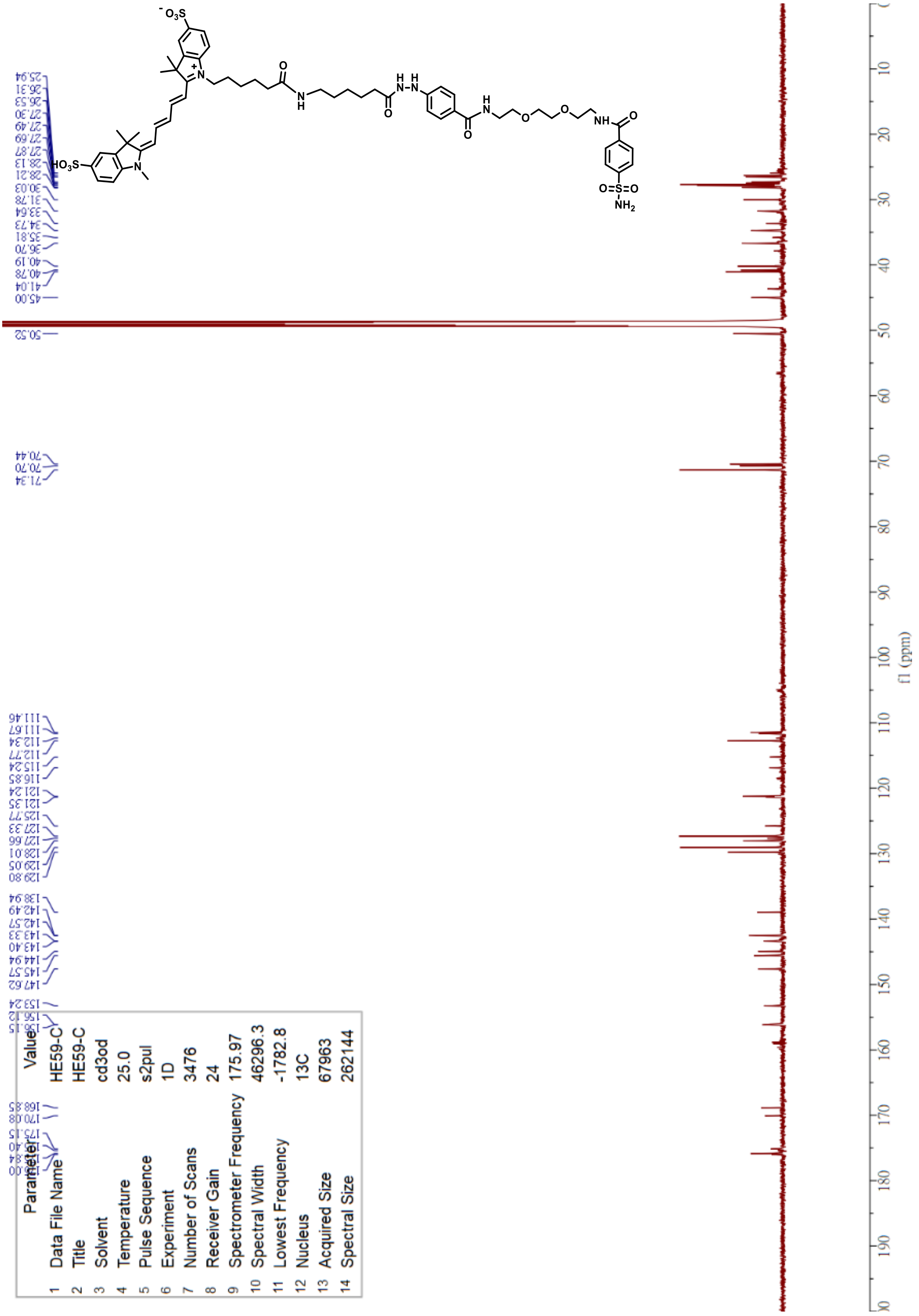

Figure S45 ^{13}C NMR spectra of **Probe 1**.

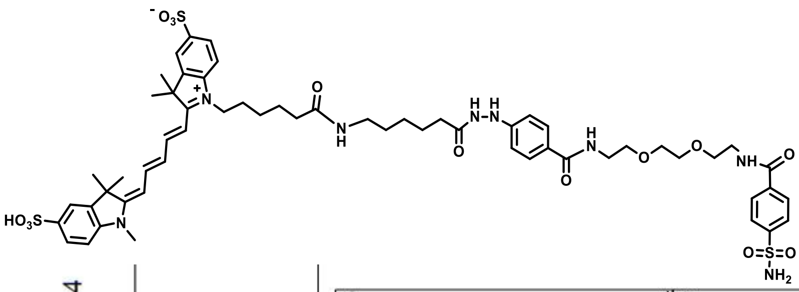

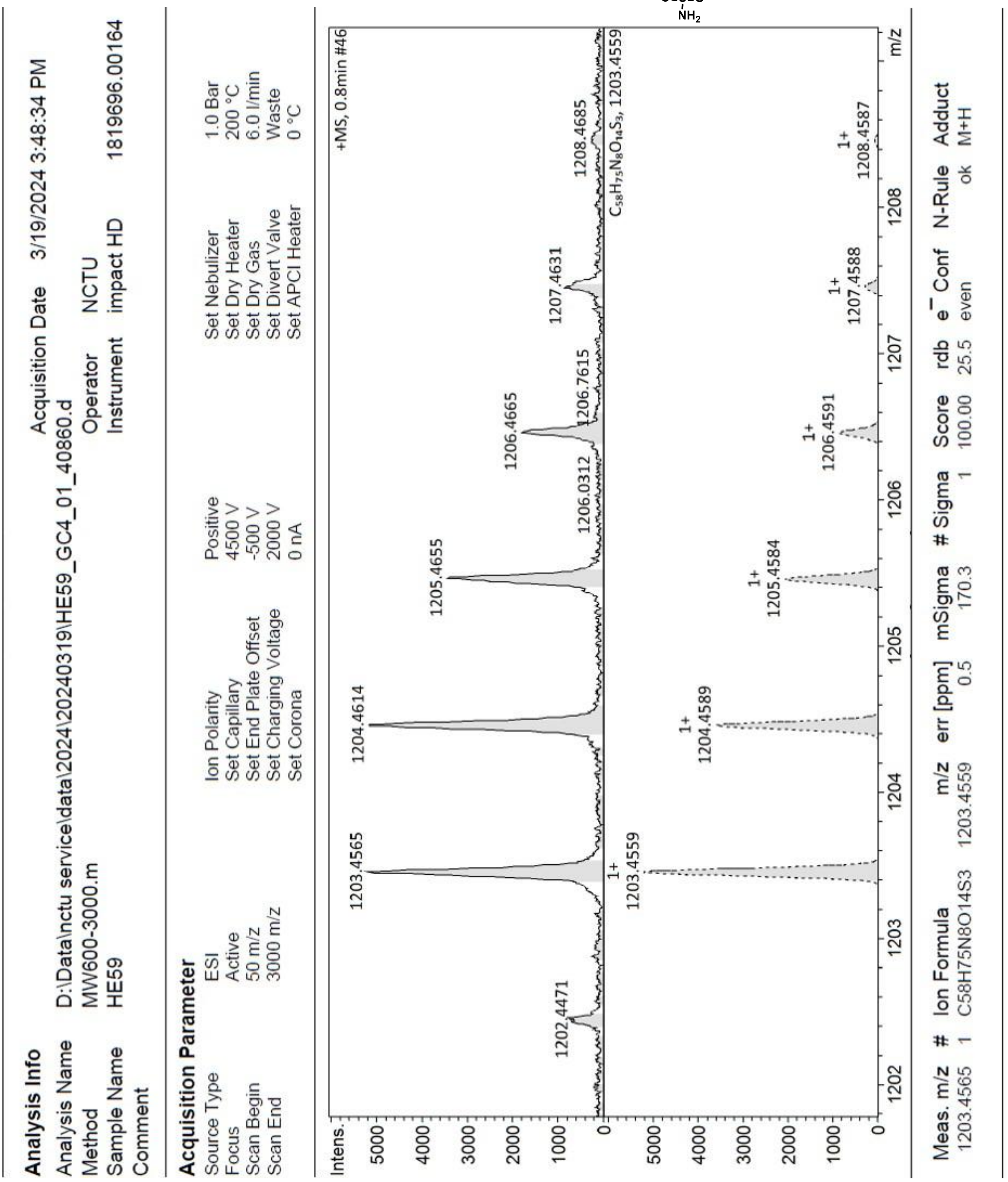

Figure S46 HRMS-ESI spectra of **Probe 1**.

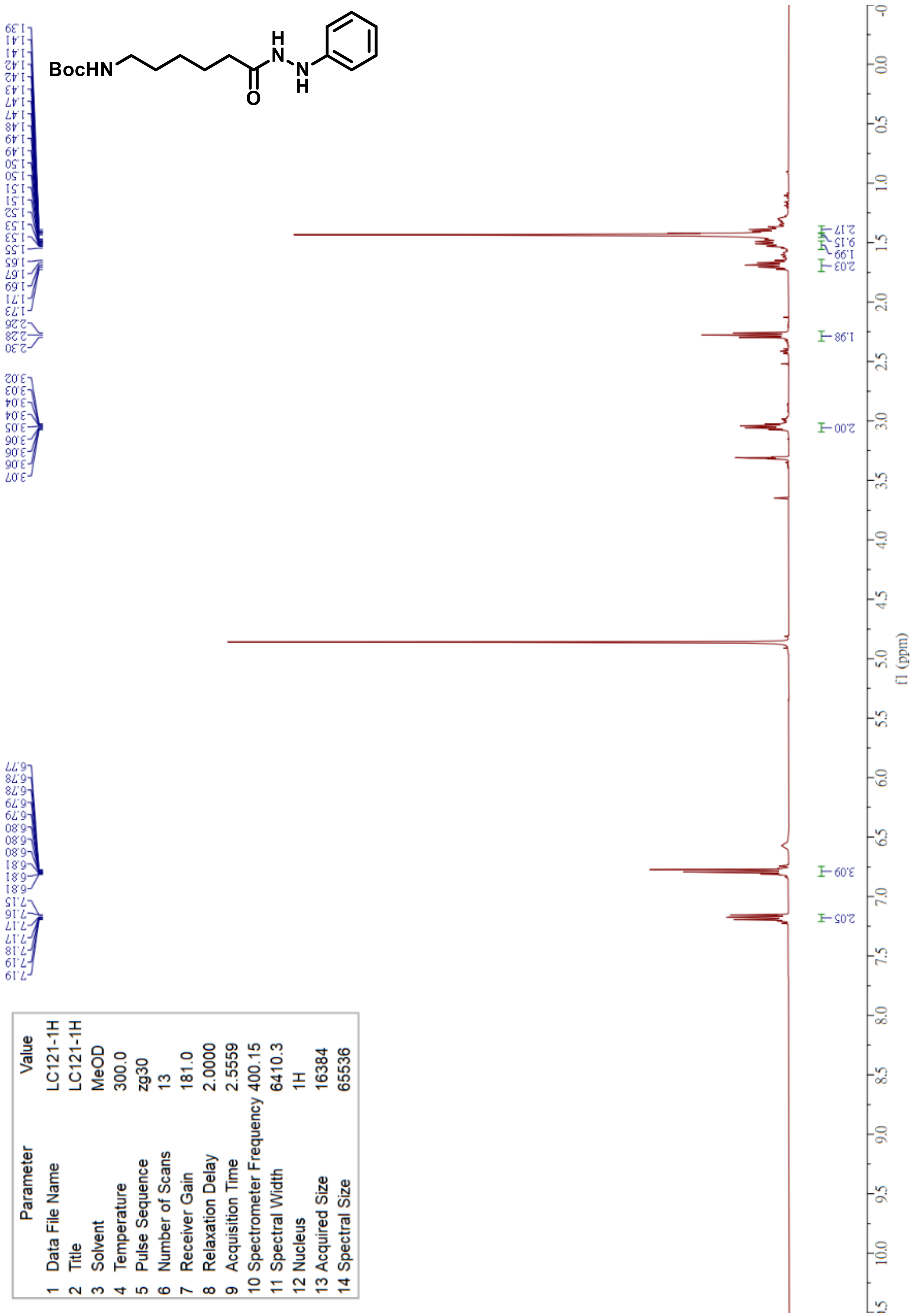

Figure S47 ^{1}H NMR spectra of compound **14**.

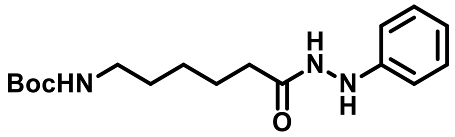

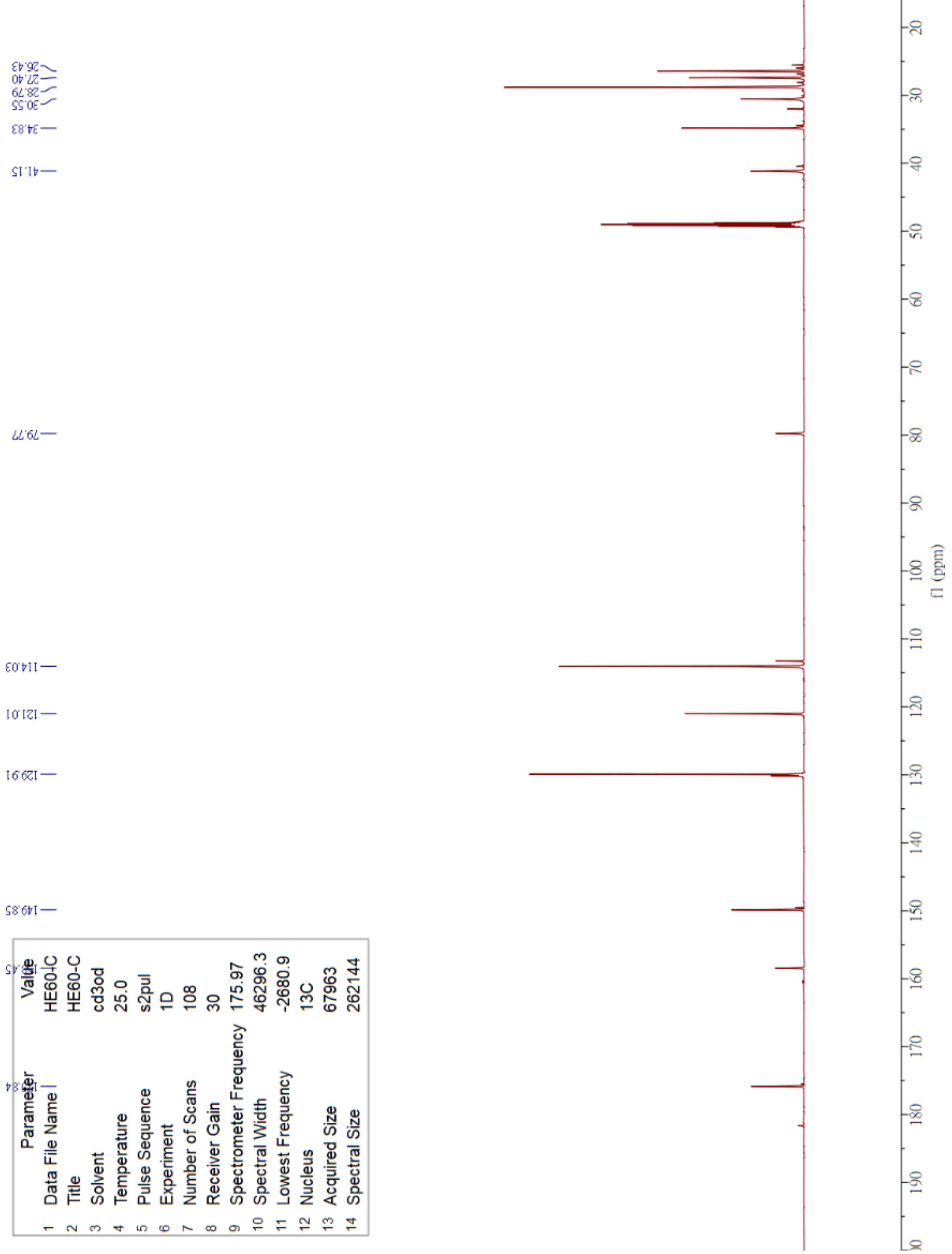

Figure S48 ^{13}C NMR spectra of compound **14**.

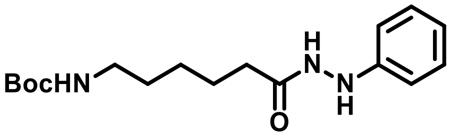

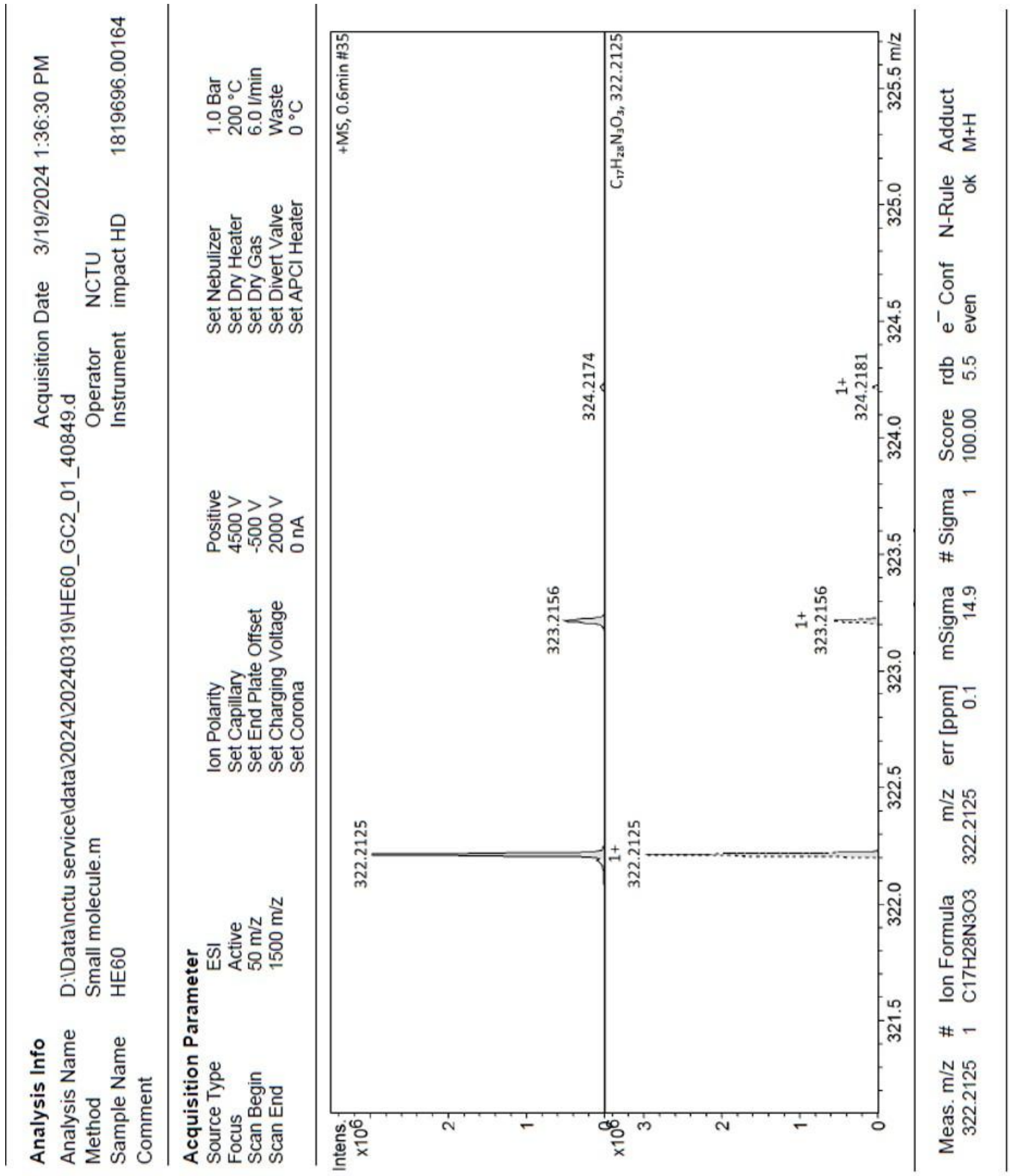

Figure S49 HRMS-ESI spectra of compound **14**.

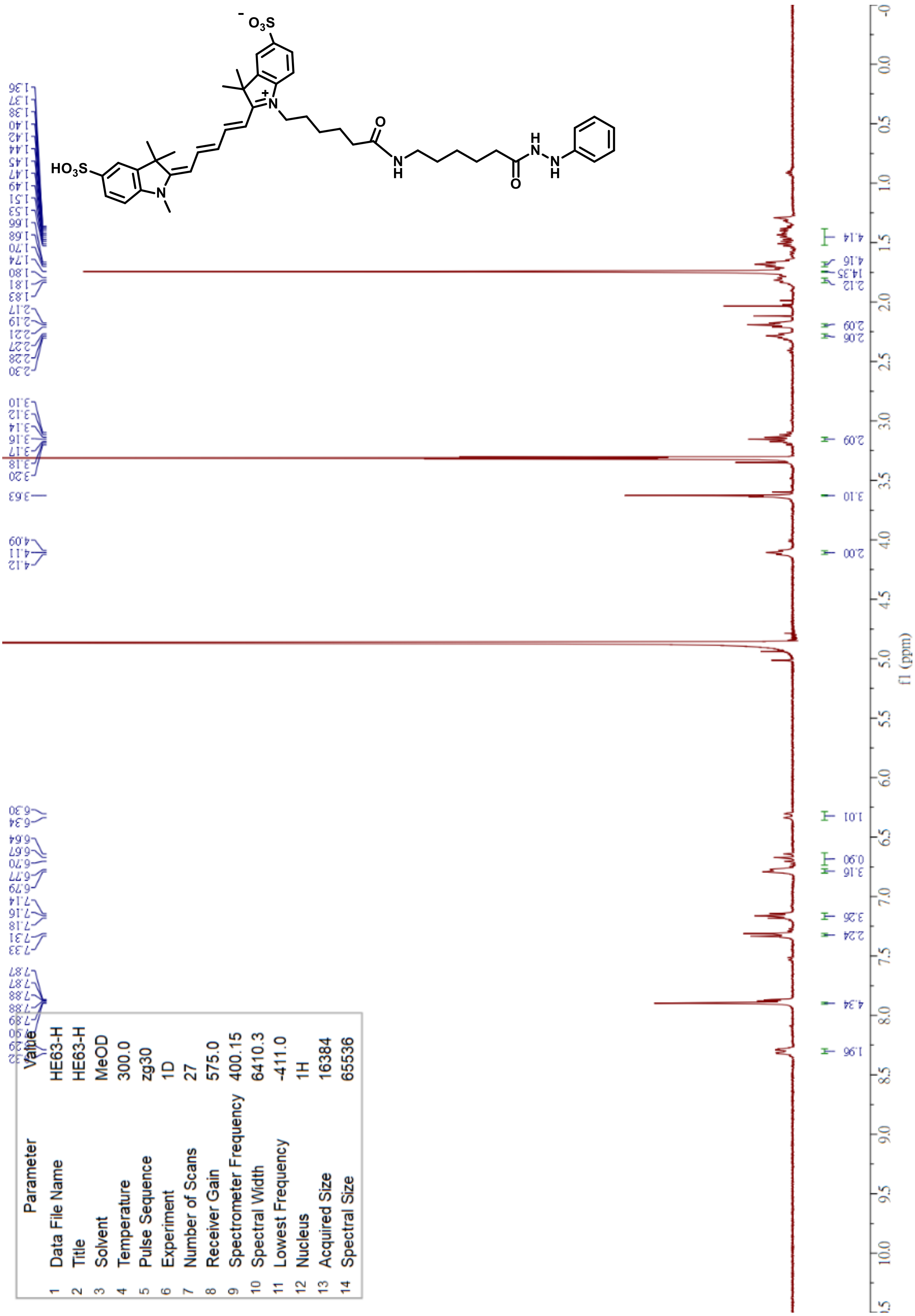

Figure S50 ^{1}H NMR spectra of **Probe 2**.

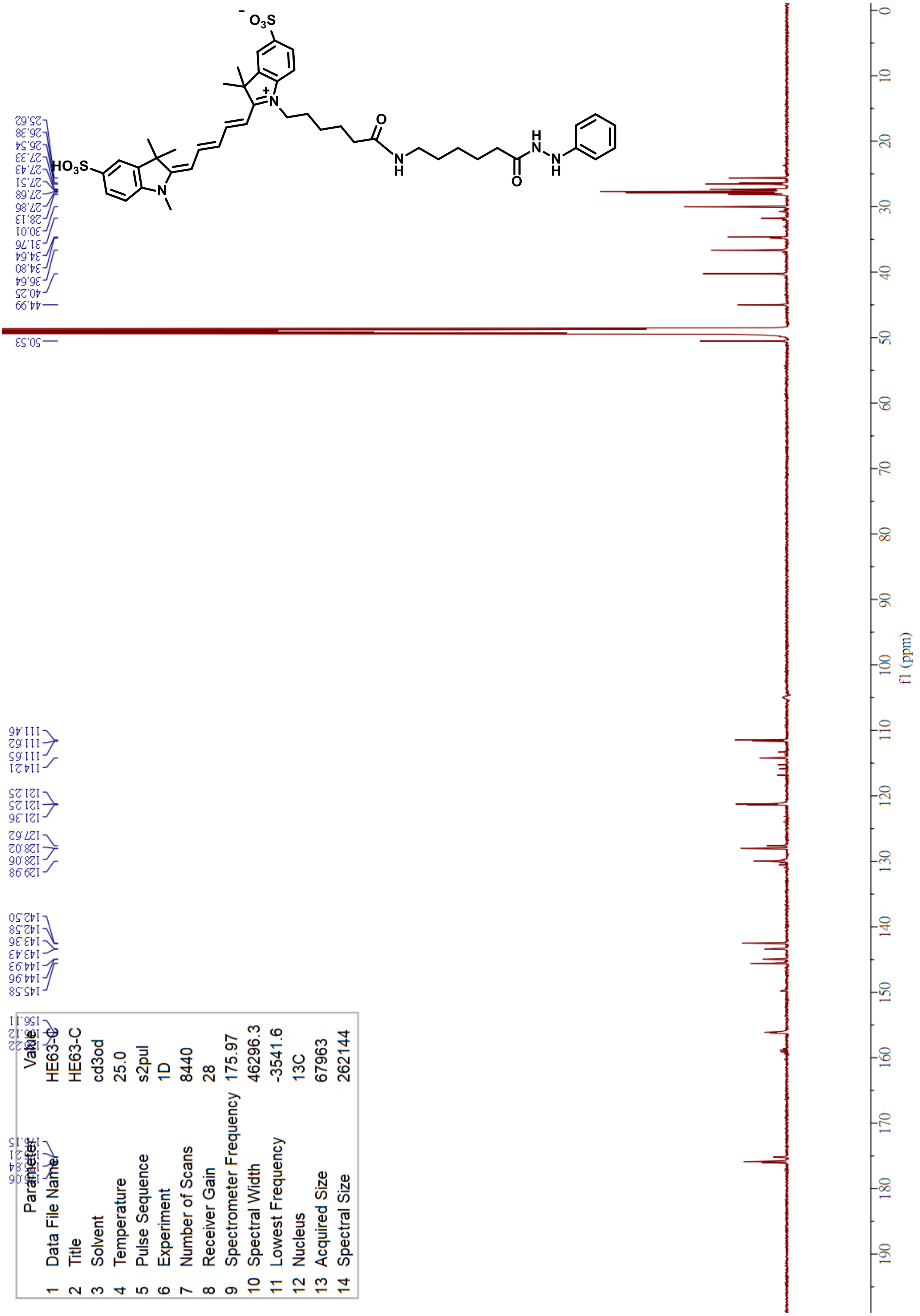

Figure S51 ^{13}C NMR spectra of **Probe 2**.

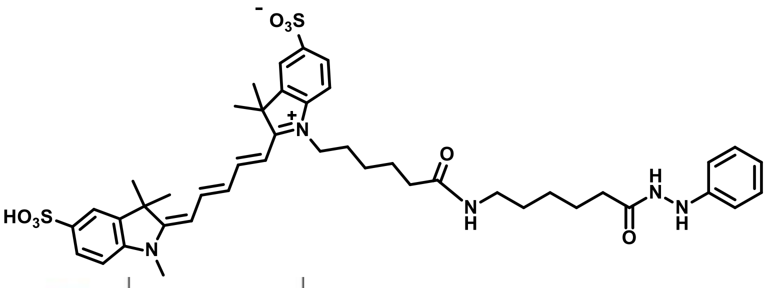

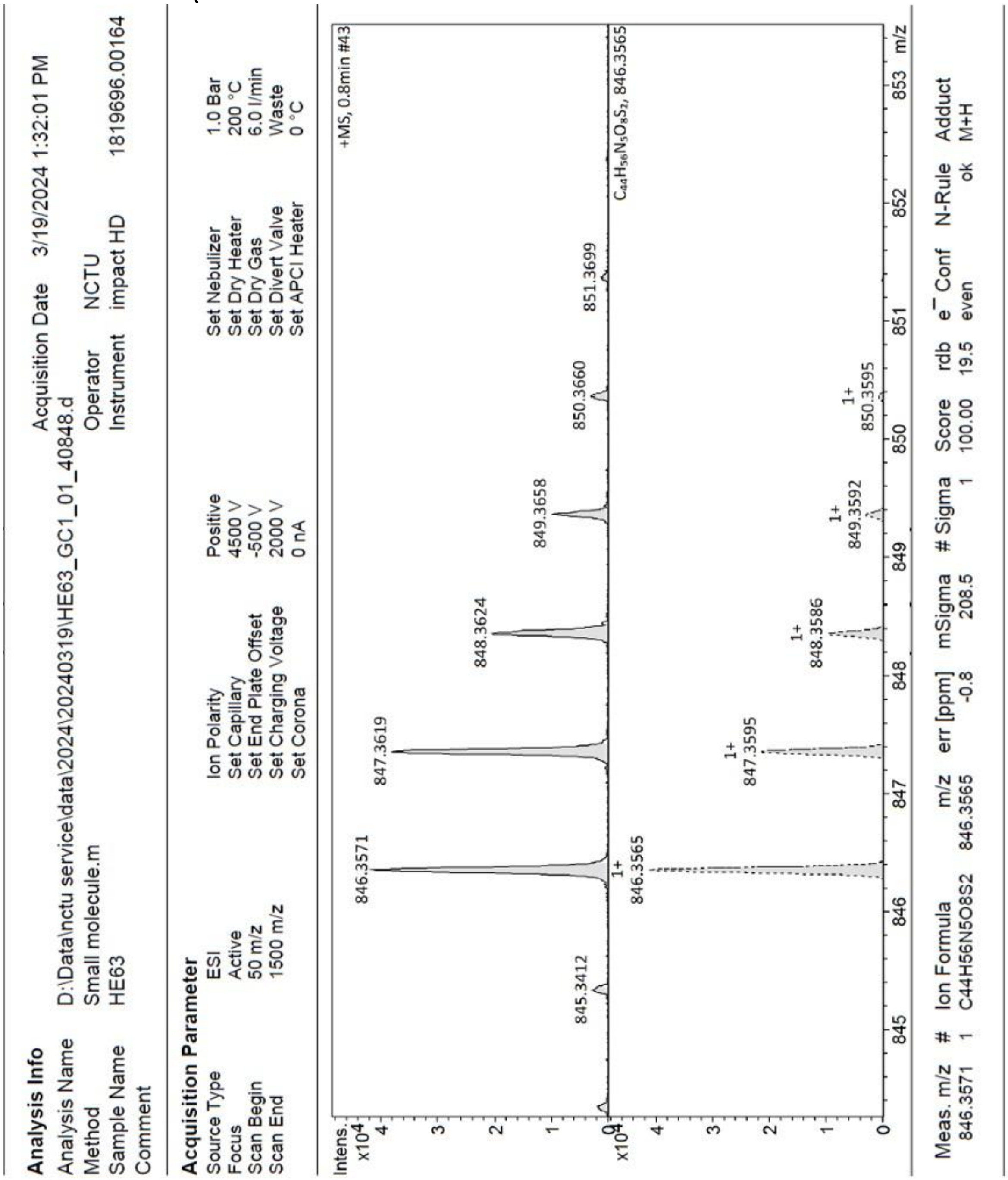

Figure S52 HRMS-ESI spectra of **Probe 2**.

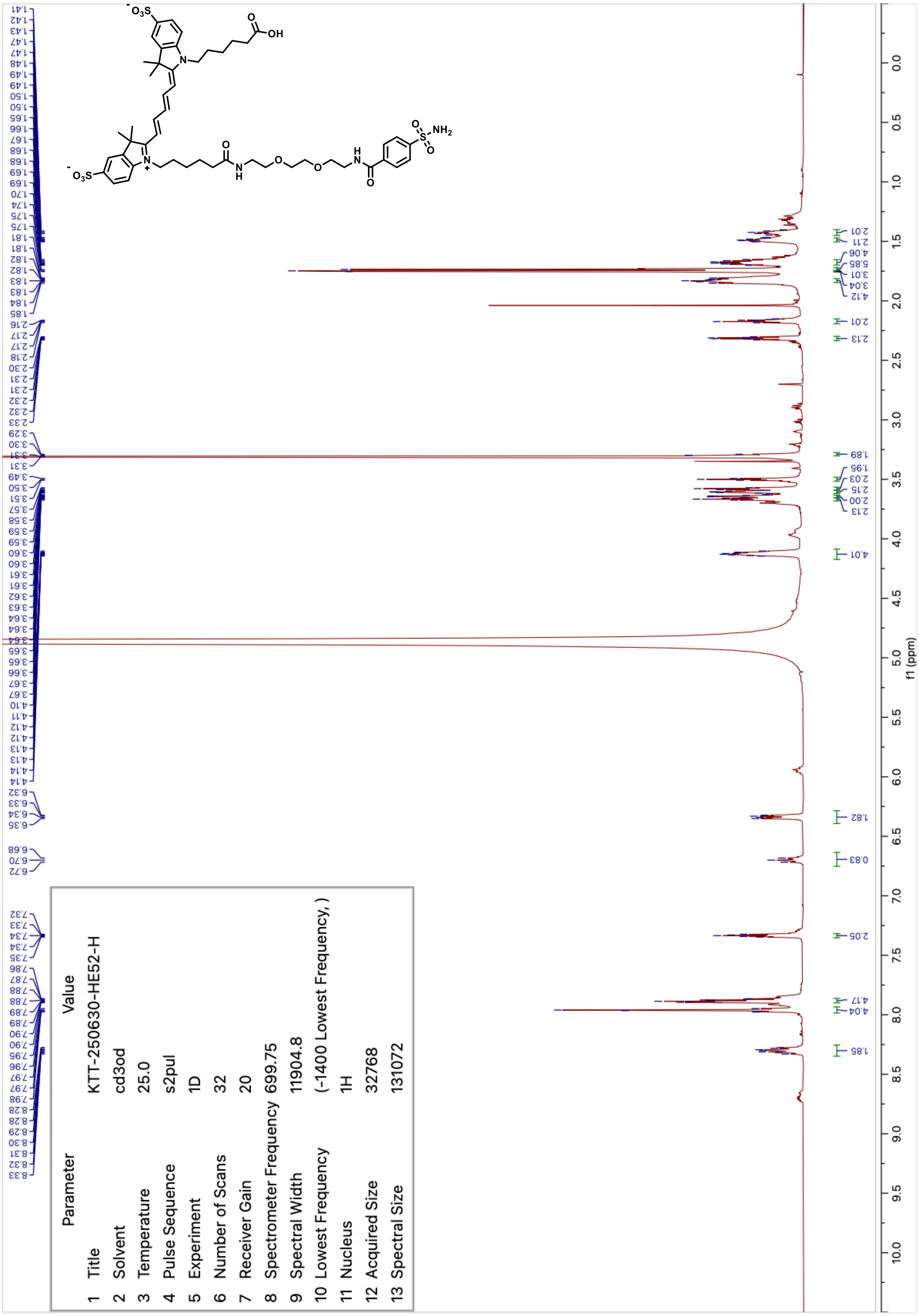

Figure S53 ^{1}H NMR spectra of **Probe 3**.

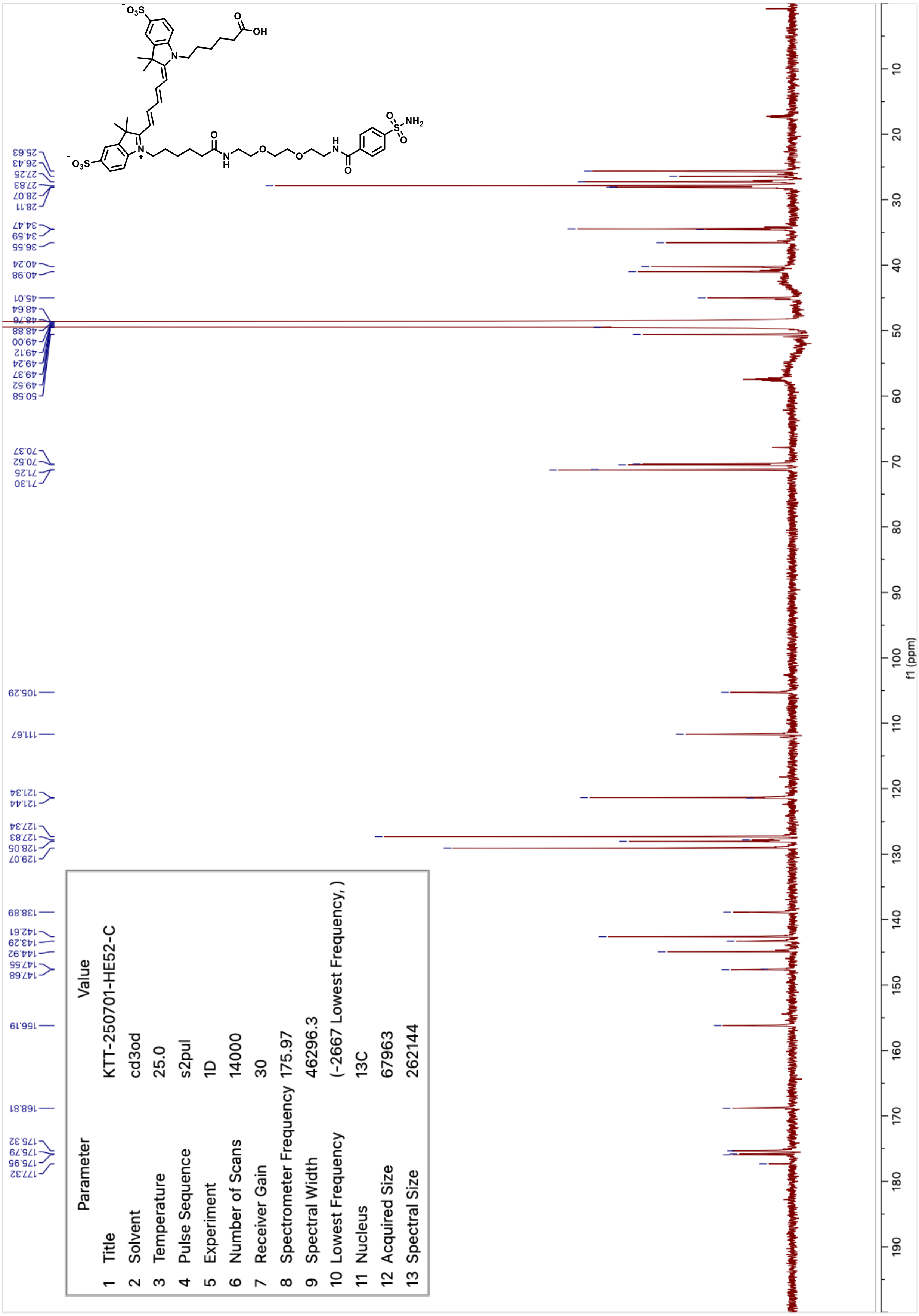

Figure S54 ^{13}C NMR spectra of **Probe 3**.

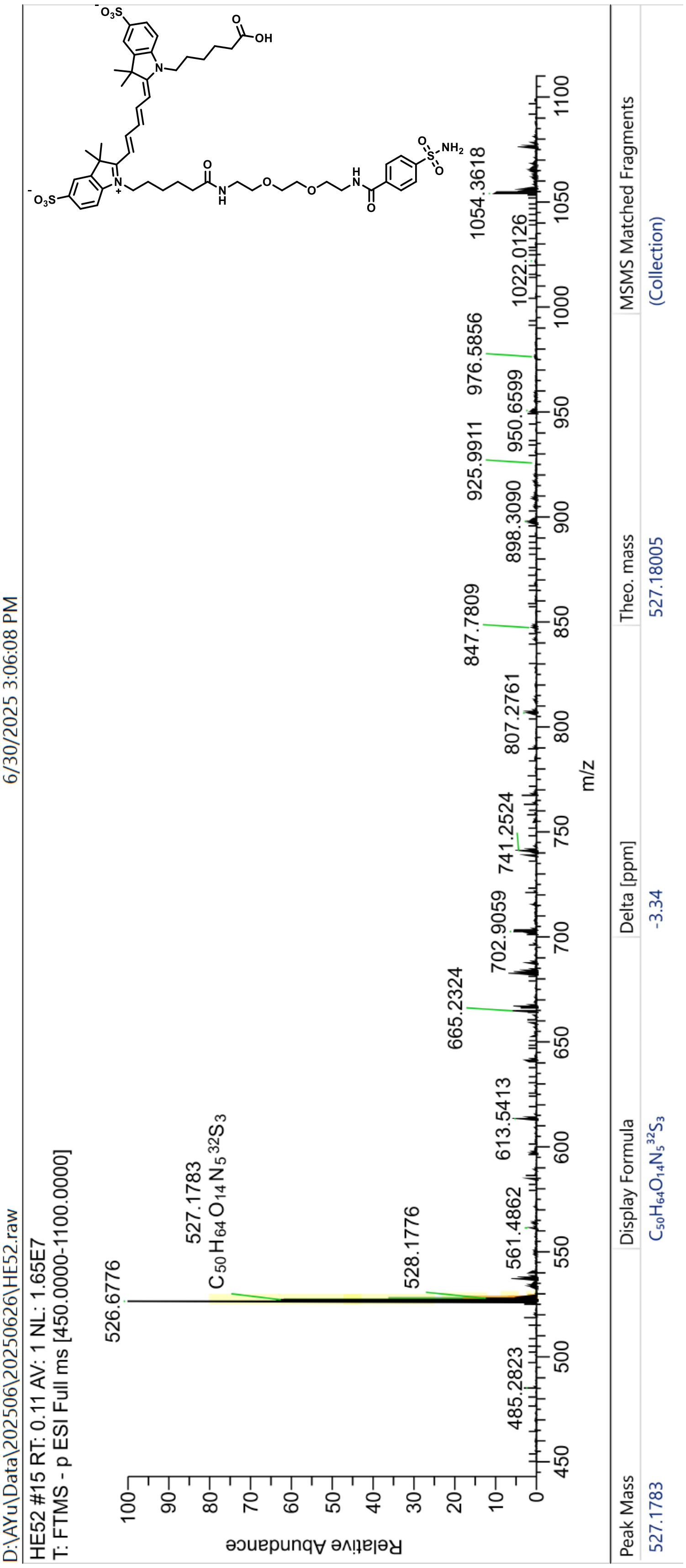

Figure S55 HRMS-ESI spectra of **Probe 3**.
